# Genome instability is associated with ethnic differences between Asians and Europeans in hepatocellular carcinoma

**DOI:** 10.7150/thno.71676

**Published:** 2022-06-06

**Authors:** Neslihan A. Kaya, Jianbin Chen, Hannah Lai, Hechuan Yang, Liang Ma, Xiaodong Liu, Jacob Santiago Alvarez, Jin Liu, Axel M. Hillmer, David Tai, Joe Yeong Poh Sheng, Zheng Hu, Yun Shen Chan, Pierce K.H Chow, Yuguang Mu, Torsten Wuestefeld, Weiwei Zhai

**Affiliations:** 1 Genome Institute of Singapore, Agency for Science, Technology and Research, Singapore, 138672, Singapore.; 2 Key Laboratory of Zoological Systematics and Evolution, Institute of Zoology, Chinese Academy of Sciences, Beijing, 100101, China.; 3 School of Biological Sciences, Nanyang Technological University, Singapore, 637551, Singapore.; 4 Centre for Quantitative Medicine, Program in Health Services and Systems Research, Duke-NUS Medical School, Singapore, 169857, Singapore.; 5 Institute of Pathology, Faculty of Medicine and University Hospital Cologne, University of Cologne, Cologne, 50937, Germany.; 6 Division of Medical Oncology, National Cancer Centre Singapore, Singapore, 169610, Singapore.; 7 Singapore General Hospital, Singapore, 169608, Singapore.; 8 Institute of Molecular and Cell Biology, Agency for Science, Technology and Research (A*STAR), Singapore, 138673, Singapore.; 9 CAS Key Laboratory of Quantitative Engineering Biology, Shenzhen Institute of Synthetic Biology, Shenzhen Institutes of Advanced Technology, Chinese Academy of Sciences, Shenzhen, 518055, China.; 10 Guangzhou Laboratory, Guangzhou International Bio Island, Guangzhou 510005, Guangdong Province, China.; 11 Hepatopancreatobiliary and Transplant Surgery, Singapore General Hospital, Singapore, 169608, Singapore.; 12 Division of Surgery and Surgical Oncology, National Cancer Centre, Singapore, 169610, Singapore.; 13 Office of Clinical Sciences, Duke-NUS Graduate Medical School, Singapore, 169857, Singapore.; 14 Center for Excellence in Animal Evolution and Genetics, Chinese Academy of Sciences, Kunming, 650223, China.; 15 School of Life Sciences, Division of Life Sciences and Medicine, University of Science and Technology of China, Hefei 230027, China.

## Abstract

Hepatocellular carcinoma (HCC) is one of the deadliest cancer types with diverse etiological factors across the world. Although large scale genomic studies have been conducted in different countries, integrative analysis of HCC genomes and ethnic comparison across cohorts are lacking.

**Methods:** We first integrated genomes of 1,349 HCC patients from five large cohorts across the world and applied multiple statistical methods in identifying driver genes. Subsequently, we systematically compared HCC genomes and transcriptomes between Asians and Europeans using the TCGA cohort.

**Results:** We identified 29 novel candidate driver genes, many of which are infrequent tumor suppressors driving late-stage tumor progression. When we systematically compared ethnic differences in the genomic landscape between Asian and European HCCs using the TCGA cohort (n = 348), we found little differences in driver frequencies. Through multi-modal integrative analysis, we found higher genomic instability in Asians together with a collection of molecular events ranging from tumor mutation burden (TMB), copy number alterations as well as transcriptomic subtypes segregating distinctively between two ethnic backgrounds. Strikingly, we identified an Asian specific transcriptomic subtype with multiple ethnically enriched genomic alterations, in particular chromosome 16 deletion, leading to a clinically aggressive RNA subgroup unique to Asians. Integrating multi-modal information, we found that survival models predict patient prognosis much better in Asians than in Europeans, demonstrating a higher potential for precision medicine applications in Asia.

**Conclusion:** For the first time, we have uncovered an unprecedented amount of genomic differences segregating distinctively across ethnicities in HCC and highlighted the importance of differential disease biology and management in HCC across ethnic backgrounds.

## Introduction

Hepatocellular carcinoma (HCC) is the major subtype of liver cancer and ranks fourth in the cancer related deaths 1. Major risk factors for HCC include viral infection, alcohol intake as well as environmental exposures (*e.g.* aflatoxin) which segregate distinctively across different geographic regions of the world 2,3. Previous studies with medium size cohorts have characterized HCC genomes from a wide range of ethnic backgrounds including Japanese 4, Korean 5, French 6 and the US cohorts 7. Common molecular events, including driver genes (*e.g. CTNNB1*), functional pathways (*e.g.* the Wnt pathway) as well as transcriptomic subtypes have been extensively characterized in HCC 8,9.

Despite rapid progress in understanding HCC genomes, there are still significant gaps in the field. First, even though major molecular changes including common driver genes have been discovered using medium size cohorts, a significant proportion of HCC patients do not carry any known driver mutations (*e.g.* ~20% in the TCGA cohort 7), indicating insufficient power in identifying less frequent driver events. Secondly, given diverse etiological backgrounds in HCC, molecular events have only been explored individually in each cohort and findings are often partially consistent 10. Due to the shortage of multi-modal datasets collected using the same sequencing protocol across cohorts, systematic comparison between different ethnic backgrounds has not been explored in the field. Lastly, even though integrative survival analysis has been explored in each cohort separately, a systematic integration of multi-layer information and comparison across cohorts has not been explored 11,12.

In this study, we first performed an integrative genomic analysis of five large HCC cohorts (n = 1,349 patients) and identified a significant number of novel candidate drivers using several statistical methods. In order to understand ethnic differences between Asians and Europeans, we conducted a systematic comparison across multiple genomic layers using the TCGA cohort and identified a suite of genomic events segregating differentially between ethnic backgrounds.

Through integrative survival analysis, we combined ethnically different factors in patient stratification models and compared their performances across ethnic backgrounds. For the first time, we uncovered an unprecedented amount of ethnic differences in HCC and highlighted the importance of studying differential disease biology and management across ethnic backgrounds.

## Results

### Ethnic differences in clinical phenotypes in the TCGA cohort

Even though many cohorts were collected for studying HCC genomes 4,5,7,10,13, they often have a single layer of genomic data (*e.g.* DNA changes) from a particular ethnic background. Thus, ethnic differences in HCC have not been systematically explored, partly due to lack of a suitable dataset with multi-modal information collected using the same protocol. The TCGA cohort which includes multi-layered genomic data from similar number of patients from both ethnicities (161 Asian and 187 European patients) is an ideal cohort for ethnic comparison. In order to conduct systematic comparisons, we reprocessed the raw sequencing data downloaded from the Genomic Data Commons (GDC) portal using our in-house pipeline (see Methods) and compared the two cohorts across multiple layers.

**Figure 1:**
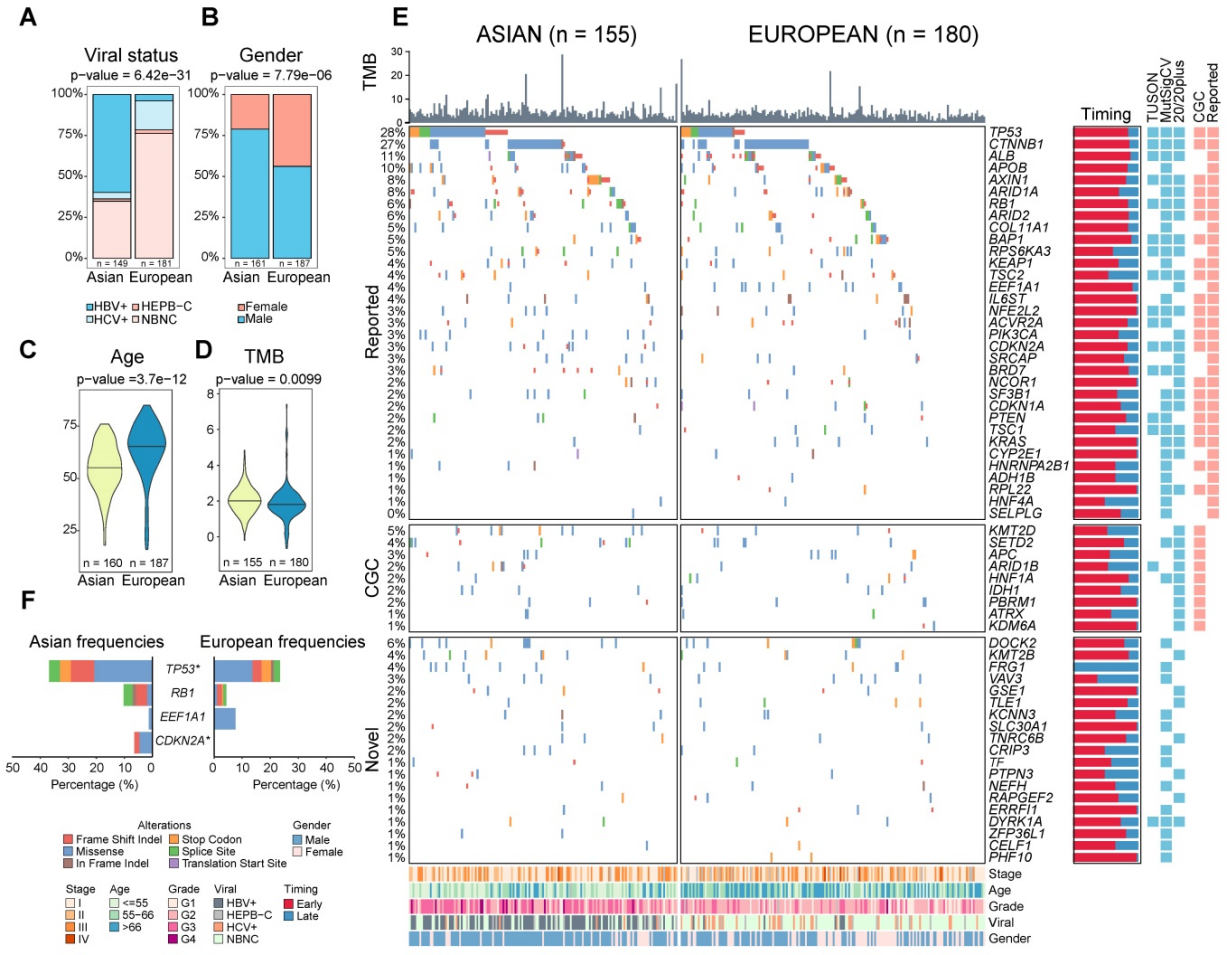
** Comparison of clinical and genomic profiles between Asian and Europeans.** Ethnic differences were found in clinical phenotypes including A) viral status, B) gender, C) age, D) TMB between Asians and Europeans. E) The driver gene landscape of Asian and European HCCs were shown. For the reported drivers, only the ones with frequencies greater than 5% were shown in this oncoprint plot. The plot on the right side indicates proportions of early (red) and late (blue) mutations in the driver genes across patients. Heatmaps on the right-hand side indicate whether the driver gene is detected by different methods or whether the gene was previously reported by other studies (reported) or in the cancer gene census list (CGC). Clinical phenotypes of patients were shown at the bottom of the panel. F) Driver genes with significantly different frequencies between Asian and European cohorts (Fisher's Exact test p-value < 0.05). The star indicates a q-value of less than 0.1 after multiple testing correction. Mutation types were shown in different colors.

Comparing clinical variables between two cohorts, the most significant difference was the viral status (p = 6.42e^-31^, Figure 1A). While around 60% of Asian patients are HBV positive, only 25% of Europeans are viral carriers. In addition, European patients have a relatively higher proportion of female patients (44% vs 21%, p = 7.79e^-06^, Figure 1B) and older age at diagnosis (median age 66 vs 55, p = 3.66e^-12^, Figure 1C). In general, the two cohorts are similar in other clinical phenotypes including tumor stage, microvascular invasion (MVI) as well as tumor purity (Figure S1A-S1D).

### Similar driver frequencies across ethnic backgrounds, but higher TMB in Asians

To evaluate ethnic differences in the genomic landscape across multiple layers, we first compared the tumor mutation burden (TMB) between two ethnic backgrounds and found a significantly higher TMB in Asian patients (p = 9.90e^-03^, Figure 1D). This difference remains significant after controlling for clinical variables (*e.g.* viral status) and tumor purity (p = 4.58e^-03^, Figure S2). Higher TMB in Asians raised an interesting question whether two cohorts will also differ in other molecular phenotypes. Driver genes play a very important role in driving multi-stage tumorigenesis 14, but were mainly identified using cohorts from single ethnic background 4-7,10,15. When we compiled a list of genes (n = 88) from eight previous studies, 76% of these drivers are discovered by only a single study (Figure S3, Table S1). In order to identify a comprehensive list of drivers, we collected HCC genomes from five large cohorts including The Cancer Genome Atlas (TCGA, n = 373), International Cancer Genome Consortium (ICGC) database 16 (n = 270 for Japanese from Riken: LIRI-JP, n = 244 for Japanese from National Cancer Center: LINC-JP and n = 242 for French: LICA-FR), as well as a Korean cohort (n = 231) (Table S2-S3). Leveraging the large sample size (n = 1349, Supplementary Note 1), we integrated three different methods 17-19 and identified 62 candidate driver genes for HCC (q-value < 0.1, Supplementary Note 1, Figure S4-S6, Table S4-S5).

Among 62 candidate drives, 33 genes (53%) overlapped with literature reported driver list. 29 novel candidate drivers which include several interesting candidate genes such as *DOCK2* (a gene frequently mutated in esophageal adenocarcinoma and colorectal carcinoma 20,21) were identified (Figure S5-S6, Table S5). A full list of drivers and their functional roles are discussed in the Supplementary Note 1. Further analysis revealed several important findings about driver genes in HCC: 1) The association between driver genes and clinical phenotypes (*e.g.* viral status or ethnicity) is rather weak (Figure S6C), suggesting that driver genes may be independent of disease etiology; 2) Drivers from different pathways tend to co-occur while drivers from the same pathway often mutate “mutual exclusively” (Figure S6D, Supplementary Note 1). 3) The list of novel driver candidate genes was enriched in a number of novel and known pathways (Figure S6E). Even with the large sample size, the number of driver genes is far from saturation (Figure S7A, Supplementary Note 2). Moreover, less frequent novel drivers tend to occur late (subclonal) in the history of tumorigenesis and the chromatin remodeling pathway is enriched for late drivers (Figure S7B-E, p = 8.23e^-11^), suggesting that there are many rare driver genes driving tumor progression that have not been identified yet in HCC. 4) Many new candidate drivers are potential tumor suppressor genes with high levels of truncating mutations (n = 45, Figure S7F, S7G, Table S6, Supplementary Note 3).

Leveraging the large number of driver genes identified using public cohorts, we systematically compared driver frequencies between Asian and European patients in the TCGA cohort. Surprisingly, most of the drivers have similar frequencies except for *TP53* and *CDKN2A* (Figure 1E-F, q-value < 0.1, Table S7, Supplementary Note 1). Despite disparate etiological backgrounds between the two cohorts (*e.g.* viral status), driver gene profiles are rather similar between Asians and Europeans.

### Ethnic differences in the mutational process during tumorigenesis

**Figure 2:**
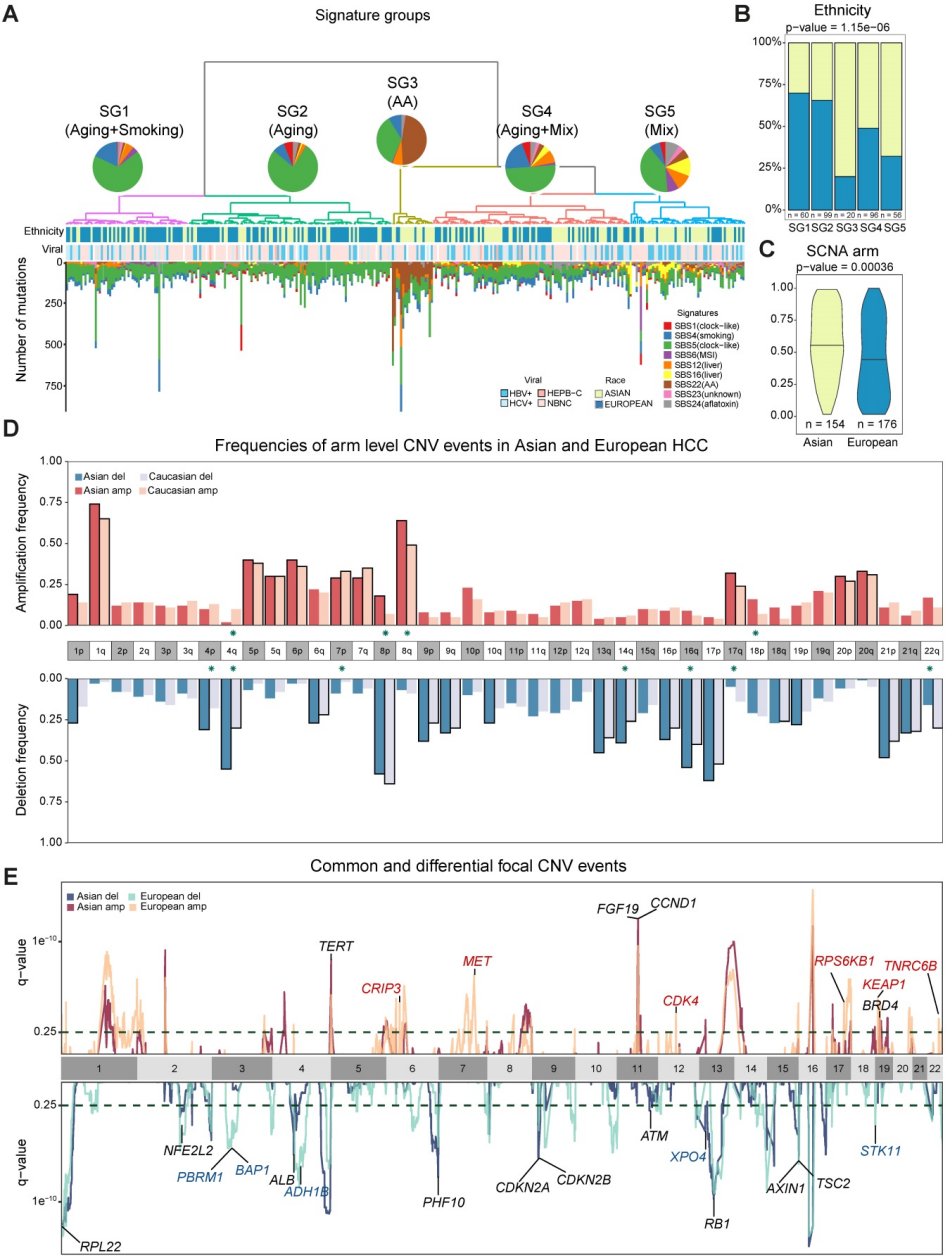
** Ethnic comparison of mutational signatures and copy number alterations.** A) Signature groups across patients. Mutations contributed by different mutational process were plotted as barplots across patients. The pie charts indicate the mean proportion of each mutational signature in each group. Ethnicity and viral status of patients were shown as annotation for each patient. B) Proportions of Asian and European patients in the signature groups. C) Comparison of arm level somatic copy number alteration (SCNA) scores in two cohorts. D) Arm level events in Asians (n = 154) and Europeans (n = 176). Frequencies for each arm are shown with different colors for Asians and Europeans. Arms with significant difference (*i.e.* p-value ≤ 0.05) were indicated with green stars around chromosome labels (n = 11). Chromosome arms with black borders indicate putative driver CNVs (based on GISTIC output). E) Focal CNV peaks for Asian (n = 154) and European (n = 176) cohorts. Driver genes within GISTIC peaks were labelled. Genes in common peaks are colored in black while genes in cohort specific peaks are colored in their respective colors.

The higher TMB and similar prevalence of driver genes raised an interesting question how different mutational processes could yield a different genomic landscape between the two cohorts. When we deconvolute mutations into contributions of known mutational signatures found in HCC (n = 10, Methods) 12,22,23 using deconstructSigs 24, all except one have appreciable proportions in the cohort (Supplementary Note 4, Table S8). Using the contributions of different mutational signatures, we clustered the patients into five signature groups (denoted as SG1-5, Figure 2A). Groups SG1 and SG2 are dominated by SBS5 (clock like) signature and are enriched for European patients (Figure 2B-2D, Figure S9C p = 1.15e^-06^). SG3 with strong aristolochic acid (*i.e.* AA) signature (SBS22) and higher TMB is much more frequent in Asian patients (Figure 2A-B, Figure S9G, Figure S10A). SG4 has a dominant signature of SBS5 together with an appreciable proportion of SBS4 (smoking) and a mix of other signatures (Figure S9B). SG5 has much higher frequency of liver related signatures (SBS12 and SBS16) and is also enriched for Asian patients (Figure 2A-B, Figure S8E-F, Figure S9E-F, p-value = 1.15e^-06^). It is important to note that signatures groups did not correlate with viral status of patients (Figure 2A). Through timing and clonality analysis, we found that signatures related to external exposures such as smoking and AA are significantly lower in the late stage of tumorigenesis while MSI and liver associated signatures have higher proportions in the late stages of tumorigenesis, suggesting their active role throughout HCC initiation and progression (Supplementary Note 4, Figure S10C). Even though the prevalence of the mutational signatures differs between the two cohorts, the evolutionary timing of common signatures is quite similar across the two cohorts (Figure S10C-D).

### Chromosomal CNVs drive higher genome instability in Asians

While point mutations and mutational signatures point to a higher genomic instability in Asians, copy number alterations (CNAs) are the other important mutational process driving tumorigenesis. Using Somatic CNA (SCNA) score, which integrates both the magnitude as well as the scale of CNAs, we found that Asians have higher arm level SCNA (Figure 2C, p = 0.00036). After controlling for clinical variables as well as other covariates, ethnic differences in arm level SCNA score remained significant (p = 0.02, Figure S11-S13). Breaking down the overall SCNA scores into contributions of individual chromosomes, 11 arms (4 amplifications and 7 deletions) including chromosome 16 deletions and 8q amplification were altered at significantly different frequencies between the two cohorts and were mostly enriched in Asians (Figure 2D, Fisher's Exact test q-value < 0.1). In addition to arm level differences, when we compared focal CNAs using the GISTIC algorithm, the landscape stay qualitatively similar between cohorts (*e.g. TERT* and *FGF19* amplification and *AXIN1* deletions 5,25, Figure 2E, Table S9, Figure S14), despite the existence of private peaks to each cohort. In summary, HCC in Asians have significantly higher genome instability contributed by multiple arm level CNV events.

### A clinically more aggressive transcriptomic subtype unique to Asia

**Figure 3:**
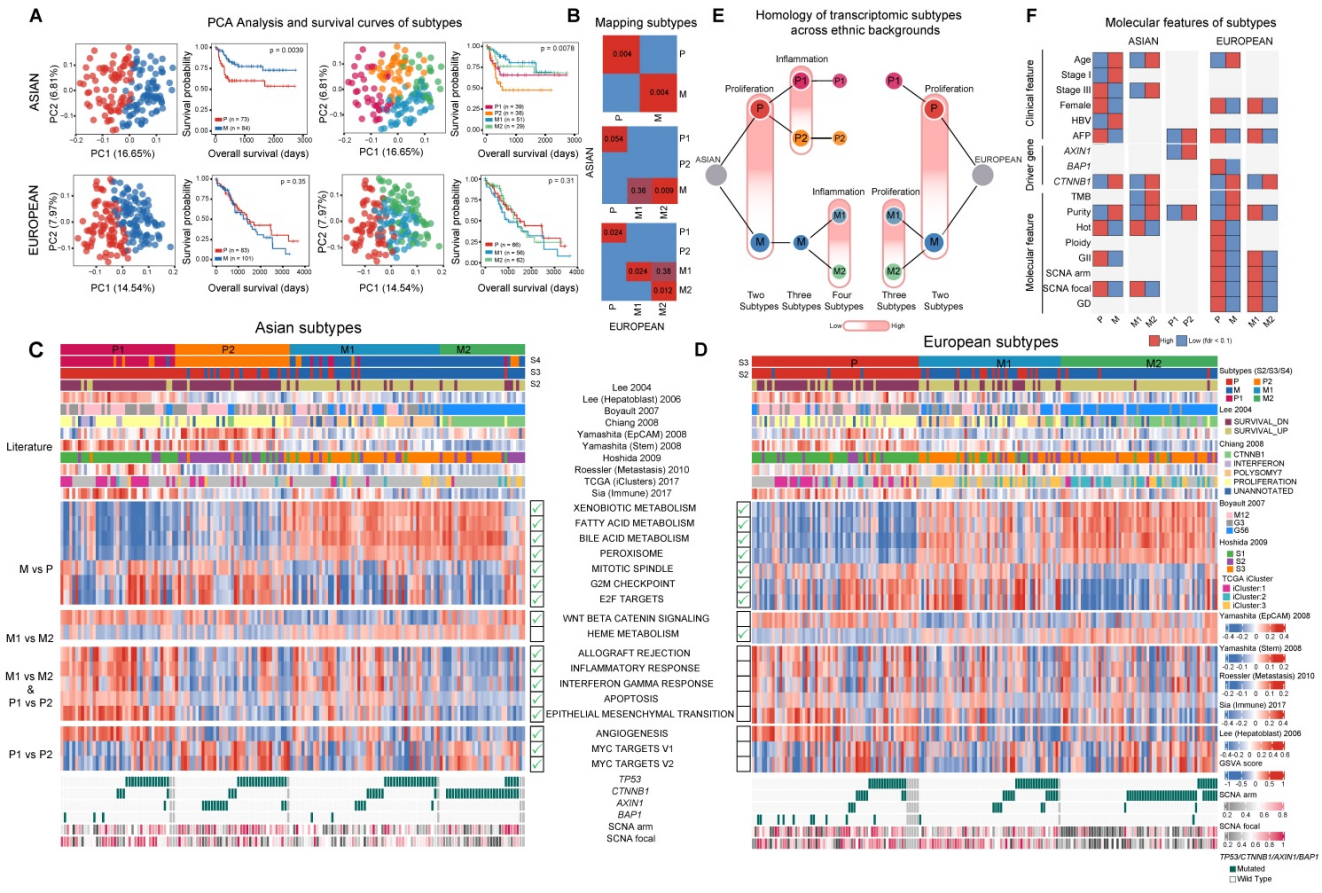
** Transcriptomic landscape between Asian and European cohorts.** A) Principal component and survival analysis when partitioning the two cohorts into two, three and four subtypes. Results for two subtypes are shown for both cohorts. Optimal number of subtypes (4 for Asians and 3 for Europeans) are shown for both cohorts. B) Pairwise similarity mapping of subtypes between Asian and European cohorts using the SubMap method. c-d) Heatmaps displaying differentially expressed pathways between different transcriptomic subtypes for Asian (n = 158) (C) and European (n = 184) (D) cohorts. Annotations on top of each heatmap show subtypes reported by the current work and previous studies. Bottom rows display subsets of differentially expressed pathways. Green tick marks indicate the significance of pathways in the Asian or European cohort. E) Homologous relationship between transcriptomic subtypes between the two cohorts. The color hue indicates the upregulation of the key pathways delineating the subtype partition (*e.g.* proliferation or inflammation). F) Significant differences in clinical features, driver genes as well as molecular features across subtype comparisons**.** (GII: genome instability index, GD: genome doubling).

With higher genome instability found in Asians, we wondered whether higher genomic instability could drive phenotypic divergence, especially transcriptomic differences between ethnic backgrounds. A literature review revealed a list of transcriptomic subtypes (n = 7) with varying levels of consistency between multiple cohorts 26-35 and differences between Asians and Europeans have not been systematically explored in these studies. Using non-negative matrix factorization (NMF) 36, we first clustered the Asian and European cohorts from TCGA into two subtypes (Figure 3A, Figure S15) and compared the subtype similarity using SubMap 37 (Figure 3B). Interestingly, in both cohorts, we observed one subgroup with upregulated cell cycle (*e.g.* “G2M checkpoint”), but down-regulation of metabolic pathways typical to common liver function (*e.g.* “Bile acid metabolism”) (Figure 3C-D). Even though the basal split is functionally similar across cohorts, the two-group subtyping only stratifies overall survival in the Asian cohort. We named basal clusters as P (proliferation) and M (metabolism) according to the activated pathways in basal partition. When we further cluster the two cohorts into three subgroups, the proliferation group (P) in Asians and the metabolism group (M) in Europeans further partitioned into two groups with the number of matching subgroups remaining at two (Figure 3B, 3E). The P1 subtype in Asians shows upregulation of EMT, inflammatory response, as well as angiogenesis pathways (Figure 3C, 3E, Figure S16A), while P2 has higher regulation of unfolded protein response (UPR) as well as MYC target genes (Figure S16C). The phenotypic divergence between the M1 and M2 subtype in Europeans is similar to the basal phenotypic divergence between P and M where M1 has higher cell cycle activity, but down regulated metabolic functions (Figure 3C-D and Figure S16B).

Using several statistical procedures for selecting the optimal number of clusters, the best number of subgroups for Asians and Europeans were found to be 4 and 3 respectively (Figure S15A-S15B). Partitioning Asians into four subgroups, the metabolism group further split into M1/M2, with the M1/M2 difference similar to the P1/P2 divergence with M1 having higher expression of immune related pathways as well as EMT. When comparing the four subtypes from Asians and three subtypes from Europeans, the two subgroups within the metabolism group (M1 and M2) match well between the two ethnic backgrounds and there is an extra subgroup (P2) unique to Asians (Figure 3B, 3E). Across all the clustering analysis, RNA subgroups stratify overall survival of patients very well in Asians, but not in Europeans (Figure 3A). Correlating transcriptomic subtypes with clinical and molecular phenotypes, we found a few clinical phenotypes such as alpha-fetoprotein (AFP) levels are enriched in subtype P in both cohorts and *CTNNB1* driver mutations are enriched in the M2 subtype in both cohorts (Figure 3F). Mapping previous transcriptomic subtypes together with the molecular events onto the subtype ontology, we found both concordant and divergent events across the two cohorts (Figure S16D, Supplementary Note 5).

### Genomic changes enriched in Asians delineating the transcriptomic subtype P2

**Figure 4:**
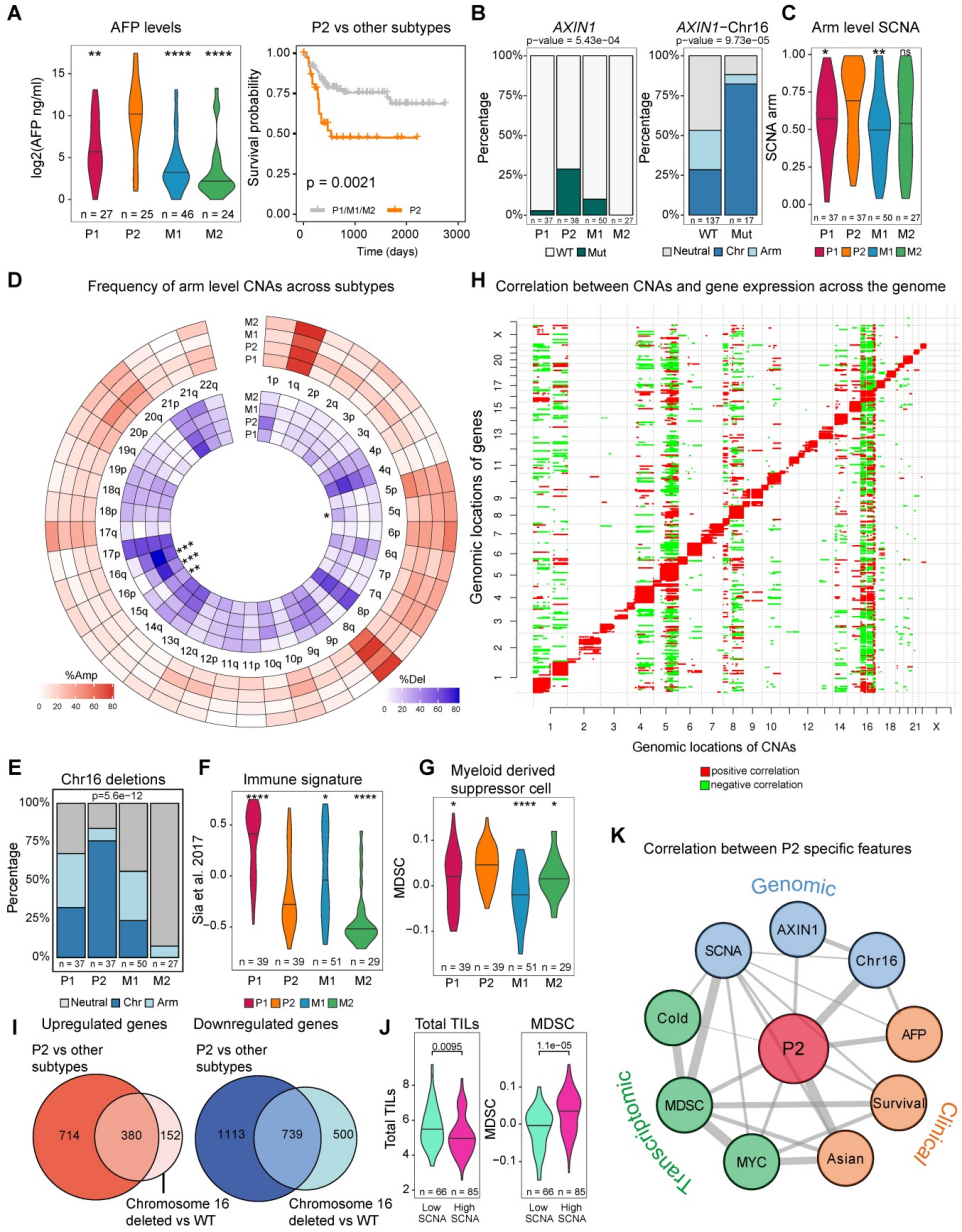
** Genomic features for an Asian specific subtype P2.** A) Alpha-fetoprotein (AFP) levels across transcriptomic subtypes and Kaplan-Meier survival curve for P2 and other transcriptomic subtypes in Asians. B) *AXIN1* mutations across subtypes (left). P2 subtype has the highest frequency of *AXIN1* mutations. Co-occurrence of *AXIN1* mutations with chromosome 16 deletion (right). C) Arm level SCNA score comparison across subtypes. D) Frequencies of copy number alterations across transcriptomic subtypes. Stars indicate significant differences. E) Proportion of patients with arm level or chromosome level deletions at chromosome 16 across subtypes. F) Comparison of gene signature of the immune class derived from Sia et al. 92 G) Myeloid derived suppressor cell (MDSC) score across subtypes. H) Correlation between copy number alterations (x axis) and mRNA expression (y axis) across the genome. Red color represents a significant positive correlation and green color indicates a significant negative correlation. I) Overlap between up-regulated and down-regulated genes when comparing P2 versus other subtypes and chromosome 16 deleted versus the rest of the patients (wild type or WT). J) Total tumor infiltrating lymphocyte (TIL, left) and myeloid derived suppressor cell (MDSC, right) levels between tumors with high and low SCNA tumors. K) Correlation network between P2 specific features across clinical, genomic as well as transcriptomic levels. Across all comparison, p-values ≤ 0.0001 were labelled as “****”, p-values ≤ 0.001 were labelled as “***”, p-values ≤ 0.01 were labelled as “**”, p-values ≤ 0.05 were labelled as “*” and p > 0.05 is “ns”.

The Asian-enriched transcriptomic subtype (P2) is one of the most aggressive subtypes with the highest level of AFP and the poorest survival (Figure 4A). This raised a series of interesting questions: what are the molecular events specific to this novel subtype and more importantly, are these subtype differences correlated with ethnic differences which might explain the origin of this ethnic specific subtype? Comparing genomic events between P2 and other subgroups, we found a series of genomic changes unique to P2: 1) significantly higher frequency of *AXIN1* mutations (Figure 3F, 4B), 2) strongly elevated SCNA as well as the highest level of CIN70 score 38 (Figure 4C, Figure S17B). When we break down the overall SCNA level into components, we found that chromosome 16 deletions were also significantly higher in P2 (Fig 4D) and tend to co-occur with* AXIN1* mutations (Fig 4B and 4E, p-value = 5.6e^-12^). 3) significantly higher expression of MYC targets and unfolded protein response (UPR), indicating endoplasmic reticulum (ER) stress possibly responding to fast cell cycle 39 (Figure S16C). 4) When we deconvolute the transcriptomic profile into immune components 40, we found that P2 and M2 are immunologically much colder than the other subtypes with lowest level of immune signature and P2 has the highest level of myeloid derived suppressor cells (MDSC) (Figure 4F-G). To understand whether the P2 subtype also exists in other Asian cohorts, we retrieved two Chinese cohorts 41,42 and assigned each patient to one of these four subtypes (Figure S18A-B). P2 was also found in these two cohorts and patients in the P2 subtype had similar phenotypes such as higher levels of AFP (Figure S18C-D), poor overall survival (Figure S18E) and higher frequency of chromosome 16 deletions (Figure S18F).

Despite a suite of genomic events highly enriched in P2, how these changes act concertedly to derive a new RNA subgroup is quite puzzling. Since ethnic differences are quite minor in driver frequencies, but a lot stronger in CNAs, we correlated copy number events with gene expressions across the genome. As expected, most of the CNVs act as cis-regulatory events, positively influencing the expression of genes in the genomic neighborhood (Figure 4H). Strikingly, CNV at chromosome 16 tends to impact expression levels of genes across the genome in the Asian cohort (Figure 4H) even though the correlation structure differs slightly between Asians and Europeans. (Figure S19). Moreover, differentially expressed genes (DEGs) found in the patients from the P2 subtype and DEGs found in patients with the chromosome 16 deletions are highly similar in the Asian cohort (Figure 4I), suggesting that the transcriptomic shift driven by chromosome 16 strongly correlates with the rise of the P2 subtype, which might explain the origin of this transcriptomic subtype.

In addition to chromosome 16 deletions, a suite of other genomic events defining P2 subtype seem to be acting collectively to define the P2 subtype. For example, previous studies reported that tumors with higher SCNA score tend to have lower immune infiltration across cancer types 43 and is also true in this HCC cohort (Figure 4J, p = 0.0095). Higher genomic instability including chromosome 16 correlates with low immune infiltration in P2 with high levels of MDSCs (Figure 4E, 4H, 4J) 43. When we draw a correlation network between P2 specific events across layers spanning clinical features, genomic changes, transcriptomic and immune phenotypes, we observe a well-connected network spanning multiple layers that defines the P2 subtype (Figure 4K). Taken together, ethnic differences in genome instability seem to drive a collection of genomic differences defining an Asian specific transcriptomic subtype.

### Integrative survival model predicts patient survival much better in Asians

**Figure 5:**
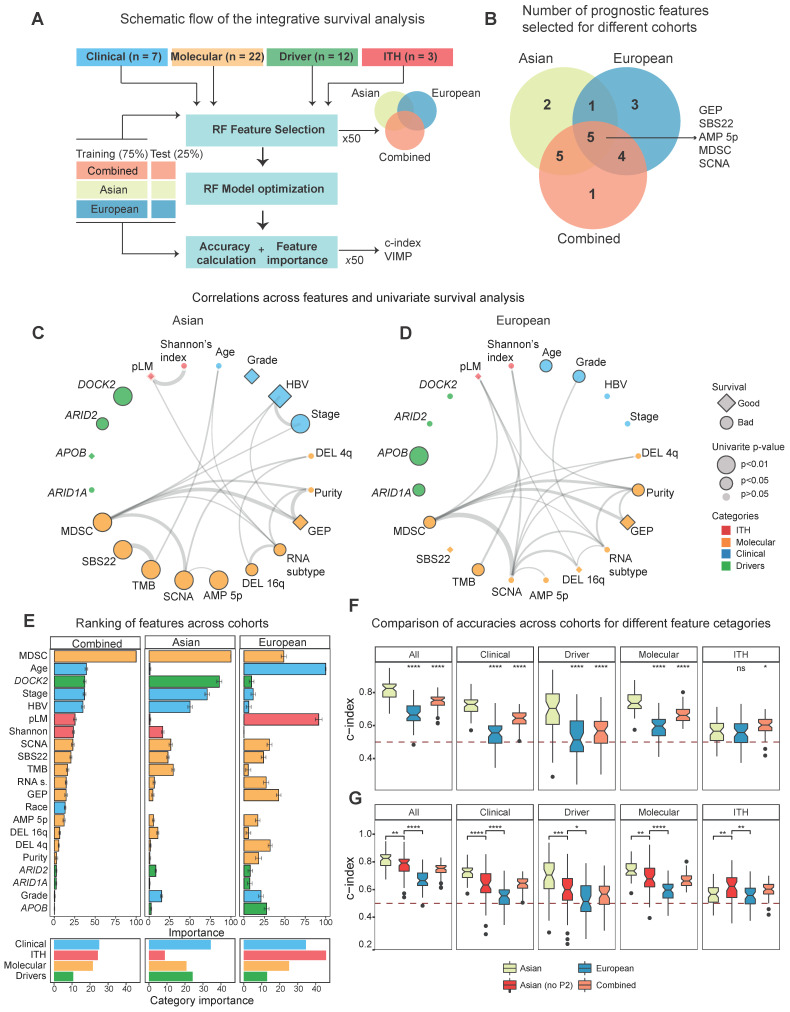
** Integrative survival analysis and ethnic differences.** A) A schematic summary of the integrative survival analysis. B) Number of significant features selected for Asian, European and the combined TCGA cohort. C-D) Correlation networks for the prognostic variables that can stratify patients in Asians (C) and Europeans (D). Edges of the network indicate significance of the correlation between features with the width of edges proportional to the re-scaled p-values (-log10(p-value)). Diamonds represent hazard ratios (HR) less than 1 (good prognosis) and circles represent HRs greater than 1 (poor prognosis). For features with multiple levels such as stage, HR of the most significant level was chosen. The black border around the nodes and size indicates its significance of the variable in the univariate Cox model. E) The ranking of importance for variables from clinical, molecular, driver and ITH categories. F) The predictive accuracy of the survival models when employing variables across different categories (All, Clinical, Driver, Molecular as well as ITH). Within each category, the Asian cohort was used as the reference group in the Wilcoxon test. G) The predictive accuracy of the survival models including the subset of Asian cohort without the P2 subtype.

With a large number of ethnic differences in HCC driven by genome instability, we wondered how ethnic differences might affect patient stratification and survival in two cohorts. In order to curate clinical and molecular features that can stratify patients, we collected multiple variables from different layers, including clinical phenotypes (*e.g.* stage, (n = 7), driver genes (n = 12) and other molecular features (n = 22). Since intra-tumor heterogeneity (ITH) has increasingly been recognized as an important factor driving patient clinical outcomes 11,44,45 and hasn't been explored in large cohorts for HCC 46-48, we curated three ITH metrics: 1) the percentage of late mutations (pLM), calculated as fraction of subclonal mutations, 2) Mutant-Allele Tumor Heterogeneity (MATH) score 49, measuring the distribution of variant allele frequencies, 3) Shannon's index, calculated based on the subclonal proportions (Methods). When we compared ITH values across Asians and Europeans, two cohorts had similar levels of ITH (Figure S20). In order to select variables that can stratify patients, random forest model was applied to Asian and European cohorts as well as the combined cohort (Figure 5A). It is interesting to observe that many variables that can stratify patients are shared between the two cohorts (Figure 5B, Figure S21).

When we calculate the correlation between features from multiple layers and plot the correlation network for the two cohorts separately, we found that multiple features strongly correlate with each other (Figure 5C-D, Table S10). While majority of selected features significantly stratify patients under the univariate Cox model (n = 17), a subset of features selected by random forest models were not significant in the univariate survival analysis (Figure 5C-D, Figure S22, Table S11), suggesting potentially non-linear relationship between these variables and patient's overall survival. For example, pLM stratifies patients when we categorize them as low, medium and high levels of each feature, but is not significant in the univariate model (Figure S23). By ranking the importance of these variables using random forest models (Methods), we found that immune features (*e.g.* MDSC) and driver genes (*e.g. DOCK2*) play very important roles in patient survival (Figure 5E, see Methods). Notably, ITH features rank rather poorly in the Asian cohort, but ranked first in the European cohort (Figure 5E, right-bottom). This high ranking of ITH features in the European cohort seem to reflect the poor prognostic ability across all variables in the cohort.

In order to check whether the predictive models differ between Asians and Europeans, we first evaluated accuracies of the predictive models using a cross-validation design (Figure 5A, Methods) and observed higher predictive accuracy in Asians (Figure 5F). Higher accuracy in the predictive model (*i.e.* c-index) observed in the Asian cohort (Figure 5F) raised an interesting question: whether this difference in predictability between cohorts is due to ethnic differences. When we compared predictive accuracies with and without the P2 subtype for the Asian cohort, we observed a significant decrease in the accuracy when excluding P2, indicating that ethnic differences indeed contribute to better predictability in Asians (Figure 5G). Taken together, ethnic differences in HCC not only endow us a better predictive model for patient survival in Asians, but also suggests higher potential for a more effective precision medicine program for HCC in Asia.

## Discussion

With the completion of the TCGA and ICGC projects, the study of ethnic differences has been now becoming one of the central topics in cancer genomics 50,51. By comparing Asian and European cohorts, we presented one of the first systematic comparisons of HCC genomes and identified a suite of genomic events ranging from TMB, mutational signatures as well as CNAs segregating distinctively across two cohorts. Most strikingly, we identified an Asian specific transcriptomic subtype with enriched driver genes (*e.g. AXIN1*), higher genomic instability (in particular chromosome 16 deletion) as well as a much colder immune profile with high levels of MDSC cells. Ethnic differences, especially higher genome instability, seem to drive the evolution of a unique transcriptomic subtype and better patient prognostic prediction in Asians. Interestingly, despite Asian cohort is enriched for HBV positive patients, Asian-enriched P2 subtype had a significant lower proportion of HBV carriers compared to other subtypes (Figure S17A). In previous studies, HBV positive HCC was found to have a better overall survival possibly driven by higher screening rates 52,53 and patients in the P2 subtype might represent those non-viral carriers with advanced diseases. For the first time, we uncovered an unprecedented amount of ethnic differences in HCC and highlighted an interesting example of how genome instability can drive a collection of ethnic differences between the two cohorts.

The integrative analysis and ethnic comparison presented in this study suggested several new insights in patient treatment in HCC. First of all, novel drivers identified through integrative analysis, especially the chromatin remodelling genes (*e.g. ARID1B*), are often late-occurring tumor suppressors. Instead of targeted therapy or immunotherapy, genes in this group may be better targeted using synthetic lethal approaches 54. Secondly, while immune checkpoint inhibitors (ICI) are becoming a popular therapeutic strategy, we found that the P2 subtype is immunologically cold and has very high levels of myeloid derived suppressor cells (Figure 4G). Since higher levels of MDSC often include the release of immunosuppressive cytokines or arginase 55, which is mechanistically different from common ICI targets such as PD-1, PD-L1 or CTLA4, regular ICI might not work so efficiently in patients from the P2 subtype. However, studies showed that targeting MDSCs or combining ICI with MDSC-targeting therapies may result in better response for the P2 subtype 56,57. Finally, the Asian specific P2 subtype with higher genomic instability might be sensitive to DNA damage repair (DDR) response inhibitors which have been proven to be effective in many cancer types with defects in DNA repair pathways 58,59. Thus, the integrative analysis and ethnic comparison might pinpoint new possibilities for novel therapeutic strategies in HCC.

Integrating multiple features across layers, including the ITH metrics, yielded a combined survival model with much better prediction accuracy in Asians than Europeans. This difference seems to be contributed by ethnic difference as most of the statistical power for patient stratification in Asians came from the existence of the P2 subtype (Figure 5G). For the first time, ethnic specific genomic events can contribute to differences in patient prognosis and stratification in HCC. Despite this difference, it is still surprising to see that molecular events provide so poor predictive power in Europeans and is worth investigating in a future study. For the first time, the integrative survival analysis provided a unique opportunity linking ethnic differences with patient stratification and shed light on differential strategies for precision medicine between ethnic backgrounds.

Even though pan-cancer analysis of ethnic differences has recently been explored, due to biased patient collection in different cancer types, earlier ethnic studies have focused on African and European comparison 60. One important conclusion from recent studies is that ethnic differences with large effect sizes are often specific to individual cancer types 61, emphasizing the importance of targeted analysis focusing on specific cancer types. Through an integrative analysis using the TCGA cohort, we revealed a cascade of molecular events starting from genomic changes (*i.e.* genome instability) to molecular phenotypes (*e.g.* an ethnic specific transcriptomic subtype) and subsequently to patient predictive differences. The study presented here not only provides a unique “molecular mechanism” connecting multi-layer ethnic differences, but also constructed a foundation for integrating potential inconsistent discoveries across ethnicities for the field (Supplementary Note 5). Ethnic differences in HCC depicted here provide one of the best examples for understanding ethnic differences across cancer types.

## Materials and Methods

### Patient cohorts for integrative driver identification

Five largest cohorts of hepatocellular carcinoma (HCC) genomes were collected including the TCGA, the ICGC (LICA-FR, LIRI-JP, LINC-JP), as well as a Korean study 5 (Supplementary Table S2). For the ICGC and TCGA cohorts, mutation data were downloaded from ICGC and Firebrowse websites 62. For the Korean dataset, somatic mutation data were collected from the original publication 5. Somatic mutations were first annotated using Oncotator (Version 1.9.2, based on hg19) 63. Since the combined dataset contains both the whole exome and whole genome sequencing datasets, samples were standardized by taking only the coding variants. Hypermutated samples with more than 1000 coding mutations were excluded (n = 11). After identifying driver genes with MutSigCV (version 1.41) 19, 20/20+ 18 and TUSON Explorer 17, a final list of driver genes was curated by combining results from all three methods and genes with less than 1% frequency were further filtered away. In the saturation analysis, different number of down-sampled subsets (n = 100, 250, 500, 750 and 1000, each replicating 5 times) were sampled and MutSigCV was used to identify candidate drivers 64.

### Literature reported driver genes and pathways

We compiled literature reported driver genes from eight large cohort studies 4-7,10,13,15,65 (n = 88) (Supplementary Table S1). Cancer Gene Census (CGC) genes were downloaded from the Catalogue of Somatic Mutations in Cancer (COSMIC) database (GRCh37/COSMIC v83) 66. To compile significantly altered pathways in hepatocellular carcinoma, multiple significantly mutated pathways in HCC from seven genomic studies 5-7,10,15,67,68 were compiled and a consensus list of pathways was curated by grouping pathways with similar functions.

### Significantly altered pathways in driver genes

Using the combined driver list (n = 62), ConsensusPathDB 69 and g:profiler 70 were used to identify significantly altered pathways. g:Profiler applies the Fisher's Exact test to identify over-represented pathways in a given gene list. ConsensusPathDB employs hypergeometric tests to find significantly altered pathways. For both methods, pathways from the Kyoto Encyclopedia of Genes and Genomes (KEGG) 71 and Reactome 72 databases were selected. In addition, pathways from BioCarta 73 database were also included in the ConsensusPathDB analysis. Given the output from these two methods, pathways with similar biological functions were first grouped together and classified into literature known pathways as well as novel ones.

### Mutual exclusivity and co-occurrence analysis of driver genes

Using the presence and absence information across variables, the association between drivers as well as between drivers and clinical phenotypes were tested using the Fisher exact test (co-occurrence and mutual exclusiveness representing the two tails of the test). Multiple test correction was carried out using the Benjamini-Hochberg method and FDR cutoff of 0.1 was used to select significantly perturbed gene pairs.

### Reprocessing the TCGA data for ethnic comparison

WES data from the TCGA cohort was downloaded from GDC 74 and somatic mutations were called using Mutect 75. Among the identified driver genes (n = 62), Fisher's Exact test was applied to test for frequency differences in each gene and Benjamini-Hochberg method was used for multiple test correction. Drivers with q value less than 0.1 were selected as significantly different genes.

### Inferring cancer cell fraction and timing of mutations

Cancer cell fraction (CCF) of single nucleotide variants was calculated similar to McGranahan *et al.* 44,76. In particular, VAF = *purity* × CCF / (CN_normal_ × (1-*purity*) + *purity* × CN_mutation_). CN_normal_ is the copy number of the loci in the normal sample. Copy number 2 (diploid) was used for autosomal mutations. For mutations in the X chromosome, 2 was used for female patients and 1 was used for male patients. CN_mutation_ is the mutation copy number and was calculated by local copy number and tumor purity (values estimated from the Sequenza 77). Mutations with total number of reads less than 10, number of alternative alleles less than 3 and/or allele frequency of less than 0.05 were filtered out. For each mutation, we conducted binomial modeling of observed VAF. A likelihood function is defined by calculating the binomial probability using the depth of coverage as the number of trials. Then, a deviance function was defined as -2*sum(log-likelihoods). Finally, deviance function was optimized between [0,1] interval using optim() function in R to find the CCF value which minimizes the deviance function (*i.e*. the highest binomial probability). Timing of the mutation was classified based on cancer cell fraction (CCF). Early mutations were defined as CCF ≥ 0.8 and late mutations were mutations with CCF < 0.8.

### Mutational signatures and subgrouping

A list of 10 HCC related mutational signatures from previous studies were collected 12,22. DeconstructSigs 24 was used to deconvolute mutations into these signatures from the COSMIC v3.1 78. Signatures with significant contribution (*i.e.* mean proportion greater than 2% or a maximum proportion of 20% across samples) were kept and deconvolution was repeated with the set of significant signatures. With the contribution of different signatures estimated for each patient, signature proportions were clustered using the hierarchical clustering algorithm with the Euclidean distance and 'ward.D' method in R. Comparison of signatures between early and late tumorigenesis was performed using the paired Wilcoxson test. Timing of signatures was conducted by separate deconstruction of early and late mutations.

### Copy number inference and the GISTIC analysis

Sequenza 77 was employed to infer the integer copy number using the raw WES data. Genomic instability index (GII) was calculated by comparing the copy number of each segment with median copy number across the genome of the patient. GII is simply the fraction of the genome with an integer copy number different from the median ploidy. Somatic CNA (SCNA) score was calculated for arm and broad scale CNV output from the GISTIC algorithm using a method similar to Yuan *et al.* (2018) 60. GISTIC 79 was employed to identify significantly perturbed CNVs for the Asian and European patients from the TCGA cohort separately to compare arm level and focal CNV events using parameters as following:“*-genegistic 1 -smallmem 1 -broad 1 -brlen 0.5 -conf 0.95 -armpeel 1 -savegene 1 -gcm extreme”.* Arm level frequencies were compared across cohorts using results from the *broad_significance_results.txt* based on the Fisher's exact test and p-value for each variable was adjusted for multiple testing using the Benjamini-Hochberg method with a cutoff value of 0.1. For focal amplifications, q values from the scores.gistic output were used. Common and private peaks were identified by overlapping peak limits from Asian and European cohorts using the “GenomicRanges” R package. Driver genes, a list of pan-cancer amplifications and deletions 80 and a list of known HCC copy number events 25 were labelled for the peaks.

### Transcriptomic subtypes and ethnic comparison

Raw gene counts were downloaded from the GDC 74. Protein coding genes were used for further analysis and lowly expressed genes were filtered out (*i.e.* removing genes with less than 5 counts in at least ten patients). Gene expression levels were normalized using DESeq2 81 and subsequently log2 transformed after adding 1 pseudo count.

For molecular subtypes, top 3000 most variable genes (based on median absolute deviation, MAD) were selected for both Asian and European cohorts separately. Non-negative matrix factorization (NMF) algorithm was applied in the NMF R package with the Brunet algorithm 82. Number of ranks from 2 to 6 were iteratively run for 200 times. Optimal rank (number of subtypes) was selected based on the highest cophenetic correlation and the highest consensus silhouette values.

Mapping of homologous subtypes was conducted using the SubMap from the Genepattern with default parameters 37. Differentially expressed genes were identified using DESeq2 81. Gene set enrichment analysis was conducted using the “fgsea” method 83. Hallmark (v6) and C2_CGP (v7) gene sets were used for fgsea and genes were ranked according to a combined significance score (sign of log fold change times -log10 of p-value) and significant pathways were extracted (fdr < 0.05).

In order to measure the enrichment of a pathway in a group of patients, gene set variation analysis (GSVA) was conducted to calculate a pathway level score^84^ (Figure 3c, 3d). Comparisons of clinical and genomic differences were conducted using the Wilcoxon test for continuous variables and Fisher's Exact test was used for categorical variables. P-values were adjusted using the Benjamini-Hochberg method (< 0.1).

Due to limited samples size, we assigned patients from external Chinese cohorts to the transcriptomic subtypes. Top 100 up-regulated genes of each subtype (compared to the rest of the subtypes) were used as signature (template) genes. Nearest template algorithm was applied using “CMScaller” R package 85. Predicted classes (subtypes) were selected based on the false discovery rate (fdr < 0.1) otherwise patients were assigned to the “NS” group.

### Annotating literature known transcriptomic subtypes

To understand the concordance of identified transcriptomic subtypes with transcriptomic subtypes identified by earlier studies 26-29,31,32,37, gene signatures of previous subtypes were downloaded from MSigDB 86. Pathway activity score was calculated similar to a previous study 87. Patients were assigned to the subtype with the highest pathway activity score. For previous studies without assigning to discrete categories (*e.g.* single gene signature such as the EpCAM signature in Yamashita *et al.* 2008), pathway activity score was plotted as a continuous value indicating the expression level of the gene.

### Statistics for measuring intra-tumor heterogeneity (ITH)

Percentage of late mutations (pLM) was calculated as the proportion of late mutations (CCF < 0.8). MATH score was calculated as described in the original study 49. Pyclone 88 was employed to infer the clonal structure of the tumor. Binomial density and 10,000 iterations were selected for the MCMC inference (the first 1000 iterations were treated as the burning phase). Shannon index was calculated using the number of mutations in each subclone identified in pyclone as 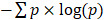, where *p* is the mean CCF of each cluster.

### Integrative survival analysis

A total of 44 features from clinical (n = 7), molecular (n = 22), driver genes (n = 12) and ITH (n = 3) categories were first compiled. Clinical features include gender, age, stage, HBV and HCV status, grade and race (only for the combined TCGA cohort). Molecular features included: 1) basic tumor features such as purity, ploidy, transcriptomic subtypes, TMB, SCNA; 2) proportions for common mutational signatures (*i.e.* with a mean proportion of no less than 5% across patients which include SBS4, SBS5, SBS12, SBS22); 3) immune features including immune subtypes, MDSC score, GEP 89; 4) frequent copy number events with a cohort frequency of at least 40% in either Asian and European cohort. Driver features included genes with at least 15 mutations across all patients. Finally, ITH variables included pLM, MATH score and Shannon's index. Random forest survival (RFS) algorithm was implemented to calculate the concordance index (c-index) distributions and feature importance 90. Random forest feature selection was applied 50 times with a random subset (75%) of cohorts separately (Asian, European and combined TCGA) and features which were selected at least 25 times across runs were selected for each cohort. The union list of all selected features across cohorts was used as the final feature list. Hyper parameter tuning is done by 100 times random search for random forest (RF) algorithm parameters such as number of trees (1500), node size (10), number of selected features for each tree (3), and number of splits (25) and optimal parameters were chosen with 5-fold cross validation. Distribution of accuracies were obtained by splitting cohorts 50 times (75% training set, 25% test set) and running the tuned RF algorithm across different cohorts (*e.g.* Asian, European and combined TCGA) and different feature categories (*e.g.* clinical, molecular, driver, ITH or all features). Concordance index (c-index) was used to evaluate the model accuracy in the test set (25%). Feature importance was calculated using RF variable importance method (VIMP) 91 on the full models for each cohort (50 times) and the average rank of each feature across runs was used as the importance score for each feature.

### Data and code availability

Somatic mutation data for LIRI-JP, LINC-JP and LICA-FR cohorts were obtained from the ICGC database (https://dcc.icgc.org/releases/release_25/Projects/). Raw data (BAM files) for Korean cohort was obtained from the authors. TCGA-LIHC WES and RNAseq data are obtained from GDC (https://portal.gdc.cancer.gov/). Custom R code for the analyses implemented in this work can be obtained upon request from the authors.

## Supplementary Material

Supplementary figures, notes, and tables.
