# Supplementary material for: Genome instability is associated with ethnic differences between Asians and Europeans in hepatocellular carcinoma
Source: Theranostics. 2022 Jun 6;12(10):4703–17. doi: 10.7150/thno.71676 (PMC9254249; doi:10.7150/thno.71676)
Supplement: Supplementary file 1 — Supplementary figures, notes, and tables. [file thnov12p4703s1.pdf]

Supplementary Figures

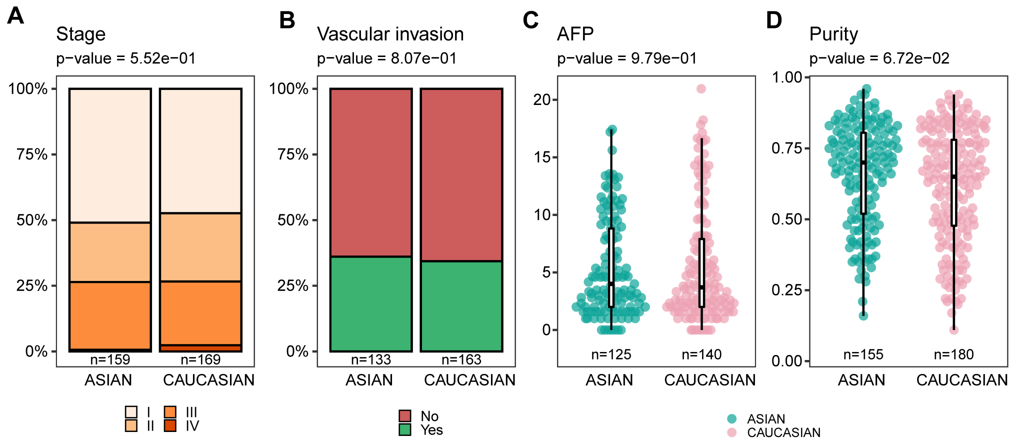

Figure S1: Comparison between the Asian and European cohort in the TCGA dataset. Ethnic comparison in A) Stage, B) Vascular invasion, C) AFP as well as D) purity between Asians and Europeans in the TCGA cohort.

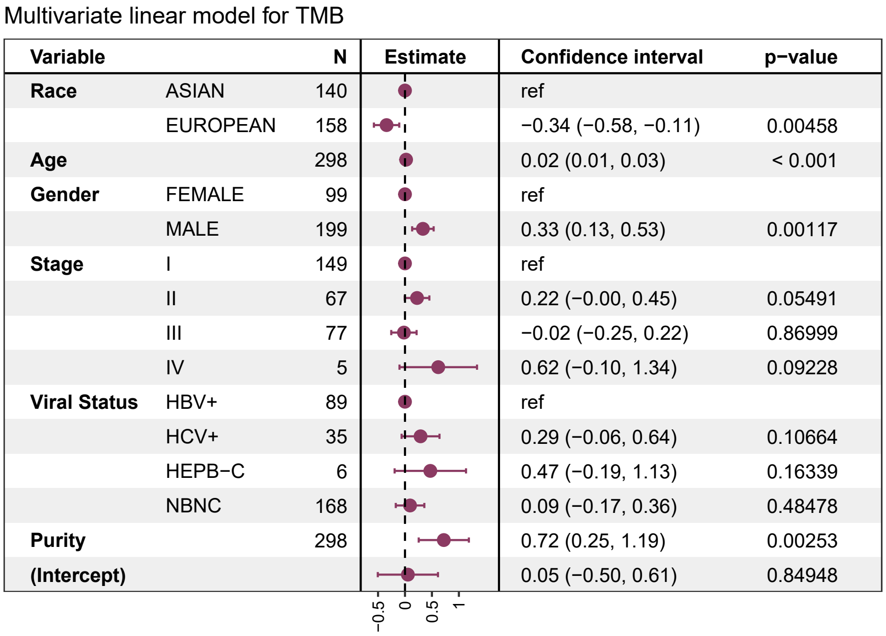

Figure S2: Multivariate linear model comparing TMB across cohorts. Multivariate analysis between TMB and the ethnic background controlling for a collection of clinical variables. TMB difference in ethnicity was still significant after controlling for tumor purity and other clinical variables.

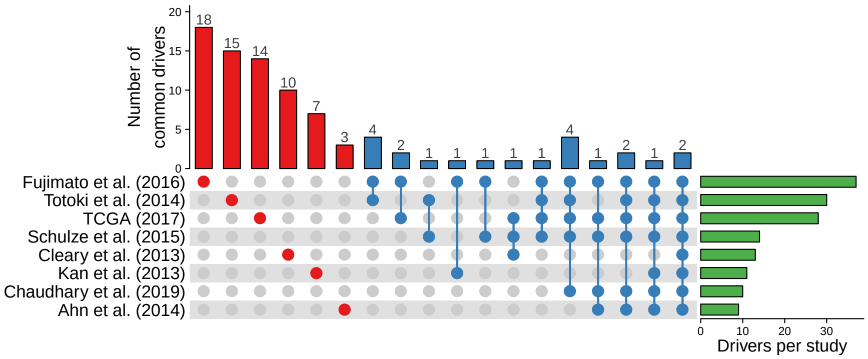

Figure S3: Driver genes identified in a few large cohorts. Summary of driver genes across cohorts. Right bars indicate the number of driver genes identified in each study are summarized. Top bars indicate the number of genes shared by all possible combination of studies. Blue bars denote the number of genes identified by at least two studies and red bars indicate the number of genes identified by only one of the studies.

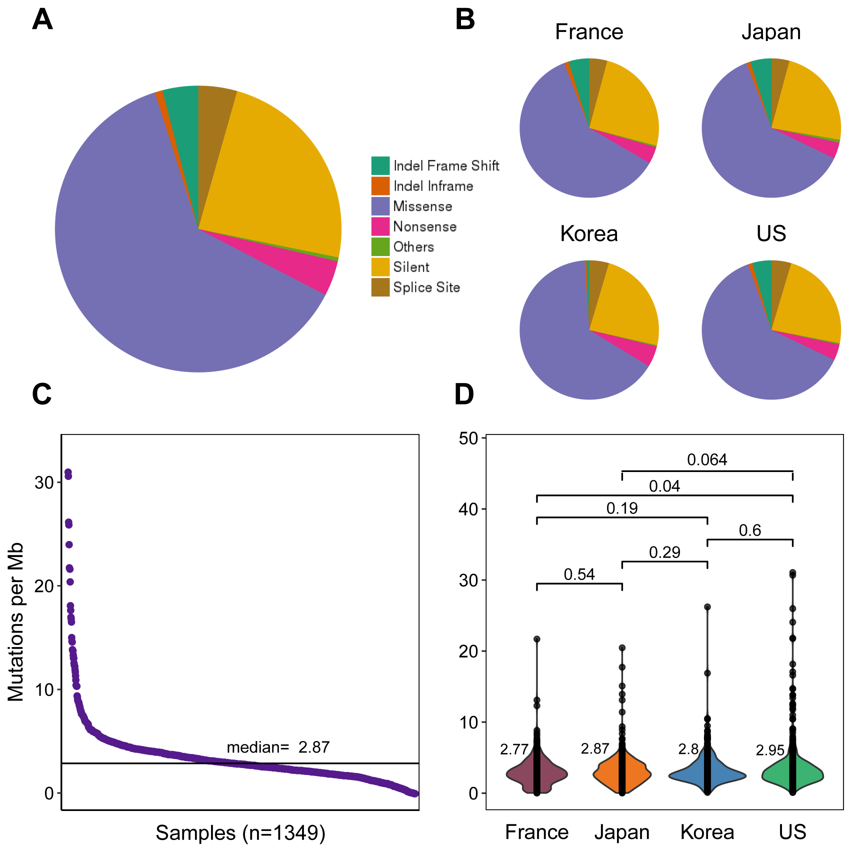

Figure S4: Summary of mutation profile across cohorts.A) Distribution of mutation types across all 1349 patients, B) Distribution of mutation types in different individuals, C) Distribution of mutation rates (i.e. number of mutations per megabase) across all 1349 patients, D) Mutation rate distributions in different cohorts. Median mutation rates are shown for each cohort. P-values from the Wilcoxon signed-rank test across cohorts were listed for each comparison.

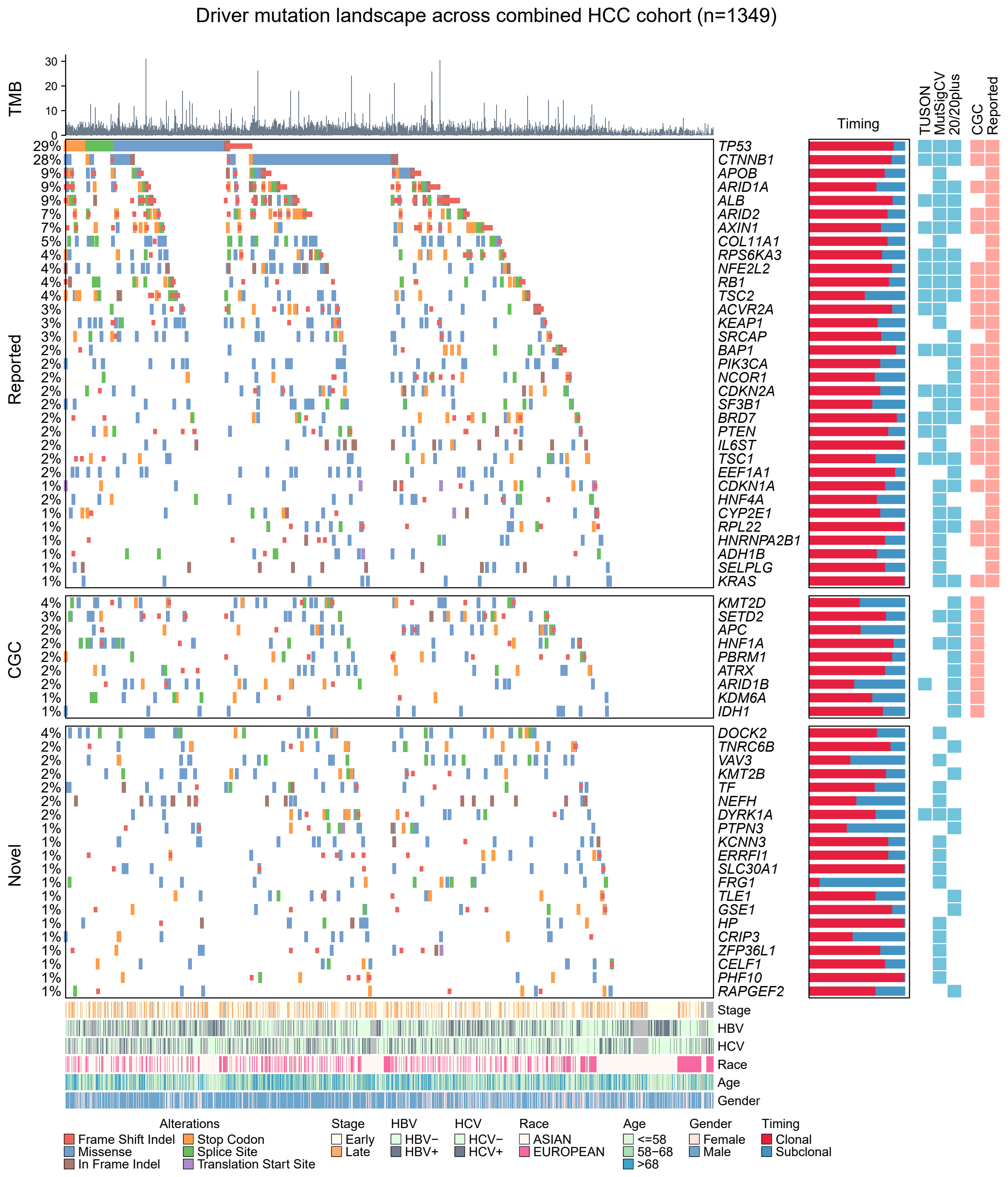

Figure S5: The landscape of 62 HCC drivers across 1349 patients. Each column represents a patient. In each row, somatic alterations (represented by different colors) and mutation frequencies for each driver were given. The timing barplot on the right side indicates proportions of early (red) and late (blue) mutation proportions in the driver across the cohort. Heatmap on the right side indicates whether the driver gene is detected by different methods (MutSigCV, TUSON Explorer and 20/20+) or whether the gene was reported previously by other studies or in the cancer gene census list (Reported or CGC). The barplot on the top indicates the mutation burden of patients. Clinical phenotypes of patients were shown at the bottom of the figure.

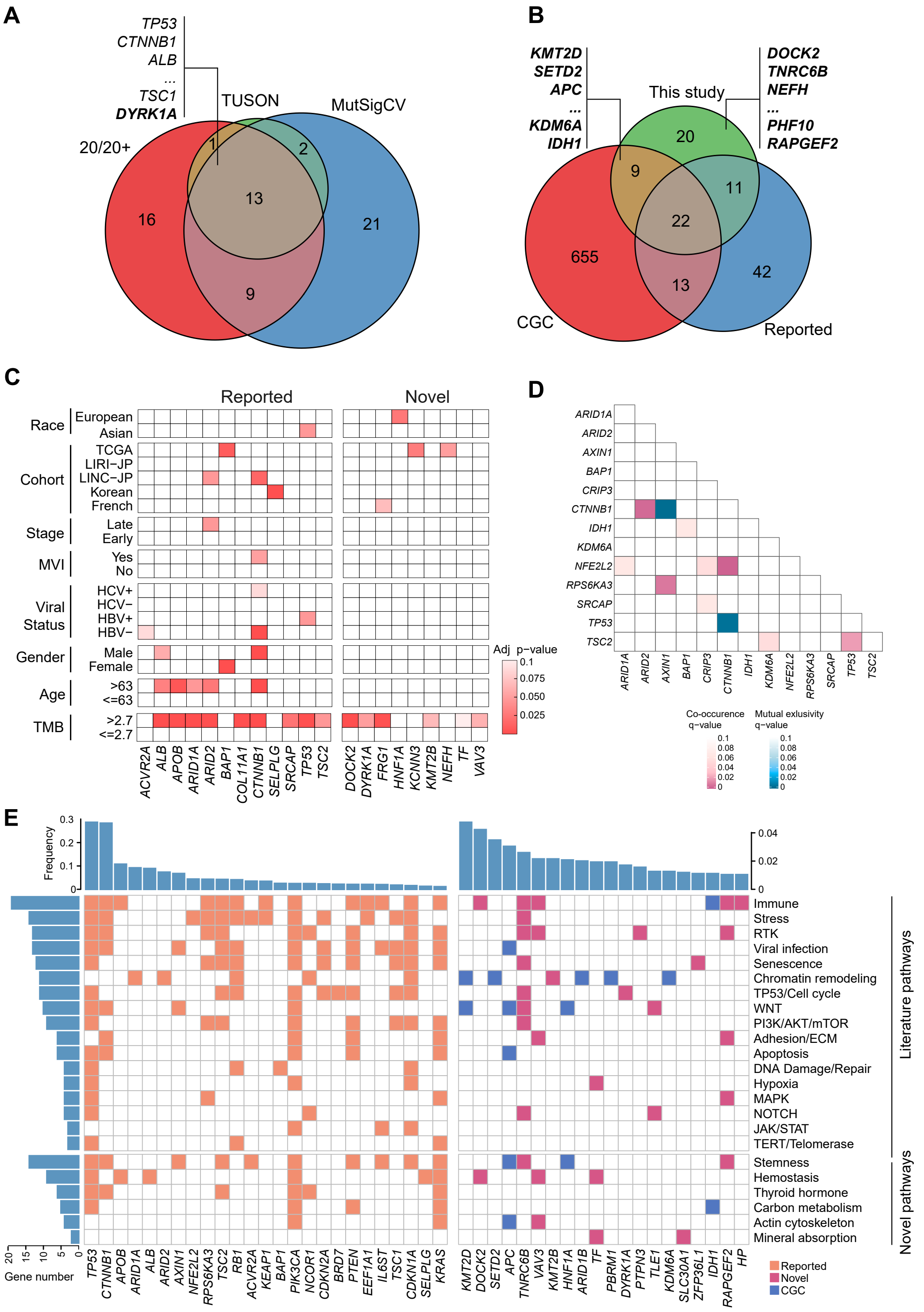

Figure S6: Driver genes identified in different methods. A) Venn diagram of drivers identified by three different methods, B) Venn diagram of overlapping and unique drivers identified in this work, previous studies (Reported) and the Cancer Gene Census (CGC) database. Novel genes were shown in bold font, C) Correlation of drivers with clinical phenotypes. The color scale represents the adjusted p-value after Benjamini-Hochberg multiple test correction, D) Mutual exclusivity and co-occurrence status of drivers. Pairs with a q-value < 0.1 were kept. The q-value for each significant mutually exclusive or co-occurrent pair are shown as gradients of pink (co-occurring) and blue (mutually exclusive),E) Driver genes and their associated pathways . Both literature and novel pathways are shown. The upper panel bar chart shows the mutational frequency of the corresponding driver genes. The left panel bar chart shows the number of genes assigned to corresponding pathways. Heatmaps on the left show pathways for previously reported driver genes and heatmaps on the right show pathways for novel drivers. CGC genes among novel drivers are shown in blue color.

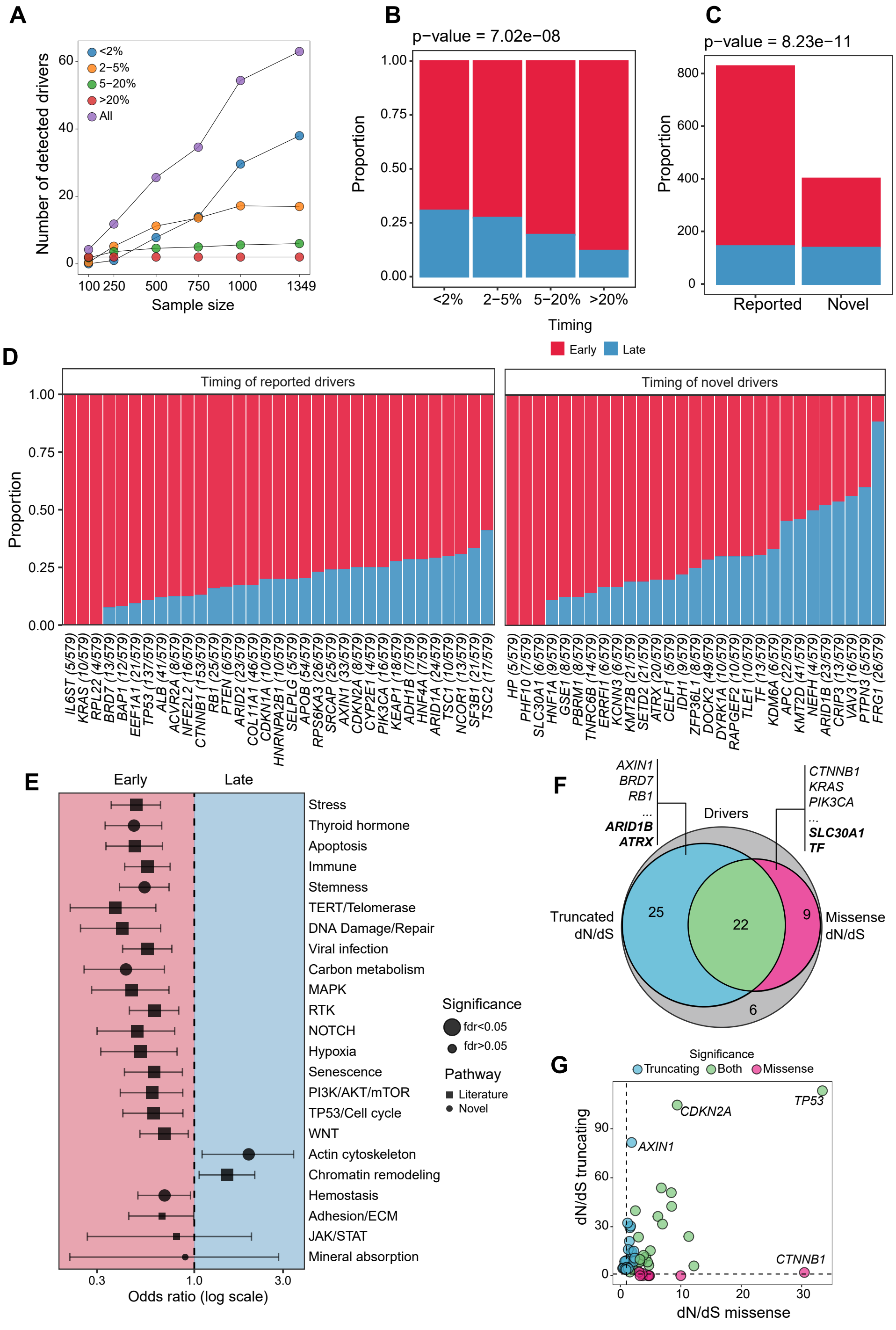

Figure S7: Saturation analysis and the timing of drivers. A) Number of drivers detected with different sample sizes. The relationship between the number of identified driver genes and the sample size (Purple dots). Lines are connecting the median number of detected drivers for that subsample size (5 replicates per sample size). Drivers in different frequency categories across samples are shown with different colors. B) Timing of mutations in driver genes in different frequency groups, C) Subclonal (blue) and clonal (red) fractions of mutations in reported and novel drivers across all patients in the TCGA and Korean cohorts, D)Subclonal (blue) and clonal (red) fractions of mutations in individual drivers across patients in the TCGA and Korean cohorts, E) boxplot for timing of driver genes in different pathways, F)Venn diagram of drivers with significant truncating or missense dN/dS value, G) Truncating versus missense dN/dS values for all the driver genes.

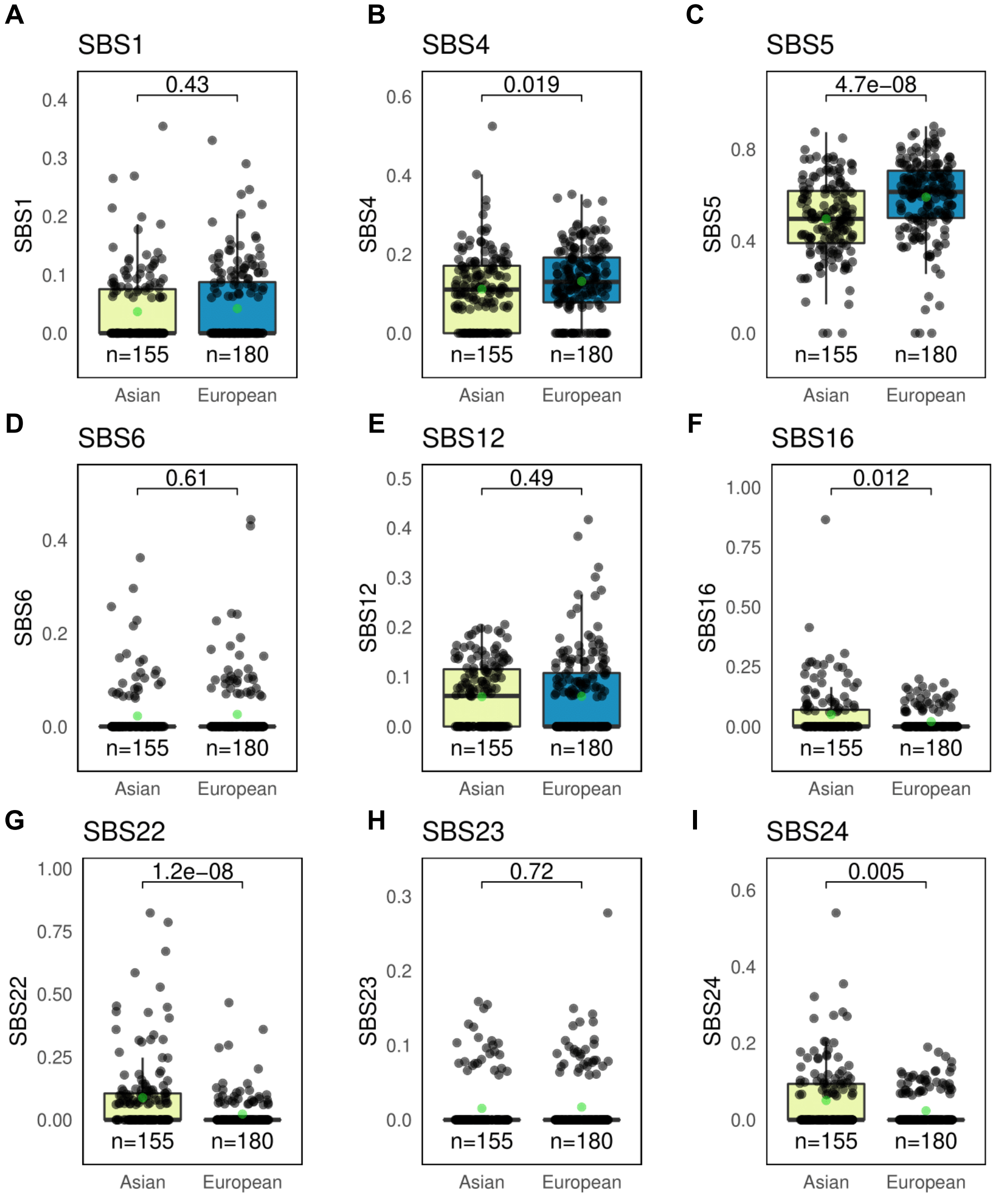

Figure S8: Comparison of mutational signature proportions between Asian and Europeans. A) SBS1 (aging), B) SBS4 (smoking), C) SBS5 (aging), D) SBS6 (MSI), E) SBS12 (liver related signature), F) SBS16 (liver related signature), G) SBS22 (aristolochic acid), H) SBS23 (unknown etiology), I) SBS24 (aflatoxin B1)

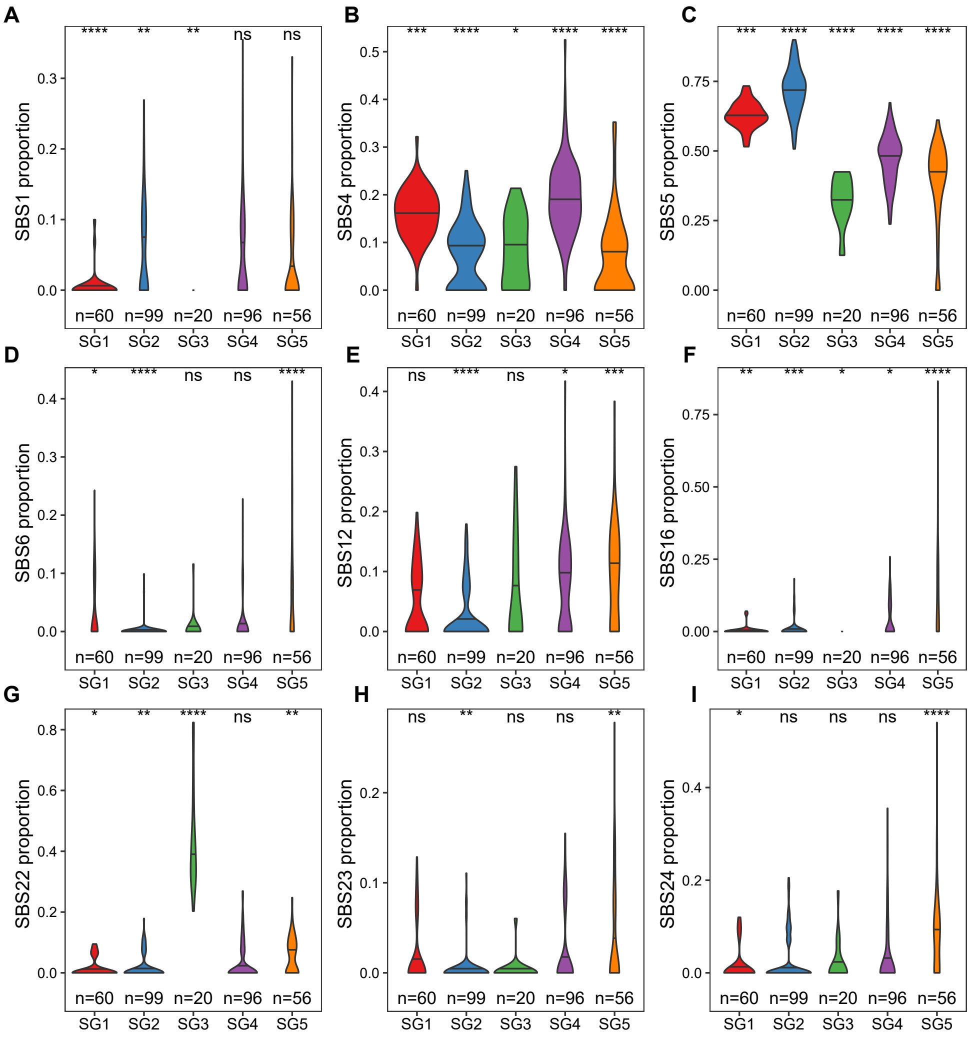

Figure S9: Comparison of mutational signature proportion across signature groups for A) SBS1 (aging), B) SBS4 (smoking), C) SBS5 (aging), D) SBS6 (MSI), E) SBS12 (liver related signature), F) SBS16 (liver related signature), G) SBS22 (aristolochic acid), H) SBS23 (unknown etiology), I) SBS24 (aflatoxin B1). In these comparisons, median value of the signature group was taken as the reference and stars indicates that the signature is significantly different from the median signature proportion across the cohort.

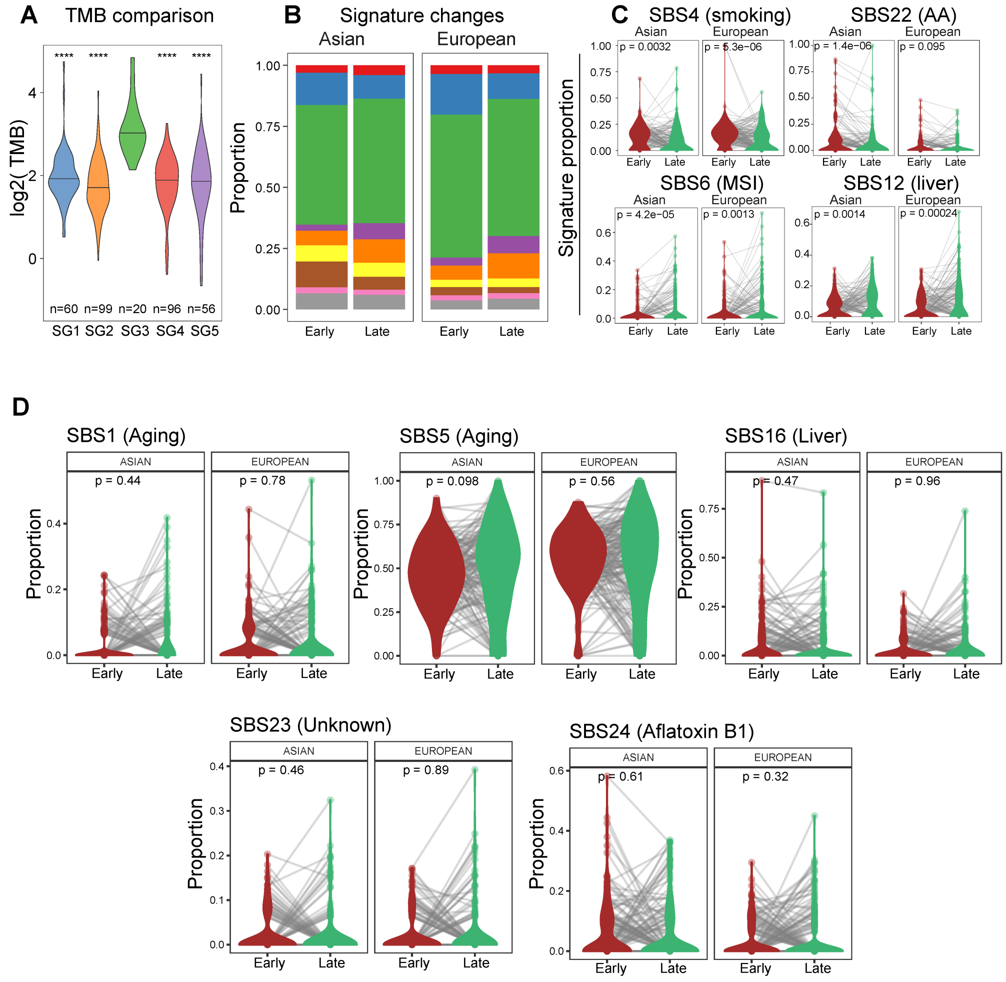

Figure S10: Summary of the signature subgroups. A) Comparison of TMB across signature groups. SG3 is the reference group in the Wilcoxon test and p-value <= 0.001 were labelled as "****", B) Proportions of mutational signatures in early (CCF>0.8) and late (CCF<0.8) mutations in the Asian (left) and European (right) cohorts, C) Violin plots for signatures with significant differences in their proportions between early and late mutations, D)Signatures with similar timing between the Asian and European cohorts.

Multivariate linear model for arm SCNA

Variable		N	Estimate	Confidence interval	p−value
**Race**	ASIAN	140		ref	
	EUROPEAN	155		−0.10 (−0.18, −0.02)	0.0203
**Age**		295		0.00 (−0.00, 0.00)	0.1510
**Gender**	FEMALE	99		ref	
	MALE	196		−0.03 (−0.10, 0.04)	0.3577
**Stage**	I	148		ref	
	II	65		0.08 (−0.00, 0.16)	0.0565
	III	77		0.07 (−0.01, 0.15)	0.0866
	IV	5		0.22 (−0.03, 0.46)	0.0874
**Viral Status**	HBV+	89		ref	
	HCV+	35		0.00 (−0.12, 0.12)	0.9975
	HEPB−C	6		−0.03 (−0.26, 0.19)	0.7632
	NBNC	165		0.01 (−0.08, 0.10)	0.8687
**Purity**		295		0.28 (0.12, 0.45)	<0.001
TP53	wt	211		ref	
	mut	84		0.17 (0.10, 0.24)	<0.001

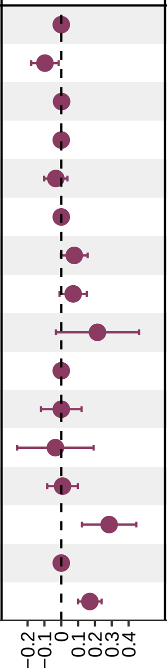

Figure S11: Multivariate linear model comparing arm level SCNA between Asians and Europeans. Multivariate analysis between arm level SCNA and the ethnic background controlling for a collection of clinical variables and driver genes.

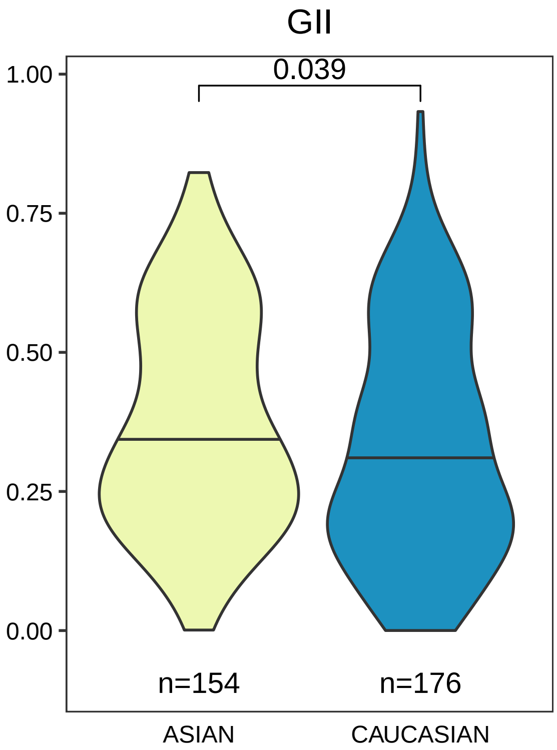

Figure S12: Genomic instability index (GII) differences between Asians and Europeans. Violin plots for GII values between the two cohorts

Multivariate linear model for GII

Variable		N	Estimate	Confidence interval	p−value
**Race**	ASIAN	140		ref	
	EUROPEAN	155		−0.05 (−0.11, 0.01)	0.1121
**Age**		295		0.00 (−0.00, 0.00)	0.5589
**Gender**	FEMALE	99		ref	
	MALE	196		−0.02 (−0.07, 0.03)	0.4526
**Stage**	I	148		ref	
	II	65		0.06 (0.01, 0.12)	0.0316
	III	77		0.05 (−0.01, 0.11)	0.0837
	IV	5		0.23 (0.05, 0.41)	0.0125
**Viral status**	HBV+	89		ref	
	HCV+	35		−0.01 (−0.09, 0.08)	0.8752
	HEPB−C	6		−0.01 (−0.17, 0.16)	0.9198
	NBNC	165		0.04 (−0.03, 0.10)	0.2773
**purity**		295		−0.09 (−0.21, 0.03)	0.1234
TP53	wt	211		ref	
	mut	84		0.11 (0.06, 0.16)	<0.001

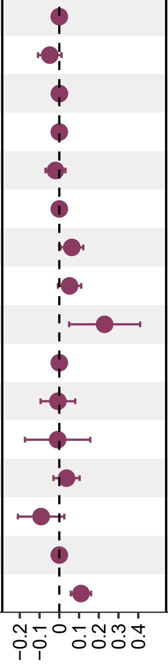

Figure S13: Multivariate linear model comparing GII between Asians and Europeans. Regression analysis between GII and the ethnic background controlling for a collection of clinical variables and driver genes.

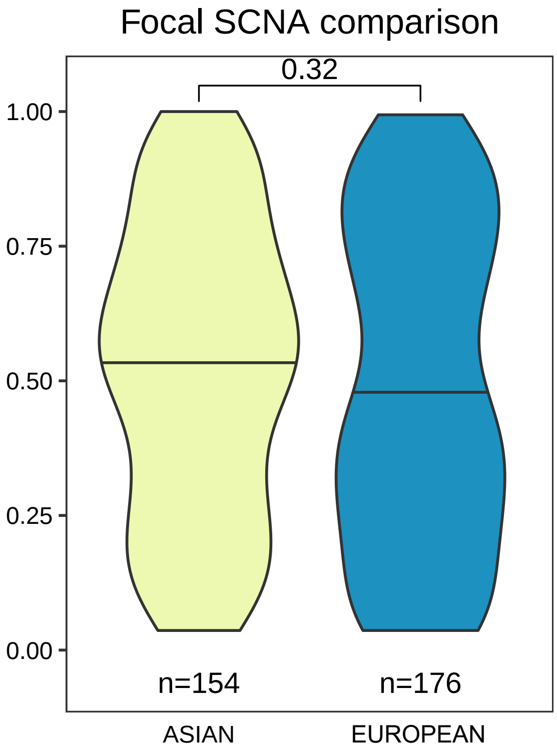

Figure S14: Focal SCNA comparison between Asian and European cohorts. Violin plots for focal SCNA values between the two cohorts

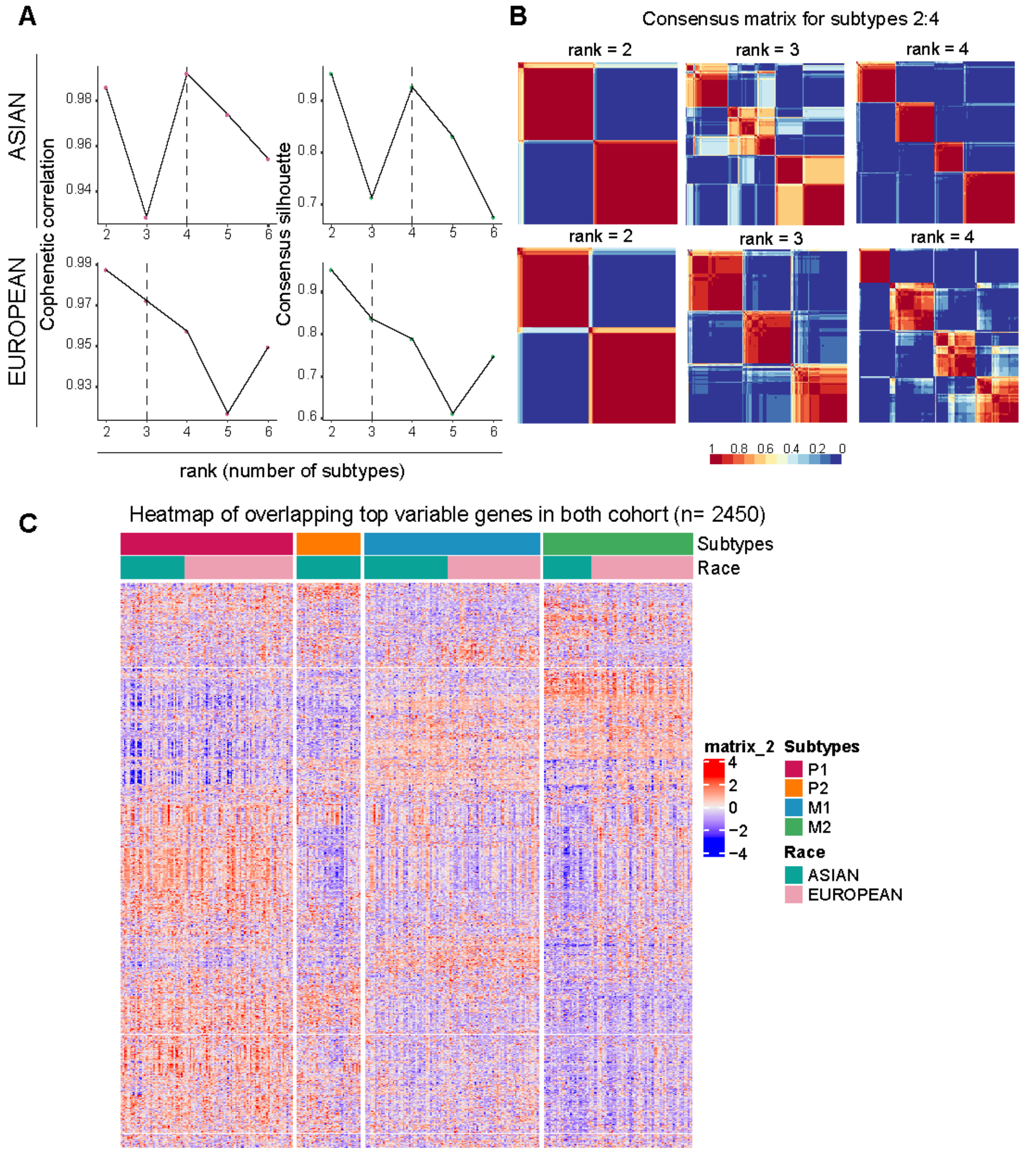

Figure S15: Clustering stability analysis for the two cohorts. A) Cophenetic correlation and silhouette values across the ranks (number of clusters, n=2-4) for Asians (top) and Europeans (bottom), B) Consensus matrix that that shows robustness of subtypes. When patients were assigned to the same subtype across multiple runs of NMF (n=200), they were plotted with a red color (coefficient =1). Top panels are for the Asian cohort and the bottom panels are for the European cohort. C) Heatmap for normalized expression of top most variable genes across identified subtypes. Overlapping genes between Asian and European top 3000 most variable genes are shown (n=2450).

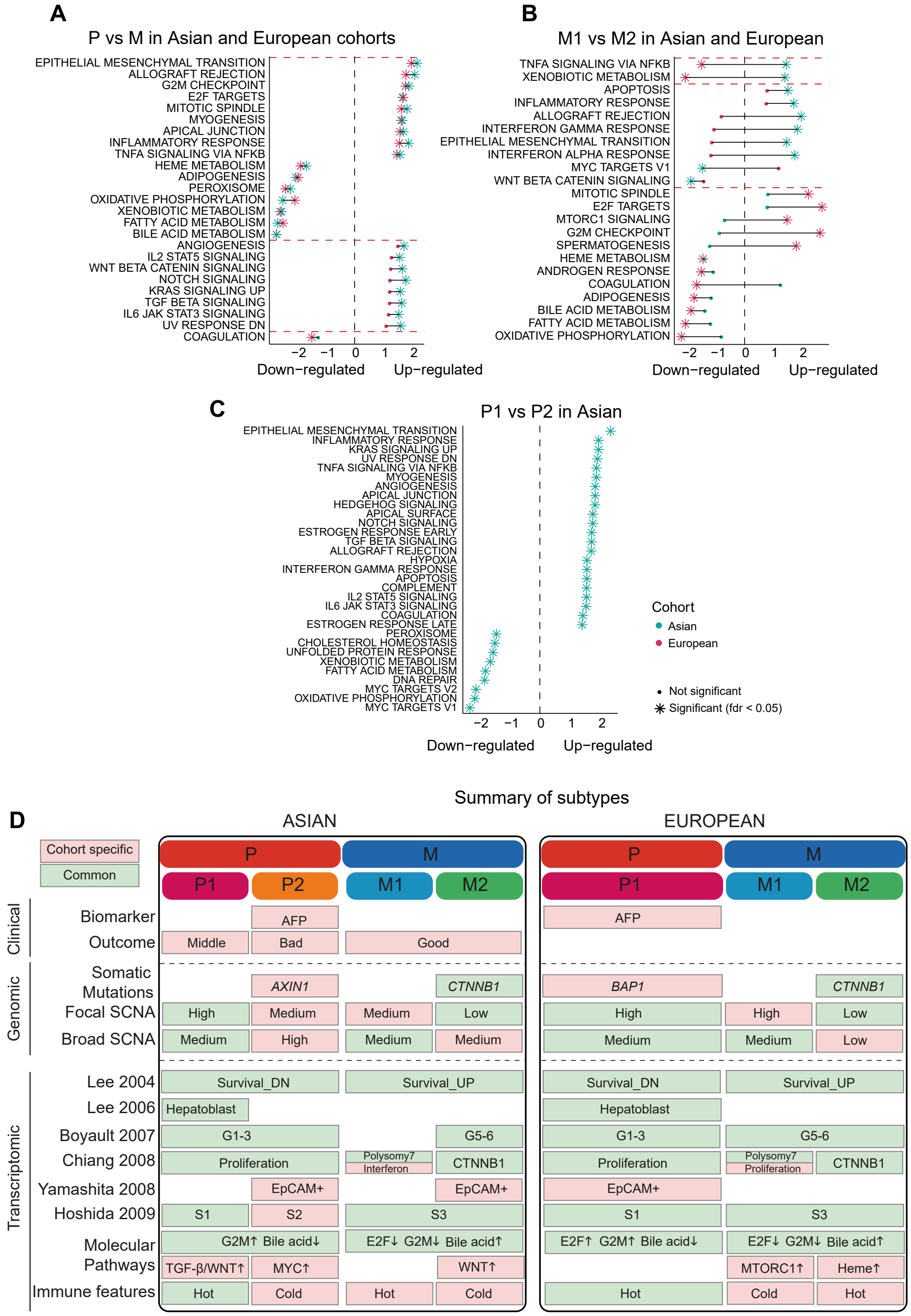

Figure S16: Pathway enrichment analysis for different subtypes in the Asian and European cohorts. A) Comparison between P and M in both Asian and European cohorts, significant pathways were marked with a *, green color represents Asians and pink color is used for Europeans, B) Comparison between M1 and M2 in both Asian and European cohorts, significant pathways were marked with a *, green color represents Asians and pink color is Europeans, C) Comparison between P1 and P2 in the Asian cohort, D) Summary of molecular subtypes and comparison of subtype features. Similar features between two cohorts are shown with a green color while cohort specific features are shown in pink blocks. Corresponding transcriptomic subtypes from the literature are shown in the "transcriptomic" section.

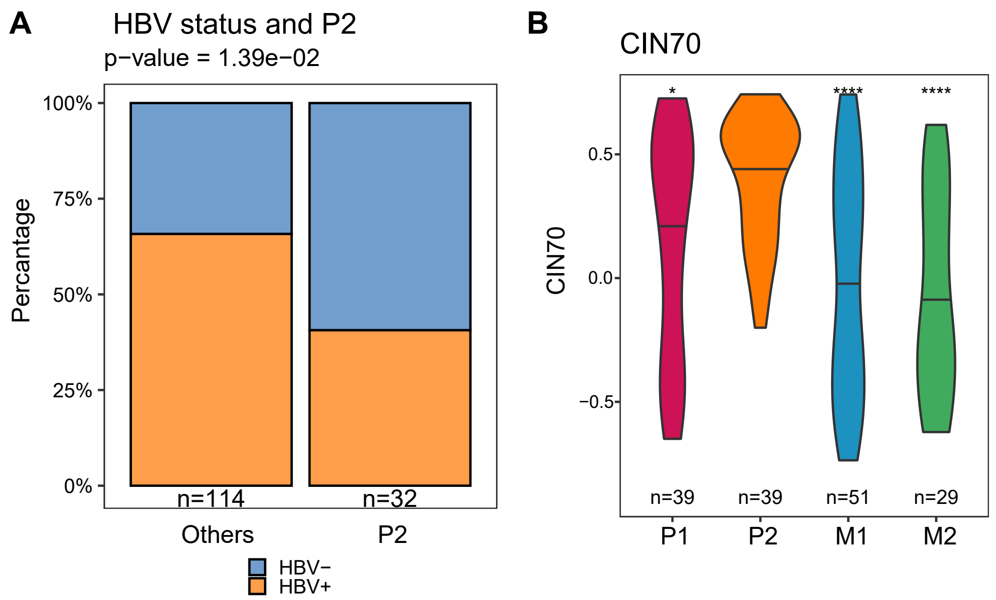

Figure S17: A) Comparison of HBV status between P2 and the rest of the subtypes. B) CIN70 signature gene expression across RNA subtypes. Vioin plot for the CIN70 score across the subtypes. P2 was used as the reference group in the Wilcoxon's rank sum test. * and **** represent p-value < 0.05 and 0.0001.

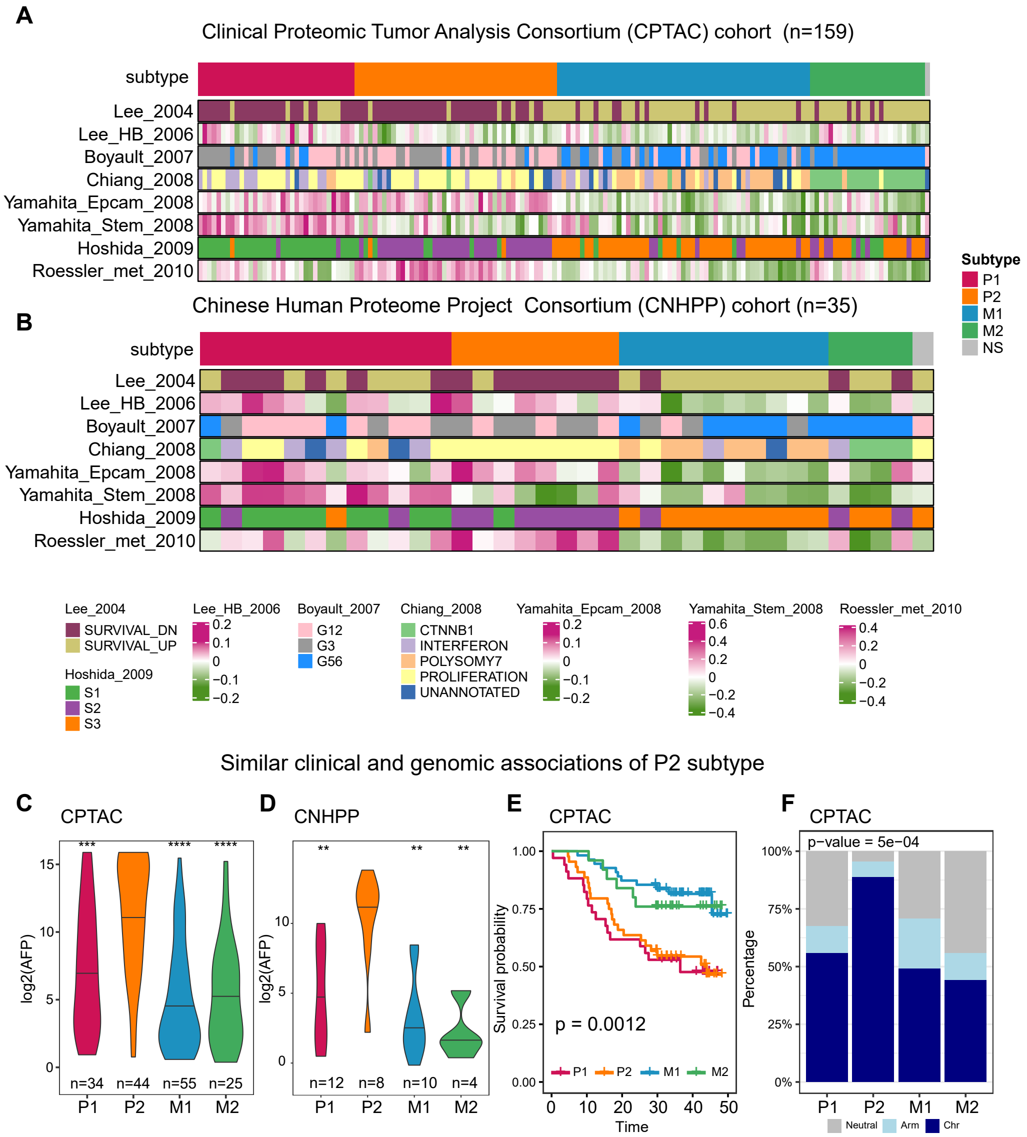

Figure S18: Assignment of patients from the external Chinese cohorts Clinical Proteomic Tumor Analysis Consortium (CPTAC) and the Chinese Human Proteome Project Consortium (CNHPP) to the Asian subtypes (P1, P2, M1 and M2). Similarity of assigned subtypes to literature subtype for A) CPTAC cohort and B) CNHPP cohort. Comparison of AFP levels between subtypes for C) CPTAC cohort and D) CNHPP cohort. P2 was used as the reference group in the Wilcoxon's rank sum test. * and **** represent p-value < 0.05 and 0.0001. E) Survival comparison between subtypes in the CPTAC cohort. F) Comparison of chromosome 16 deletion frequencies across assigned subtypes in CPTAC cohort.

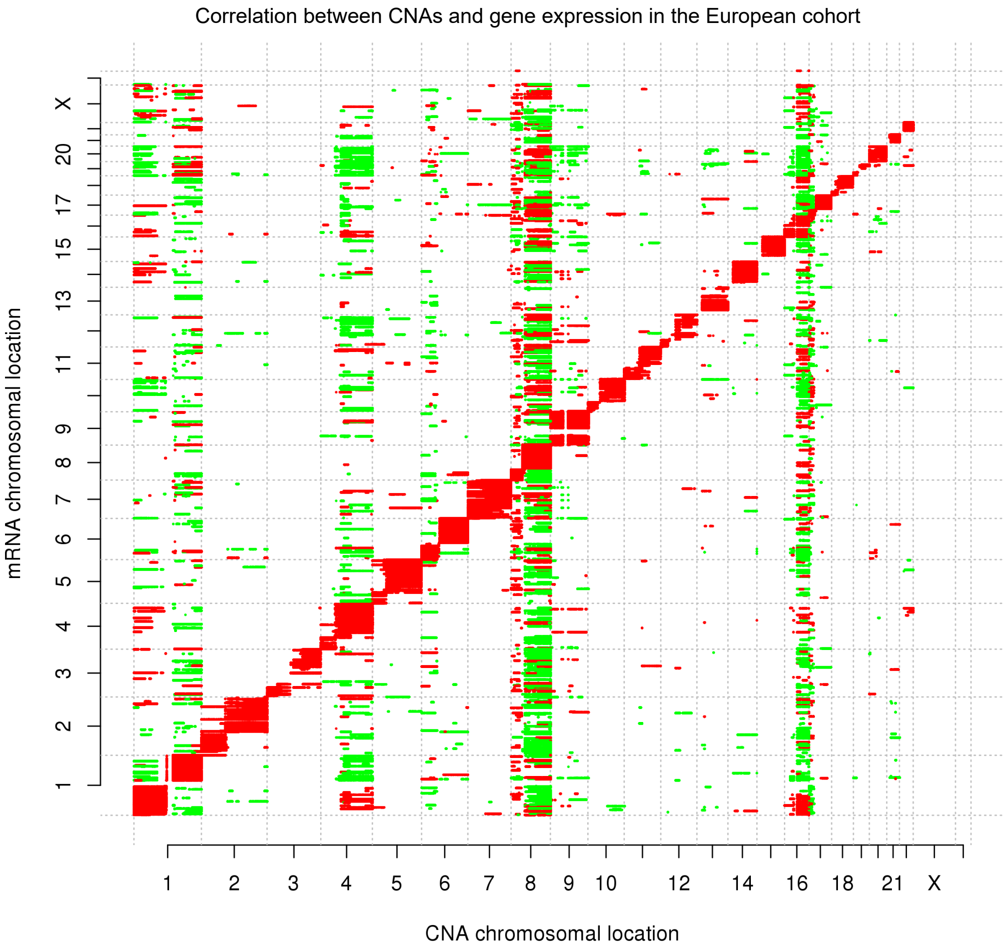

Figure S19: Correlation between copy number alterations (x axis) and mRNA expression (y axis) across the genome in the European cohort. Red color represents a significant positive correlation and green color indicates a significant negative correlation.

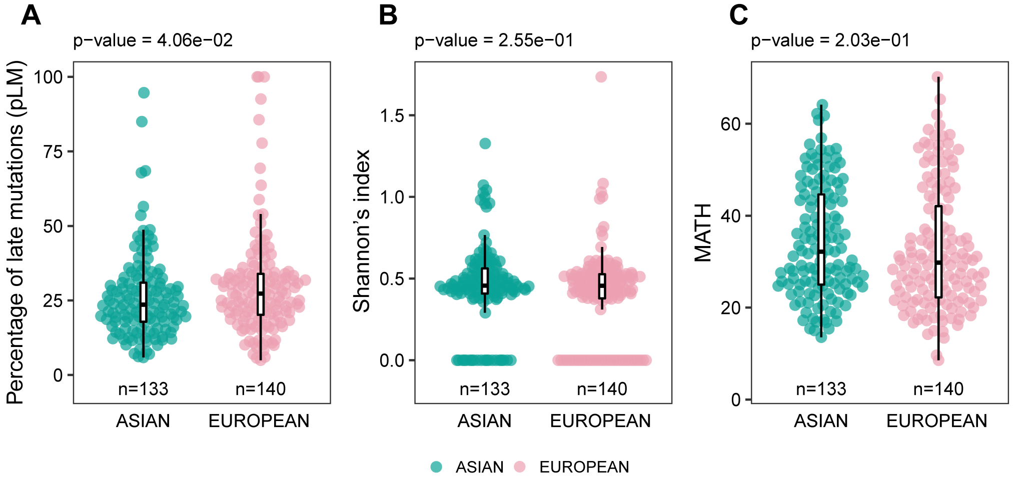

Figure S20: Comparison of multiple ITH features between Asian and European cohorts. A) Violin plot for the percentage of late mutations (pLM) between Asian and European cohorts. B) Violin plot for the Shannon's index between Asian and European cohorts. C) Violin plot for the MATH score between Asian and European cohorts.

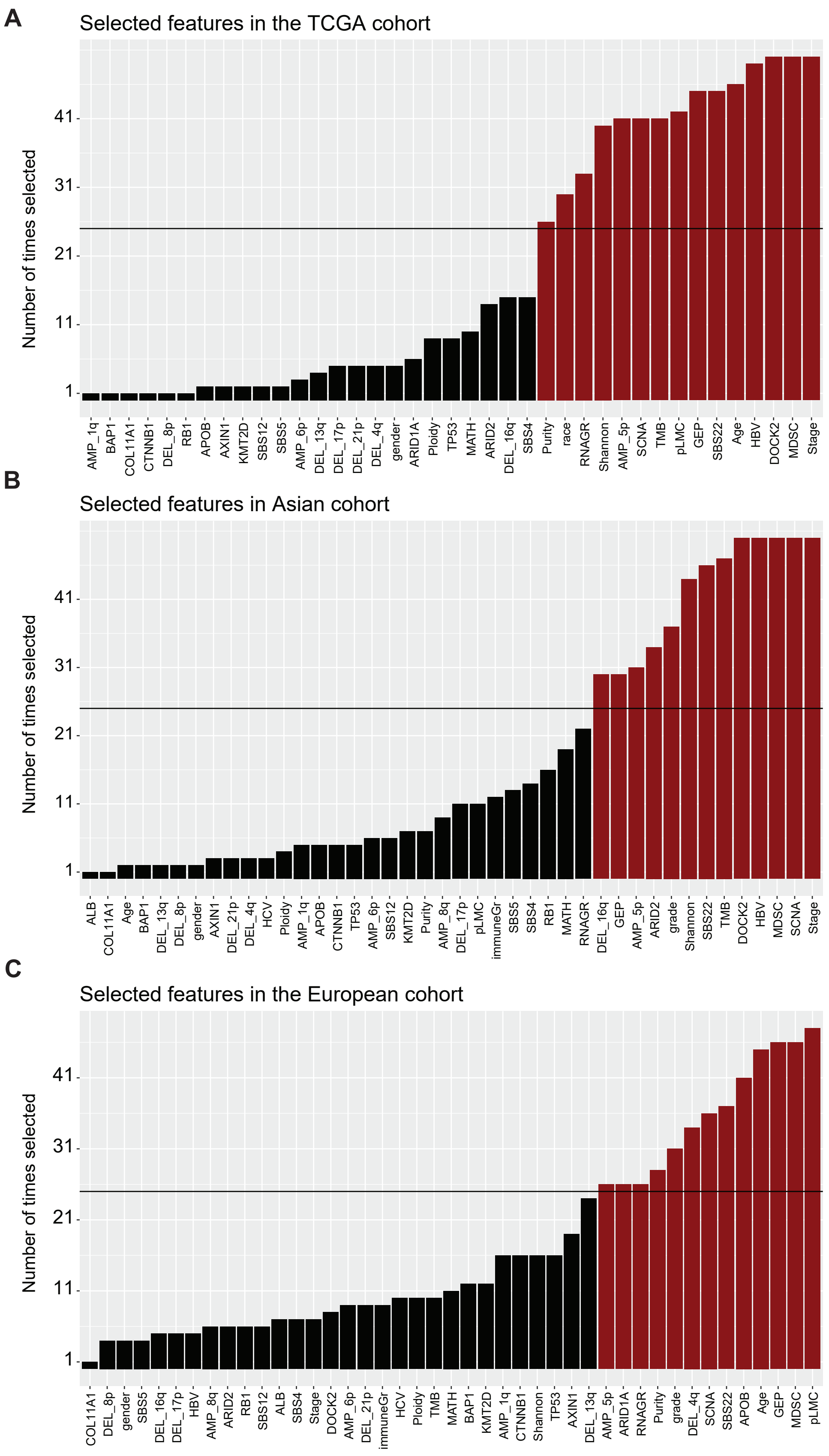

Figure S21: Importance of selected features from the random forest model. Ranking of different features in the A) TCGA, B) the Asian Cohort, C) the European cohorts. Red color represents selected features which have been selected in least 25 times across all runs (n=50)

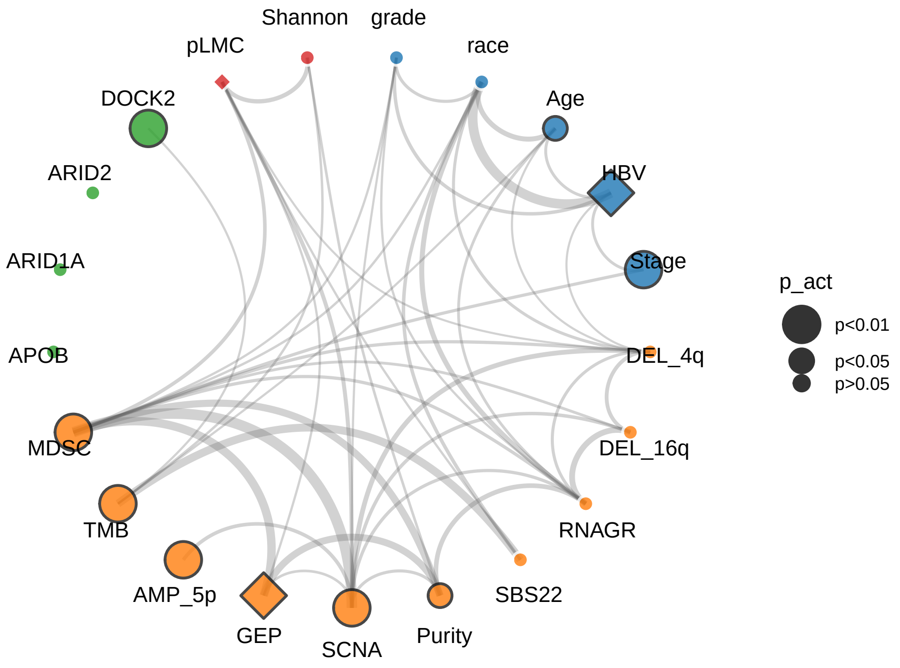

Figure S22: Correlation networkfor the variables that can stratify patients in the combined TCGA cohort. Edges of the network indicate the significance of correlation between features with the width of edges proportional to the re-scaled p-values (-log10(p)). Diamonds represent hazard ratios (HR) less than 1 (good prognosis) and circles represent HRs greater than 1 (poor prognosis). For features with multiple levels such as stage, HR of the most significant level was chosen. The black border around the nodes and size indicates its significance of the variable in the univariate Cox model.

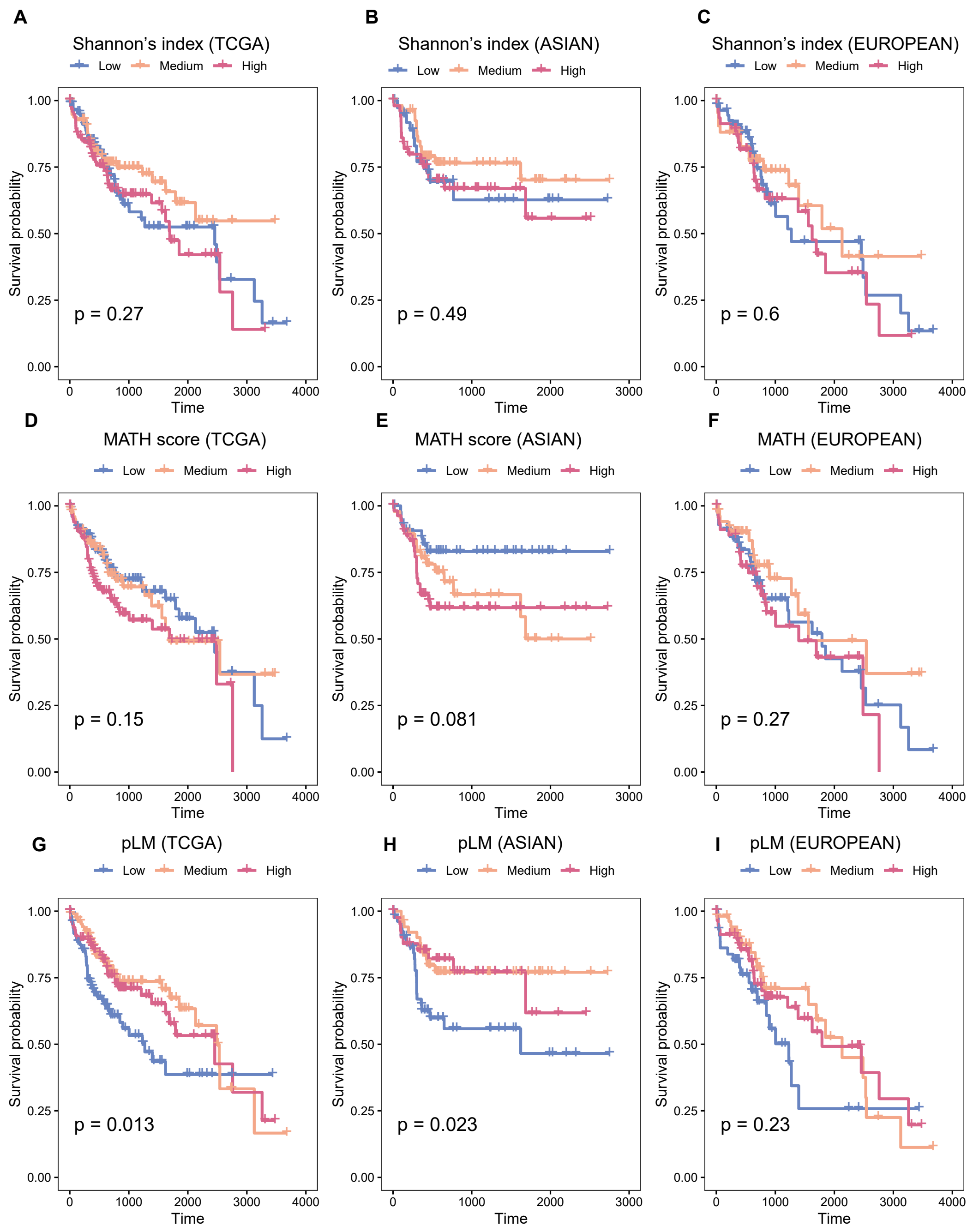

Figure S23: Survival analysis using ITH metrics in the TCGA cohort. Survival curves using the Shannon's index in A) the combined TCGA cohort, B) the Asian cohort, C) the European cohort, Survival curves for MATH score in D) the combined TCGA cohort, E) the Asian cohort, F) the European cohort. Survival curves for percentage of late mutations (pLM) in G) the combined TCGA cohort, H) the Asian cohort, I) the European cohort.

Supplementary Notes

Supplementary Note 1: Integrative driver identification and pathway enrichment

After quality control including removing hyper-mutated samples (greater than 1000 coding mutations, n = 11), the final dataset comprised of 1,349 HCC patients. In the combined driver identification cohort (n = 1,349), 31% of the patients are Europeans (French and TCGA European cohort, n = 179), most of the patients are Asians. Of the disease etiology, 362 (27%) patients are HBV positive and 273 (20%) patients are HCV positive, 13 patients are HBV+HCV infected (1%) and 430 patients are non-viral (non B or C) (32%) (Table S3). Among 129,292 single nucleotide variants (SNV) and 7,242 indels, 62.5% of the SNVs are missense and 23.8% are silent mutations (Figure S4A). While the mutation burden is highly variable across patients, the mutation type and rate (median 2.87/Mb) are similar across cohorts (Figure S4B-D).

Using three different identification tools, we discovered 62 driver genes. Large proportion of these genes (n = 13, 21%) including several classical driver genes such as *TP53*, *CTNNB1* and *DYRK1A* were identified by all three methods. In order to understand the novelty of these drivers. Among these genes, most of them were previously reported as driver gene except for *DYRK1A. DYRK1A* phosphorylates a component of DREAM complex (LIN52) which induce quiescence in cells and can be an important driver gene for HCC [1]. In addition to common drivers, a significant amount of driver genes was found in only a subset of methods which can be attributed to distinct features of driver genes captured by different methods (Table S4-S5, Figure S6A). In order to understand the novelty of drivers, a total of 88 literature reported drivers were curated from eight earlier studies (Table S1) and Cancer Gene Census (CGC). 33 genes overlap between literature drivers and our driver list (Figure S6B). Among the novel drivers (n = 29), 9 genes were found in the Cancer Gene Census (CGC) indicated in other cancer types (Figure S5, S6B, Supplementary Table 5). For example, *ATRX* is associated with

alternative lengthening of telomerase (ALT) which can lead to synthesis of telomeres without telomerase in sarcomas [2]. In addition to identification of the driver genes, TUSON Explorer and 20/20+ calculate the probability of oncogene (OG) or tumor suppressor gene (TSG) for each driver. In total, 30 candidate driver genes were classified as TSG and 9 candidate drivers were classified as OG. This list includes known oncogenes such as *CTNNB1*, *PI3KCA*, *NFE2L2*, *KRAS*, *EEF1A1* and *SF3B1* as well as new HCC oncogenes such as *IDH1*, *KCNN3* and *CRIP3* are novel HCC drivers. Among novel oncogenes, *KCNN3* encodes for SK3 protein which is a calcium-dependent potassium channel. The proliferative effect of activation of K channels has been reported by earlier studies and the inhibition of SK3 current can stop invasiveness of breast and colorectal cancer cells [3,4].

In order to explore the correlation between drivers and patient clinical phenotypes, we carried out the association analysis. When correlating driver genes with patient cohorts, only *KCNN3* was significantly enriched in a specific cohort (TCGA, 3.3%) in both univariate and multivariate models (Figure S6C, Fisher's test q value < 0.1). In general, many drivers tend to occur in patients with higher mutational burden and older age (above 63) such as *APOB* and *ALB* (Figure S6C). HBV positive tumors tend to have more *TP53* mutations (Figure S6C). One of the most frequently mutated driver gene *CTNNB1* shows the highest number of correlations. It is found to be correlated with older age, male gender as well as non-HBV etiology. Among novel drivers, *DOCK2* and *DYRK1A* mutations are associated with higher mutation burden and *HNF1A* is enriched in Europeans (Figure S6C).

Since carcinogenesis depends on gaining a full collection of hallmarks associated with cancer, drivers with similar functions from the same pathway may occur exclusively with each other. On the other hand, genetic changes with complementary functional roles might act synergistically and tend to co-occur in the same patient. By testing the association between the driver mutations, we identified 11 pairs of co-occurrence relationship and 2 pairs of mutual

exclusivities after multiple testing correction (Fisher's test q value < 0.1) (Figure S6D). For example, *CTNNB1* and *AXIN1*, activators of the WNT pathway, tend to occur mutually exclusively across patients. On the contrary, multiple driver pairs tends to co-occur. For example, *CTNNB1* and *NFE2L2*, oncogenes of the WNT and the oxidative stress pathways tend to co-occur in many patients, suggesting synergistic effect between these pathways in HCC.

Previous studies have identified several recurrently altered pathways such as WNT signalling, chromatin remodelling, growth factor signalling, TP53/cell cycle [5,6] . In order to identify frequently perturbed pathways, we conducted gene set enrichment analysis of 62 driver genes using g:Profiler [7] (based on Fisher's Exact test on the ranked gene list) and ConsensusPathDB [8] (based on the hypergeometric test). After combining pathways with similar function, 23 pathways were identified (13 from g:Profiler and 23 pathways from ConsensusPathDB, Figure S6E). In order to compare the list of pathways against previously published findings, we compiled a list of significantly perturbed pathways from 7 different studies[5,9–14] and compared with our list. As expected, classical cancer related pathways such as WNT, TP53/cell cycle and hypoxia were among pathways that were previously reported. In addition, we also identified 6 novel pathways including thyroid hormone signalling and hemostasis.

19 of novel HCC drivers were found to be in 17 known pathways (Figure S6E). For example, several novel drivers such as *TLE1, TNRC6B, APC, KMT2D* and *HNF1A* were found in the classical WNT pathway. In addition, 6 novel pathways include genes from both known and new drivers. 14 of previously known HCC drivers were found to be in 6 novel pathways (*e.g. PIK3CA* and *ALB*). Finally, 9 novel genes were found to be involved in the novel pathways. For example, *DOCK2*, *VAV3* and *TF* novel driver are involved in hemostasis (Figure S6E).

Similar to the observation in the combined cohort (n = 1349), most of the driver genes have similar frequencies in two cohorts except that *TP53, RB1* and *CDKN2A* have a significantly higher frequency in Asians, but *EEF1A1* occurs more frequently in Europeans (p-value < 0.05, Figure 1F). However, after multiple test correction, only *TP53* and *CDKN2A* remain significant (q-value = 0.016 and 0.08 respectively, Table S7).

Supplementary Note 2: Saturation analysis of driver genes

With larger cohort size, we were able to identify many driver genes that are in relative low frequency in the cohorts. In order to understand the relationship between sample size and the number of identified drivers, we down sample the cohort to various subsets and identified driver genes using MutSigCV. Interestingly, we found that the number of driver genes increased linearly with the sample size and has not reach saturation (Figure S7A). Since common drivers are easily detectable using small cohorts, we further break down the drivers according to their frequencies. Drivers above 5% were detected at a sample size around a few hundred samples and drivers in the range of 2-5% will be found around 1,000 samples (Figure S7A). The number of extremely rare drivers (f < 2%) has not yet reached saturation and is still rapidly increasing with sample size. In addition, we showed that these rare drivers tend to be late in the history of tumorigenesis (Fig S7B). Since not all regions of the tumor will have these subclonal rare mutations, it might harder to detect with small sample size. These indicate that there are many extremely rare drivers for HCC not yet found and this might explain the observation that a large proportion (14.6%) of the HCC patients have no driver genes identified.

Supplementary Note 3: Positive selection in driver genes

Positively selected driver genes in the history of tumorigenesis tend to have higher nonsynonymous mutation rate with more loss-of-function (*e.g.* truncating mutations) mutations in tumor suppressor genes and gain of function missense mutations in oncogenes. To test the

existence and type (truncating vs missense) of positive selection, we calculated the somatic rate of non-synonymous to synonymous mutations (d_N/d_S) for all the driver genes using dNdScv [15]. As expected, 90% of driver genes were positively selected with a significant $d_N/d_S > 1$ and most of them (45%) have a significant d_N/d_S ratio based only on truncated mutations indicating putative tumor suppressor roles (q value < 0.1, Figure S7D, S7D, Table S6). While 22 of drivers have significant d_N/d_S ratio for both truncating and missense mutations, 9 of them only have significance missense d_N/d_S ratios which implies that those drivers are likely to be oncogenes. When we plot truncated vs missense d_N/d_S values we can see the separation of well-known tumor suppressor genes (*e.g. AXIN1*, *TP53*) and oncogenes (*e.g. CTNNB1*) (Figure S7G). Interestingly, well known tumor suppressor gene *TP53* is significant in both truncating and missense d_N/d_S values because inactivation for *TP53* can be achieved by truncating mutations, deletions as well as hotspot missense changes in the DNA binding residues such as R249 (Figure S7G). Since oncogenes tend to have nonsynonymous gain-of-function mutations while tumor suppressor genes (TSG) often have truncating mutations spread across functional domains, a significant truncating d_N/d_S value will be a good indicator of TSG while significant missense d_N/d_S values often occur in oncogenes.

Supplementary Note 4: Collection and timing of mutational signatures

Several signatures (n = 10) including clock-like signatures (SBS1 and SBS5), smoking (SBS4), DNA mismatch repair (SBS6), aristolochic acid (AA) and aflatoxin B1 exposure (SBS22 and SBS24), liver cancer associated signatures (SBS12 and SBS16) and two signatures with unknown etiology (SBS17 and SBS23) have been identified in HCC [16–18].

To understand the timing of these mutational events along the history of tumorigenesis, we calculated the cancer cell fractions (CCF) for all mutations and deconvoluted signatures separately for early (CCF ≥ 0.8) and late mutations (CCF < 0.8, Figure S10B). Interestingly, the proportion of smoking (SBS4) and AA (SBS22) signatures were significantly lower in the

late stage of tumorigenesis compared to early mutations in both cohorts (Figure 10C). On the other hand, MSI and liver associated signatures SBS6 and SBS12 have higher proportions in the late stages of tumorigenesis, suggesting their active role throughout HCC initiation and progression (Figure S10C). Interestingly other signatures do not show a significant proportion difference between early and late mutations (Figure S10D).

Supplementary Note 5: RNA subtype comparison across ethnic backgrounds

A literature review revealed a list of RNA subtypes (n = 7) with varying levels of consistency between multiple cohorts [19–28]. For example, Hoshida et al (2009) described one well-recognized subtyping of HCC, including two subtypes (S1 and S2) with bad prognosis and one with good prognosis (S3) [23]. Boyault *et al.* identified six molecular subtypes (G1-G6) using patients of European descent with different levels of overlap with other RNA subtypes[22] .

Looking across molecular events for all the subtypes, we found that molecular differences between the basal split in P and M are highly similar to subsequent divergence in M1 and M2. This is true in both Asian and European cohorts. For example, *CTNNB1* mutation are highly enriched in subgroup M and M2 in both cohorts. This implies potential connections between genomic events happened in DNA and the transcriptomic changes in RNA. In general, we observed stronger genomic correlations in Europeans as compared with Asians (Figure 3F). For example, both arm and focal level SCNA scores, GII as well as genome doubling (GD) proportions are higher in P and M1 subtypes compared to M and M2 subtypes respectively in the European cohort.

Mapping the literature subtypes together with the molecular events onto the subtype ontology, we found both highly concordant and divergent events across the two cohorts. On one hand, we found good concordance between subtypes identified earlier using different ethnic populations. For example, the basal partition of P and M matches very well with the survival groups found in Lee et al 2004 [24]. Hoshida S1/S2 and S3 match with P1/P2 and M subgroups

in Asians, with S2 (P2) subtype not found in Europeans. The 6 subtypes found in Boyault et al 2007 [22] have a partial overlap in both Asian and European cohorts with G5/G6 subtypes matching well to subtype G in Asians and G2 in Europeans. In addition to molecular convergence, we also found features unique to specific ethnic background. For example, Yamashita EpCAM subtype [20] matches to P2 subtype in Asians, but P subtypes in Europeans. (Figure S16D). The integrative analysis between the two cohorts identified important ethnic similarities and differences between the two cohorts, providing an important base integrating information across multiple studies and ethnic backgrounds.

References

1. Litovchick L. DYRK1A protein kinase promotes quiescence and senescence through DREAM complex assembly. Genes & Development. 2011; 25: 801–13.

2. Lawlor RT, Veronese N, Pea A, Nottegar A, Smith L, Pilati C, *et al.* Alternative lengthening of telomeres (ALT) influences survival in soft tissue sarcomas: a systematic review with meta-analysis. BMC Cancer. 2019; 19: 232.

3. Gueguinou M, Crottès D, Chantôme A, Rapetti-Mauss R, Potier-Cartereau M, Clarysse L, *et al.* The SigmaR1 chaperone drives breast and colorectal cancer cell migration by tuning SK3-dependent Ca2+ homeostasis. Oncogene. 2017; 36: 3640.

4. Prevarskaya N, Skryma R, Shuba Y. Ion channels and the hallmarks of cancer. Trends in Molecular Medicine. 2010; 16: 107–21.

5. Ahn S-M, Jang SJ, Shim JH, Kim D, Hong S-M, Sung CO, *et al.* Genomic portrait of resectable hepatocellular carcinomas: Implications of RB1 and FGF19 aberrations for patient stratification. Hepatology. 2014; 60: 1972–82.

6. Cleary SP, Jeck WR, Zhao X, Chen K, Selitsky SR, Savich GL, *et al.* Identification of Driver Genes in Hepatocellular Carcinoma by Exome Sequencing. Hepatology (Baltimore, Md). 9; 58: 1693–702.

7. Reimand J, Arak T, Adler P, Kolberg L, Reisberg S, Peterson H, *et al.* g:Profiler—a web server for functional interpretation of gene lists (2016 update). Nucleic Acids Research. 2016; 44: W83–9.

8. Kamburov A, Wierling C, Lehrach H, Herwig R. ConsensusPathDB—a database for integrating human functional interaction networks. Nucleic Acids Research. 2009; 37: D623–8.

9. Ally A, Balasundaram M, Carlsen R, Chuah E, Clarke A, Dhalla N, *et al.* Comprehensive and Integrative Genomic Characterization of Hepatocellular Carcinoma. Cell. 6; 169: 1327-1341.e23.

10. Kan Z, Zheng H, Liu X, Li S, Barber TD, Gong Z, *et al.* Whole-genome sequencing identifies recurrent mutations in hepatocellular carcinoma. Genome Research. 2013; 23: 1422–33.

11. Schulze K, Imbeaud S, Letouze E, Alexandrov LB, Calderaro J, Rebouissou S, *et al.* Exome sequencing of hepatocellular carcinomas identifies new mutational signatures and potential therapeutic targets. Nat Genet. 2015; 47: 505–11.

12. Totoki Y, Tatsuno K, Covington KR, Ueda H, Creighton CJ, Kato M, *et al.* Trans-ancestry mutational landscape of hepatocellular carcinoma genomes. Nat Genet. 2014; 46: 1267–73.

13. Guichard C, Amaddeo G, Imbeaud S, Ladeiro Y, Pelletier L, Maad IB, *et al.* Integrated analysis of somatic mutations and focal copy-number changes identifies key genes and pathways in hepatocellular carcinoma. Nat Genet. 2012; 44: 694–8.

14. Dow M, Pyke RM, Tsui BY, Alexandrov LB, Nakagawa H, Taniguchi K, *et al.* Integrative genomic analysis of mouse and human hepatocellular carcinoma. Proceedings of the National Academy of Sciences. 2018; 115: E9879.

15. Martincorena I, Raine KM, Gerstung M, Dawson KJ, Haase K, Van Loo P, *et al.* Universal Patterns of Selection in Cancer and Somatic Tissues. Cell. 2018; 171: 1029-1041.e21.

16. Nault J-C, Martin Y, Caruso S, Hirsch TZ, Bayard Q, Calderaro J, *et al.* Clinical Impact of Genomic Diversity From Early to Advanced Hepatocellular Carcinoma. Hepatology. 2020; 71: 164–82.

17. Letouzé E, Shinde J, Renault V, Couchy G, Blanc J-F, Tubacher E, *et al.* Mutational signatures reveal the dynamic interplay of risk factors and cellular processes during liver tumorigenesis. Nature Communications. 2017; 8: 1315.

18. Lu Z-N, Luo Q, Zhao L-N, Shi Y, Wang N, Wang L, *et al.* The Mutational Features of Aristolochic Acid–Induced Mouse and Human Liver Cancers. Hepatology. 2020; 71: 929–42.

19. Roessler S, Jia H-L, Budhu A, Forgues M, Ye Q-H, Lee J-S, *et al.* A Unique Metastasis Gene Signature Enables Prediction of Tumor Relapse in Early-Stage Hepatocellular Carcinoma Patients. Cancer Research. 2010; 70: 10202.

20. Yamashita T, Forgues M, Wang W, Kim JW, Ye Q, Jia H, *et al.* EpCAM and α-Fetoprotein Expression Defines Novel Prognostic Subtypes of Hepatocellular Carcinoma. Cancer Research. 2008; 68: 1451.

21. Chiang DY, Villanueva A, Hoshida Y, Peix J, Newell P, Minguez B, *et al.* Focal Gains of Vascular Endothelial Growth Factor A and Molecular Classification of Hepatocellular Carcinoma. Cancer research. 2008; 68: 6779–88.

22. Boyault S, Rickman DS, Reyniès A de, Balabaud C, Rebouissou S, Jeannot E, *et al.* Transcriptome classification of HCC is related to gene alterations and to new therapeutic targets. Hepatology. 2007; 45: 42–52.

23. Hoshida Y, Nijman SMB, Kobayashi M, Chan JA, Brunet J-P, Chiang DY, *et al.* Integrative Transcriptome Analysis Reveals Common Molecular Subclasses of Human Hepatocellular Carcinoma. Cancer Res. 2009; 69: 7385–92.

24. Lee J-S, Chu I-S, Heo J, Calvisi DF, Sun Z, Roskams T, *et al.* Classification and prediction of survival in hepatocellular carcinoma by gene expression profiling. Hepatology. 2004; 40: 667–76.

25. Lee J-S, Heo J, Libbrecht L, Chu I-S, Kaposi-Novak P, Calvisi DF, *et al.* A novel prognostic subtype of human hepatocellular carcinoma derived from hepatic progenitor cells. Nat Med. 2006; 12: 410–6.

26. Candia J, Bayarsaikhan E, Tandon M, Budhu A, Forgues M, Tovuu L-O, *et al.* The genomic landscape of Mongolian hepatocellular carcinoma. Nature Communications. 2020; 11: 4383.

27. Chaisaingmongkol J, Budhu A, Dang H, Rabibhadana S, Pupacdi B, Kwon SM, *et al.* Common Molecular Subtypes Among Asian Hepatocellular Carcinoma and Cholangiocarcinoma. Cancer Cell. 2017;

28. Xue R, Chen L, Zhang C, Fujita M, Li R, Yan S-M, *et al.* Genomic and Transcriptomic Profiling of Combined Hepatocellular and Intrahepatic Cholangiocarcinoma Reveals Distinct Molecular Subtypes. Cancer Cell. 2019; 35: 932-947.e8.

Table S1: Driver genes collected from the literature

Driver	Cleary_et_al (2013)	Kan_et_al (2013)	Totoki_et_al (2014)	Ahn_et_al (2014)	Schulze_et_al (2015)	Fujimato_et_al (2016)	TCGA (2017)	Chaudhary_et_al (2019)	#of_studies
TP53	1	1	1	1	1	1	1	1	8
CTNNB1	1	1	1	1	1	1	1	1	8
AXIN1	0	1	1	1	1	1	1	1	7
RPS6KA3	0	0	1	1	1	1	1	1	6
RB1	0	0	1	1	1	1	1	1	6
NFE2L2	0	0	1	0	1	1	1	1	5
CDKN2A	0	0	1	0	1	1	1	1	5
ARID1A	0	0	1	0	1	1	1	1	5
ARID2	0	0	1	0	1	1	1	0	4
ACVR2A	0	0	1	0	1	1	1	1	5
ALB	0	0	0	1	1	1	1	1	5
KEAP1	1	0	0	0	1	0	1	0	3
BRD7	0	0	1	0	0	1	0	0	2
CCND1	0	0	1	0	0	1	0	0	2
PTEN	0	0	1	0	0	1	0	0	2
CDKN1A	0	0	1	0	1	0	0	0	2
APOB	0	0	0	0	0	1	1	0	2
G6PC	0	0	1	0	0	1	0	0	2
RPL22	0	0	0	0	1	1	0	0	2
LRP1B	0	1	0	0	0	1	0	0	2
BAP1	0	0	0	0	0	1	1	0	2
COL11A1	0	1	0	0	0	0	0	0	1
AHCTF1	0	0	0	0	0	0	1	0	1
MEN1	0	0	1	0	0	0	0	0	1
IL6ST	0	0	0	0	0	0	1	0	1
RP1L1	0	0	0	0	0	0	1	0	1
SETDB1	0	0	0	0	0	1	0	0	1
TMEM51	1	0	0	0	0	0	0	0	1
CDKN2B	0	0	1	0	0	0	0	0	1
PER3	0	0	0	0	0	1	0	0	1
EYS	0	0	0	0	0	1	0	0	1
BRD9	1	0	0	0	0	0	0	0	1
MUC17	0	0	0	0	0	1	0	0	1
GPATCH4	0	0	0	0	0	0	1	0	1
ERGIC1	0	0	0	0	0	1	0	0	1
CACNA2D4	0	1	0	0	0	0	0	0	1
KRAS	0	0	0	0	0	0	1	0	1
ADRA1A	0	0	0	0	0	1	0	0	1

ADCY2	0	1	0	0	0	0	0	0	1
WDTC1	0	0	0	0	0	1	0	0	1
LZTR1	0	0	0	0	0	0	1	0	1
CPS1	0	0	0	0	0	1	0	0	1
CDKN1B	0	0	0	1	0	0	0	0	1
CPA2	1	0	0	0	0	0	0	0	1
JAK1	0	1	0	0	0	0	0	0	1
TSC1	0	0	1	0	0	0	0	0	1
TMEM170A	1	0	0	0	0	0	0	0	1
TMEM99	0	0	1	0	0	0	0	0	1
GJA1	1	0	0	0	0	0	0	0	1
HNF4A	0	0	0	0	0	1	0	0	1
CYP2E1	0	0	1	0	0	0	0	0	1
PIK3CA	0	0	0	0	0	0	1	0	1
FAM5C	0	1	0	0	0	0	0	0	1
TSC2	0	0	1	0	0	0	0	0	1
NCOR1	0	0	1	0	0	0	0	0	1
KRTAP5-11	0	0	0	0	0	1	0	0	1
SLC10A1	0	1	0	0	0	0	0	0	1
FGF19	0	0	1	0	0	0	0	0	1
TTC28	0	0	0	0	0	1	0	0	1
ASH1L	0	0	0	0	0	1	0	0	1
HIST1H1C	0	0	0	0	0	0	1	0	1
MAP2K3	0	0	1	0	0	0	0	0	1
TBL1XR1	0	0	0	0	0	1	0	0	1
GALNT11	0	0	1	0	0	0	0	0	1
PRKACA	0	0	0	0	0	1	0	0	1
NSMCE2	0	0	0	0	0	1	0	0	1
SRCAP	0	0	1	0	0	0	0	0	1
EEF1A1	0	0	0	0	0	0	1	0	1
SF3B1	0	0	0	0	0	0	1	0	1
IGSF3	1	0	0	0	0	0	0	0	1
SMARCA4	0	0	0	0	0	0	1	0	1
EPS15	0	1	0	0	0	0	0	0	1
NRAS	0	0	0	0	0	0	1	0	1
SELPLG	0	0	0	1	0	0	0	0	1
HNRNPA2B1	0	0	1	0	0	0	0	0	1
FCRL1	0	0	1	0	0	0	0	0	1
TERT	0	0	1	0	0	0	0	0	1
ESRRG	0	0	0	0	0	1	0	0	1

PCMTD1	1	0	0	0	0	0	0	0	1
ATAD3B	1	0	0	0	0	0	0	0	1
TTLL2	1	0	0	0	0	0	0	0	1
AR	1	0	0	0	0	0	0	0	1
VCX	0	0	0	1	0	0	0	0	1
MTAP	0	0	0	0	0	1	0	0	1
AZIN1	0	0	0	0	0	0	1	0	1
CREB3L3	0	0	0	0	0	0	1	0	1
ADH1B	0	0	1	0	0	0	0	0	1
MACROD2	0	0	0	0	0	1	0	0	1

Table S2: Study cohorts

Data source	Project name	Cohort	N_Samples*	Sequencing approach	Sequencing center	Hypermutated patients
TCGA	LIHC	TCGA	373	WES	Broad	7
ICGC	LIRI-JP	LIRI-JP	270	WES/WGS	RIKEN	1
ICGC	LINC-JP	LINC-JP	244	WES/WGS	National Cancer Center	0
ICGC	LICA-FR	French	242	WES	Institut National du Cancer	1
Ahn et al. 2014	-	Korean	231	WES	Asan Bio-Resource Center, Korea	2

* Hypermutated patients whose total mutation number in coding regions is greater than 1000 were excluded from this study.

Table S3: Summary of clinical features across cohorts

	French	Korean	LIRI-JP	LINC-JP	TCGA	Total
**Age**						
Median (range)	65 (17-90)	55 (26-80)	69 (31-89)	66 (23-85)	61 (16-90)	63 (16-90)
**Gender**						
Male	190	174	201	182	246	993
Female	47	55	68	62	120	352
Missing	4	0	0	0	0	4
**Tumor Stage**						
Early	127	229	163	85	254	858
Late	55	0	106	110	89	360
Missing	59	0	0	49	23	131
**Etiology**						
HBV	31	167	66	*NA*	99	363
HCV	59	21	145	*NA*	48	273
HBV+HCV	2	0	4	*NA*	7	13
NBNC	143	41	53	*NA*	193	430
Missing	6	0	1	244	19	270
**Ethnicity**						
European	241	0	0	0	179	420
Asian	0	229	269	244	159	901
Missing	0	0	0	0	28	28

Table S4: Significantly mutated genes from three methods

MUTSIGCV															
gene	expr	reptime	hic	N_nonsilent	N_silent	N_noncoding	n_nonsilent	n_silent	n_noncoding	nnei	x	X	p	q	Frequency
TP53	2069567	213	34	4167061	1150697	0	401	6	0	3	8	3499306	1.18E-14	5.41E-12	0.286879
CTNNB1	305811	448	8	7549004	2188078	0	407	10	0	5	18	6855618	5.44E-15	2.62E-12	0.283173
APOB	222162	698	-11	43727835	12136953	0	147	24	0	6	34	18056365	4.20E-07	1.72E-04	0.107487
ARID1A	975965	182	27	21581302	6480596	0	137	3	0	1	4	9370154	1.32E-14	5.93E-12	0.0919199
ALB	315782	613	8	6115017	1525719	0	129	7	0	4	13	5445913	0	0	0.0889548
ARID2	820796	602	10	17469550	5145086	0	112	8	0	0	8	5145086	0	0	0.0733877
AXIN1	1444992	205	40	8215410	2424153	0	95	3	0	50	109	119319050	0	0	0.0674574
COL11A1	37620	742	-20	18165634	5476940	0	72	12	0	13	32	19515983	9.45E-06	3.64E-03	0.0622683
NFE2L2	372300	145	36	5872197	1550001	0	67	2	0	50	65	72286165	0	0	0.0437361
RPS6KA3	242944	NaN	36	7418151	1918278	0	65	0	0	8	13	13123072	0	0	0.0429948
TSC2	1722112	181	44	17369724	5253006	0	56	5	0	45	55	73029464	0	0	0.0422535
DOCK2	94542	680	33	18520421	4543432	0	53	8	0	20	27	32309899	5.88E-08	2.47E-05	0.0422535
RB1	349930	450	41	9318892	2380985	0	59	1	0	50	72	85283780	0	0	0.0407709
SETD2	593886	365	46	24742009	6731510	0	42	7	0	50	63	75418543	2.93E-05	1.10E-02	0.0348406
ACVR2A	90992	951	-2	5100569	1301785	0	49	2	0	50	101	77648440	0	0	0.0348406
KEAP1	1996822	207	30	5949090	1719975	0	44	4	0	13	17	19011457	0	0	0.0340993
BAP1	1078407	229	62	7075505	2046433	0	35	3	0	45	46	67630766	0	0	0.0252039
CDKN2A	225405	357	-15	3217365	837729	0	30	1	0	38	59	57654911	0	0	0.02298
SF3B1	736523	276	30	12804708	3524937	0	29	2	0	30	43	59716183	9.59E-05	3.07E-02	0.0222387
NEFH	723458	203	35	9637256	2815363	0	25	9	0	19	30	24933567	6.27E-05	2.19E-02	0.0222387
VAV3	132139	554	-11	8707795	2117930	0	28	1	0	24	47	45565173	7.93E-05	2.65E-02	0.0214974
BRD7	303953	520	14	6612798	1582377	0	29	1	0	16	24	28903674	5.55E-15	2.62E-12	0.0214974
PTEN	259678	300	34	4084772	949696	0	28	2	0	10	9	9917848	4.44E-11	1.95E-08	0.0207561
HNF1A	1225663	380	40	5947741	1879157	0	35	4	0	50	50	67672585	0	0	0.0207561
IL6ST	317374	438	51	9070676	2329723	0	24	3	0	50	75	88791180	0	0	0.0200148
TF	489558	523	26	6936558	1809009	0	25	2	0	50	73	71738471	0	0	0.0192735
TSC1	1919595	461	33	11353184	3131029	0	23	1	0	22	11	28177912	2.96E-08	1.27E-05	0.017791
DYRK1A	314682	366	47	7767542	2038339	0	23	0	0	13	15	20894661	1.18E-06	4.74E-04	0.0170497
HNF4A	769430	376	16	5172066	1469061	0	21	2	0	41	60	64750651	0	0	0.0163084
CDKN1A	904665	228	37	1529766	505875	0	20	1	0	11	14	19271814	0	0	0.0155671
CYP2E1	563924	525	-10	4822675	1312577	0	18	1	0	50	81	67346127	0	0	0.0148258
KCNN3	1958370	559	34	7021545	2059923	0	19	4	0	32	23	44179750	8.98E-05	2.92E-02	0.0140845
ERRFI1	387339	207	26	4368062	1301785	0	20	3	0	12	22	16587304	5.11E-05	1.82E-02	0.0140845
RPL22	627248	240	31	1317973	304874	0	18	0	0	50	52	69759488	0	0	0.0133432
HNRNPA2[illegible]	915230	445	43	3581595	894387	0	18	2	0	15	25	18614851	3.18E-05	1.18E-02	0.0133432
SELPLG	448698	280	40	3630159	1295040	0	15	3	0	28	39	39423176	0	0	0.0126019
SLC30A1	380154	236	22	4734990	1448826	0	17	1	0	50	60	76292695	0	0	0.0118606
ZFP36L1	543449	259	46	3128331	1003656	0	14	2	0	50	65	70474458	0	0	0.0111193
KRAS	259193	512	16	2312186	557137	0	15	1	0	35	52	48174139	9.87E-05	3.10E-02	0.0111193
FRG1	49764	1059	-15	2676416	605701	0	18	0	0	50	140	99007157	0	0	0.0111193
ADH1B	332785	854	8	3644998	1057616	0	14	0	0	21	32	28395101	7.79E-05	2.65E-02	0.0111193
PHF10	233648	447	3	5004790	1247825	0	12	2	0	27	53	56342334	1.12E-04	3.47E-02	0.0103781
HP	438866	372	19	3988993	1057616	0	14	0	0	5	11	16993353	8.02E-05	2.65E-02	0.0103781
CRIP3	1293543	363	51	2073413	528808	0	20	0	0	28	46	42007860	0	0	0.0103781
CELF1	1011926	219	40	5162623	1316624	0	15	2	0	45	60	76343957	0	0	0.0103781
IRF2	207866	400	55	3488514	890340	0	11	2	0	50	67	77957361	0	0	0.00963677
EFNB2	167697	1267	2	3245694	882246	0	12	1	0	50	50	67141079	2.14E-04	6.50E-02	0.00963677
CDKN1B	791330	136	44	1920976	527459	0	12	2	0	8	10	10144480	2.41E-06	9.49E-04	0.00889548
FCN1	237562	489	35	3225459	882246	0	12	0	0	11	4	14751315	2.95E-04	8.83E-02	0.00815419
CELA1	504747	239	33	2536120	729809	0	11	0	0	50	59	72392736	0	0	0.00815419
HMBS	1390854	195	58	3576199	1029287	0	11	0	0	28	25	39205987	4.25E-05	1.54E-02	0.0074129
EFNA5	78424	628	21	2260924	584117	0	10	1	0	50	70	80307319	0	0	0.0074129
FUBP1	373296	256	1	6359186	1787425	0	9	2	0	50	77	96649105	0	0	0.00667161
EHD3	102236	884	15	5143737	1444779	0	8	2	0	50	84	77377291	0	0	0.00667161
WDR5B	416095	478	34	3132378	886293	0	9	1	0	50	60	61448299	0	0	0.00593032
KRTAP4-1	996799	468	-7	1246476	331854	0	8	0	0	50	74	64954350	0	0	0.00593032
KIAA0040	NaN	NaN	NaN	967233	246867	0	8	0	0	50	173	150476903	0	0	0.00444774
BAIAP2L1	609918	249	36	5077636	1349000	0	4	2	0	36	48	50532191	0	0	0.00370645
SPATA3	NaN	NaN	NaN	1829244	562533	0	6	1	0	37	129	106227005	0	0	0.00296516
NSUN3	85578	1378	-9	3317191	903830	0	3	1	0	25	49	46324660	0	0	0.00222387
NADK	1511678	243	30	4380203	1224892	0	3	1	0	50	40	70571586	0	0	0.00222387
MLF2	2816186	232	48	2468670	683943	0	4	0	0	50	85	95985397	0	0	0.00222387
GNRH1	178665	515	21	917320	256310	0	2	0	0	50	57	78805882	0	0	0.00074129

TUSON			
Gene	TSG q-value	OG q-value	Frequency
ACVR2A	0.000659841	1	0.0348406
ALB	5.95E-16	1	0.0889548
ARID1B	8.20E-25	1	0.0200148
AXIN1	7.39E-21	1	0.0674574
BAP1	0.000799981	1	0.0252039
BRD7	0.000118092	1	0.0214974
CDKN2A	0.006011515	1	0.02298
CTNNB1	1	1.18E-05	0.283173
DYRK1A	0.017590403	1	0.0170497
NFE2L2	1	0.025948703	0.0437361
PTEN	0.035324469	1	0.0207561
RB1	4.78E-12	1	0.0407709
RPS6KA3	2.09E-10	1	0.0429948
TP53	1.57E-54	1	0.286879
TSC1	0.07215699	1	0.017791
TSC2	2.18E-06	1	0.0422535

20/20+				
Gene	oncogene qval	TSG qval	driver qval	Frequency
CTNNB1	0	1	0	0.283173
TSC1	0.997869962	0.007692308	0.005263158	0.017791
TSC2	0.999513824	0.007692308	0.005263158	0.0422535
SETD2	0.997869962	0.007692308	0.005263158	0.0348406
TP53	0.899661226	0.038095238	0.005263158	0.286879
BAP1	0.997869962	0.007692308	0.005263158	0.0252039
PIK3CA	0	1	0.005263158	0.0244626
KMT2B	0.997869962	0.007692308	0.005263158	0.0214974
BRD7	0.997869962	0.007692308	0.005263158	0.0214974
ARID1A	0.997869962	0.007692308	0.005263158	0.0919199
RB1	0.997869962	0.007692308	0.005263158	0.0407709
IDH1	0	1	0.005263158	0.0111193
RPS6KA3	0.997869962	0.007692308	0.005263158	0.0429948
NFE2L2	0	1	0.005263158	0.0437361
AXIN1	0.997869962	0.007692308	0.005263158	0.0674574
DYRK1A	0.997869962	0.007692308	0.005263158	0.0170497
ARID2	0.997869962	0.007692308	0.005263158	0.0733877
KRAS	0	1	0.005263158	0.0111193
CDKN2A	0.994321142	0.023529412	0.01	0.02298
APC	0.913981245	0.053571429	0.016	0.0303929
KDM6A	0.997869962	0.021428571	0.016	0.0126019
PBRM1	0.997869962	0.027777778	0.016	0.0192735
KMT2D	0.997869962	0.023529412	0.016	0.0474426
CYP2E1	0.997869962	0.036842105	0.023076923	0.0148258
ATRX	0.997732972	0.044	0.02962963	0.0200148
TLE1	0.980085898	0.061764706	0.03030303	0.0126019
EEF1A1	0	1	0.03030303	0.0207561
ALB	0.997869962	0.044	0.03030303	0.0889548
CDKN1A	0.997869962	0.038095238	0.03030303	0.0155671
HNF1A	0.997869962	0.05	0.038235294	0.0207561
GSE1	0.997869962	0.044	0.042857143	0.0148258
SRCAP	0.990203507	0.061764706	0.051351351	0.0296516
TNRC6B	0.997869962	0.053571429	0.051351351	0.0259451
RPL22	0.997869962	0.061764706	0.054761905	0.0133432
SF3B1	0	0.999391117	0.070212766	0.0222387
PTPN3	0.997869962	0.076315789	0.073469388	0.0155671
NCOR1	0.865929204	0.193939394	0.073469388	0.0244626
RAPGEF2	0.984367899	0.117307692	0.08	0.0103781
ARID1B	0.997869962	0.117307692	0.098275862	0.0200148

Table S5: Concensus driver list and novel status

Gene	TUSON	MutSigCV	2020plus	CGC	Reported	Status
APC	0	0	1	1	0	Novel
ARID1B	1	0	1	1	0	Novel
ATRX	0	0	1	1	0	Novel
CELF1	0	1	0	0	0	Novel
CRIP3	0	1	0	0	0	Novel
DOCK2	0	1	0	0	0	Novel
DYRK1A	1	1	1	0	0	Novel
ERRFI1	0	1	0	0	0	Novel
FRG1	0	1	0	0	0	Novel
GSE1	0	0	1	0	0	Novel
HNF1A	0	1	1	1	0	Novel
HP	0	1	0	0	0	Novel
IDH1	0	0	1	1	0	Novel
KCNN3	0	1	0	0	0	Novel
KDM6A	0	0	1	1	0	Novel
KMT2B	0	0	1	0	0	Novel
KMT2D	0	0	1	1	0	Novel
NEFH	0	1	0	0	0	Novel
PBRM1	0	0	1	1	0	Novel
PHF10	0	1	0	0	0	Novel
PTPN3	0	0	1	0	0	Novel
RAPGEF2	0	0	1	0	0	Novel
SETD2	0	1	1	1	0	Novel
SLC30A1	0	1	0	0	0	Novel
TF	0	1	0	0	0	Novel
TLE1	0	0	1	0	0	Novel
TNRC6B	0	0	1	0	0	Novel
VAV3	0	1	0	0	0	Novel
ZFP36L1	0	1	0	0	0	Novel
ACVR2A	1	1	0	1	1	Reported
ADH1B	0	1	0	0	1	Reported
ALB	1	1	1	0	1	Reported
APOB	0	1	0	0	1	Reported
ARID1A	0	1	1	1	1	Reported
ARID2	0	1	1	1	1	Reported
AXIN1	1	1	1	1	1	Reported
BAP1	1	1	1	1	1	Reported
BRD7	1	1	1	0	1	Reported
CDKN1A	0	1	1	1	1	Reported
CDKN2A	1	1	1	1	1	Reported
COL11A1	0	1	0	0	1	Reported
CTNNB1	1	1	1	1	1	Reported
CYP2E1	0	1	1	0	1	Reported
EEF1A1	0	0	1	0	1	Reported
HNF4A	0	1	0	0	1	Reported
HNRNPA2B1	0	1	0	1	1	Reported
IL6ST	0	1	0	1	1	Reported
KEAP1	0	1	0	1	1	Reported

KRAS	0	1	1	1	1	Reported
NCOR1	0	0	1	1	1	Reported
NFE2L2	1	1	1	1	1	Reported
PIK3CA	0	0	1	1	1	Reported
PTEN	1	1	0	1	1	Reported
RB1	1	1	1	1	1	Reported
RPL22	0	1	1	1	1	Reported
RPS6KA3	1	1	1	0	1	Reported
SELPLG	0	1	0	0	1	Reported
SF3B1	0	1	1	1	1	Reported
SRCAP	0	0	1	0	1	Reported
TP53	1	1	1	1	1	Reported
TSC1	1	1	1	1	1	Reported
TSC2	1	1	1	1	1	Reported

Table S6: dNdScv results

gene_name	n_syn	n_mis	n_non	n_spl	n_ind	wmis_cv	wnon_cv	wspl_cv	wind_cv	pmis_cv	ptrunc_cv	pallsubs_c	pind_cv	qmis_cv	qtrunc_cv	qallsubs_cv	pglobal_cv	qglobal_cv	issignif_mis	issignif_trunc
CTNNB1	9	390	2	0	13	30.45708	1.94939	1.94939	1.817494	0	0.417982	0	0.189644	0	0.470230113	0	0	0	1	0
KRAS	1	15	0	0	0	10.00042	0	0	0	8.82E-07	0.602203	3.71E-06	1	1.11E-05	0.62194719	1.11E-05	5.01E-05	0.000137363	1	0
PIK3CA	2	34	0	0	0	4.826334	0	0	0	2.95E-05	0.251312	2.04E-05	1	0.000155	0.304473661	5.36E-05	0.000241158	0.000607717	1	0
EEF1A1	5	27	0	0	0	4.531896	0	0	0	1.80E-05	0.363415	3.72E-05	1	0.000103	0.423983791	9.02E-05	0.000416982	0.00101038	1	0
HNRNPA2B1	2	12	0	0	6	3.68723	0	0	1.853038	0.006588	0.38388	0.01185	0.200558	0.019598	0.439717475	0.015883482	0.016735751	0.025103627	1	0
IDH1	1	15	0	0	0	4.724699	0	0	0	0.001001	0.492397	0.002495	1	0.003319	0.543467073	0.004136292	0.017448138	0.025563551	1	0
SF3B1	2	28	0	1	0	3.590315	1.390097	1.390097	0	0.001338	0.769352	0.004817	1	0.004214	0.769351945	0.007225211	0.030517575	0.040906536	1	0
TF	2	16	0	1	7	3.095081	2.227982	2.227982	1.094857	0.011781	0.500335	0.041632	0.328446	0.030925	0.543467073	0.047687284	0.072365403	0.087673469	1	0
SLC30A1	1	11	0	0	5	3.240331	0	0	1.076075	0.022088	0.551115	0.051822	0.34064	0.051069	0.578670658	0.057276801	0.088913879	0.101846807	1	0
TP53	9	260	44	37	57	33.43668	113.3476	113.3476	15.81667	0	0	0	3.85E-05	0	0	0	0	0	1	1
RPS6KA3	0	26	13	9	17	6.775784	53.76363	53.76363	2.508229	1.29E-05	0	0	0.116829	9.00E-05	0	0	0	0	1	1
ARID1A	4	32	28	7	68	2.476697	39.80203	39.80203	3.252139	0.012741	0	0	0.065105	0.032107	0	0	0	0	1	1
CDKN2A.p16INK4a	1	14	6	3	7	9.400846	104.5743	104.5743	4.874553	2.78E-06	9.91E-14	6.33E-15	0.038501	2.92E-05	8.92E-13	4.98E-14	8.99E-15	7.08E-14	1	1
NFE2L2	2	57	2	0	8	12.16548	5.974582	5.974582	1.443291	4.55E-15	0.064515	4.54E-14	0.254311	9.56E-14	0.086477961	3.18E-13	3.82E-13	2.67E-12	1	1
ALB	6	31	4	5	67	4.207949	11.35164	11.35164	12.0083	1.55E-05	2.40E-06	1.39E-07	0.000296	9.74E-05	9.44E-06	6.04E-07	1.03E-09	5.87E-09	1	1
CDKN2A.p14arf	2	13	1	3	3	8.480843	42.48925	42.48925	2.466074	6.10E-06	7.36E-06	2.71E-08	0.168091	5.49E-05	2.73E-05	1.31E-07	9.20E-08	4.46E-07	1	1
RPL22	0	7	2	3	4	8.427837	50.86905	50.86905	3.390055	0.000605	5.29E-07	1.44E-07	0.098729	0.002117	3.03E-06	6.04E-07	2.71E-07	1.14E-06	1	1
CDKN1A	1	11	5	0	7	6.205216	36.31413	36.31413	3.845753	0.000371	1.98E-06	4.04E-07	0.063681	0.001459	8.90E-06	1.41E-06	4.75E-07	1.87E-06	1	1
CRIP3	0	16	3	0	1	11.31012	24.01032	24.01032	0.50151	4.59E-07	0.000633	1.60E-07	0.587637	7.23E-06	0.001534412	6.29E-07	1.61E-06	5.98E-06	1	1
ACVR2A	2	21	4	2	19	4.95619	15.3042	15.3042	4.041354	0.000114	2.68E-05	3.30E-06	0.043968	0.000512	8.88E-05	1.07E-05	2.43E-06	8.50E-06	1	1
FRG1	0	11	1	5	1	6.961139	31.62983	31.62983	0.422121	0.000302	9.26E-07	3.14E-07	0.62016	0.001268	4.86E-06	1.16E-06	3.20E-06	1.06E-05	1	1
DYRK1A	0	12	5	2	4	2.991107	23.65111	23.65111	0.572405	0.040949	1.16E-06	6.86E-06	0.504536	0.083219	5.64E-06	1.96E-05	4.70E-05	0.000134468	1	1
KEAP1	4	33	2	1	8	4.716855	6.35277	6.35277	1.399415	7.99E-06	0.02071	2.13E-05	0.262155	6.29E-05	0.031822219	5.36E-05	7.30E-05	0.000191738	1	1
HNF1A	5	19	1	4	7	2.684839	9.676925	9.676925	1.154306	0.010978	0.000563	0.000591	0.314608	0.03007	0.001419458	0.001283642	0.001782809	0.004102132	1	1
COL11A1	11	59	1	7	3	2.844399	2.911227	2.911227	0.180312	7.56E-05	0.022916	0.00027	0.733975	0.000366	0.033574382	0.000630153	0.001888283	0.004102132	1	1
PTEN	2	13	2	1	12	3.566826	9.229142	9.229142	3.247404	0.006844	0.008201	0.003037	0.077003	0.019598	0.014453288	0.004905537	0.002188937	0.004596768	1	1
VAV3	1	23	2	3	0	4.497824	8.350784	8.350784	0	0.000484	0.001911	0.000306	1	0.001792	0.00376173	0.000689451	0.00278555	0.005660957	1	1
DOCK2	9	41	6	4	0	1.954805	4.924129	4.924129	0	0.026482	0.000448	0.001342	1	0.057529	0.001227242	0.002641448	0.010215378	0.017876911	1	1
HNF4A	2	13	2	1	5	3.146097	8.386384	8.386384	1.150834	0.014959	0.010744	0.00677	0.32334	0.036247	0.017812114	0.009478527	0.015595938	0.023964491	1	1
ADH1B	0	8	0	2	5	3.817001	12.46681	12.46681	1.453847	0.022697	0.016941	0.011147	0.26418	0.051069	0.0273657	0.015267227	0.02010709	0.028789697	1	1
HP	0	8	1	1	3	3.241289	9.934448	9.934448	0.740379	0.045623	0.02668	0.026574	0.450336	0.08982	0.036718758	0.032195149	0.064928696	0.081810157	1	1
APOB	23	89	8	0	45	1.565646	2.224987	2.224987	1.077962	0.034098	0.065916	0.050634	0.315771	0.071605	0.086514142	0.056963632	0.082116559	0.095802652	1	1
ARID2	8	27	35	6	43	1.583567	29.37546	29.37546	2.560544	0.17365	0	0	0.106421	0.266827	0	0	0	0	0	1
AXIN1	3	13	40	8	33	1.851751	81.63297	81.63297	4.180609	0.177991	0	0	0.03723	0.266986	0	0	0	0	0	1
RB1	2	11	13	9	24	1.693618	30.36129	30.36129	2.824438	0.288705	0	0	0.09209	0.413373	0	0	0	0	0	1
BRD7	1	5	12	3	8	1.184904	32.29545	32.29545	1.339409	0.788371	4.48E-13	1.92E-13	0.273381	0.82779	3.53E-12	1.21E-12	1.66E-12	1.05E-11	0	1
TSC2	5	23	14	2	16	1.751647	13.92759	13.92759	0.967516	0.139113	3.75E-10	1.51E-09	0.350529	0.230635	2.63E-09	8.67E-09	1.19E-08	6.23E-08	0	1
TSC1	1	9	10	1	3	1.477876	21.04405	21.04405	0.281535	0.476179	9.08E-09	1.74E-08	0.659857	0.588221	5.72E-08	9.12E-08	2.21E-07	9.95E-07	0	1
TNRC6B	8	13	11	0	5	0.715671	7.056031	7.056031	0.298062	0.405178	1.54E-05	3.40E-06	0.642145	0.554917	5.40E-05	1.07E-05	3.06E-05	9.38E-05	0	1
BAP1	3	12	7	1	14	1.893379	14.5863	14.5863	2.096726	0.170471	2.34E-06	1.43E-05	0.156075	0.266827	9.44E-06	3.92E-05	3.13E-05	9.38E-05	0	1
ERRFI1	3	6	4	0	10	1.293702	16.02889	16.02889	2.361323	0.644753	0.000307	0.001412	0.136185	0.72668	0.00092094	0.002694911	0.001837202	0.004102132	0	1
SETD2	7	24	5	4	9	1.180003	5.849223	5.849223	0.383611	0.645938	0.000292	0.000868	0.585088	0.72668	0.000918533	0.001764761	0.004361764	0.008587223	0	1
KDM6A	2	6	3	3	3	0.758659	7.91666	7.91666	0.232781	0.632504	0.000883	0.00085	0.693314	0.72668	0.001989188	0.001764761	0.00496955	0.009487323	0	1
CYP2E1	1	8	3	1	4	2.221382	15.06716	15.06716	0.885257	0.15357	0.000515	0.002077	0.396874	0.248075	0.001352156	0.003701113	0.00667784	0.012373644	0	1
TLE1	3	6	4	1	4	0.918869	9.19844	9.19844	0.567208	0.878903	0.000884	0.002115	0.506696	0.893079	0.001989188	0.003701113	0.008399993	0.015119987	0	1
APC	8	20	7	1	6	0.876359	4.71889	4.71889	0.230652	0.719151	0.001861	0.002179	0.687425	0.781147	0.00376173	0.003709558	0.011238113	0.019135165	0	1
KMT2D	9	41	7	3	8	1.584574	4.929111	4.929111	0.157933	0.133166	0.000445	0.002078	0.746075	0.226742	0.001227242	0.003701113	0.011579227	0.01919714	0	1
PHF10	2	3	4	0	5	0.708046	8.725532	8.725532	1.095484	0.619146	0.003377	0.006139	0.336029	0.72668	0.006446625	0.00879	0.014819136	0.02354283	0	1
GSE1	5	4	3	1	7	0.367834	4.509915	4.509915	0.628329	0.081249	0.026314	0.004413	0.472134	0.150549	0.036718758	0.006807582	0.014947828	0.02354283	0	1
PTPN3	1	12	2	3	4	2.23033	8.752955	8.752955	0.478465	0.114791	0.001561	0.005889	0.546058	0.200884	0.003277663	0.008628722	0.021674482	0.029947212	0	1
CELF1	2	10	3	1	1	2.359675	10.77176	10.77176	0.21229	0.089204	0.00156	0.00443	0.733447	0.160567	0.003277663	0.006807582	0.021866218	0.029947212	0	1
RAPGEF2	3	5	3	1	3	0.507277	4.648782	4.648782	0.218659	0.241522	0.026811	0.012475	0.703727	0.353858	0.036718758	0.016373464	0.050351027	0.066085723	0	1
KMT2B	5	17	5	1	3	1.122869	5.068462	5.068462	0.120761	0.777185	0.005045	0.014683	0.788416	0.82779	0.0093477	0.018877913	0.063192106	0.081246993	0	1

PBRM1	3	11	4	1	8	1.016902	5.155611	5.155611	0.517535	0.972298	0.01029	0.02395	0.517867	0.972298	0.017520935	0.029585507	0.066849963	0.082579366	0	1
ARID1B	7	13	5	0	3	0.733319	4.063142	4.063142	0.143226	0.451492	0.019852	0.019836	0.766589	0.586383	0.031267531	0.024993953	0.078861024	0.093740463	0	1
SRCAP	7	25	5	1	4	1.307991	4.411561	4.411561	0.13535	0.450375	0.008259	0.030177	0.771522	0.586383	0.014453288	0.035870895	0.110825185	0.124678333	0	1
ATRX	5	16	4	2	2	0.936193	3.679751	3.679751	0.087709	0.874544	0.021227	0.037007	0.828012	0.893079	0.031840792	0.043175332	0.137443233	0.151910942	0	1

Table S7: Comparison of driver gene frequencies between Asian and European cohorts

Gene	Asian_percentage	European_percentage	p_value	q_value
CDKN2A	6.451612903	0	3.83E-04	0.01646234
TP53	36.77419355	23.49726776	0.00378959	0.0814762
EEF1A1	1.290322581	7.650273224	0.02454249	0.26383182
RB1	10.25641026	4.395604396	0.0225475	0.26383182
BRD7	5.128205128	1.111111111	0.08678317	0.74633523
AXIN1	11.53846154	7.142857143	0.11764858	0.78875208
CDKN1A	0.64516129	4.395604396	0.1284015	0.78875208
BAP1	3.846153846	7.182320442	0.21224555	0.85080444
HNF1A	0.64516129	3.846153846	0.22241459	0.85080444
PIK3CA	5.161290323	2.222222222	0.2374338	0.85080444
SETD2	5.806451613	2.777777778	0.18289269	0.85080444
TSC1	0.64516129	2.777777778	0.22241459	0.85080444
ACVR2A	2.580645161	5	0.39578696	0.87265752
APOB	9.032258065	13.66120219	0.38056823	0.87265752
ARID1B	3.870967742	1.666666667	0.31187364	0.87265752
GSE1	2.580645161	1.111111111	0.4209732	0.87265752
KEAP1	6.369426752	3.333333333	0.42618158	0.87265752
KRAS	0.64516129	2.222222222	0.37826636	0.87265752
PTEN	2.580645161	1.111111111	0.4209732	0.87265752
RAPGEF2	0.64516129	2.222222222	0.37826636	0.87265752
TLE1	0.64516129	2.762430939	0.37826636	0.87265752
ATRX	1.290322581	3.846153846	0.45744733	0.88176251
KMT2D	6.451612903	4.972375691	0.47164041	0.88176251
FRG1	4.516129032	2.777777778	0.557406	0.99868575
ALB	11.46496815	13.04347826	0.86101931	1
APC	2.580645161	3.867403315	0.75720162	1
ARID1A	8.974358974	7.73480663	0.68896113	1
ARID2	6.451612903	6.043956044	0.63925841	1
COL11A1	5.806451613	6.593406593	1	1
CRIP3	2.564102564	2.197802198	0.66565381	1
CTNNB1	28.125	29.12087912	0.62414148	1
DOCK2	7.096774194	6.629834254	0.82587195	1
IDH1	2.580645161	2.762430939	1	1
IL6ST	3.870967742	4.419889503	1	1
KMT2B	5.128205128	3.888888889	0.79124424	1
NCOR1	3.205128205	2.209944751	0.70810996	1
NFE2L2	3.225806452	3.867403315	1	1
PBRM1	1.935483871	2.222222222	1	1
RPS6KA3	5.128205128	4.444444444	1	1
SF3B1	3.225806452	2.222222222	0.73790639	1
SRCAP	3.205128205	4.395604396	0.75720162	1
TSC2	3.870967742	3.888888889	1	1
VAV3	2.580645161	3.314917127	1	1

Table S8: Proportions of individual signature across patients

Patient_ID	SBS1	SBS4	SBS5	SBS6	SBS12	SBS16	SBS22	SBS23	SBS24
TCGA-2V-A95S	0.06884551	0.08394755	0.7535272	0	0	0	0	0	0
TCGA-2Y-A9GS	0	0.26283705	0.15875992	0.24090937	0.13529064	0	0	0	0.15175082
TCGA-2Y-A9GT	0.06293926	0.09431173	0.53174189	0	0.15396987	0	0	0	0.1112014
TCGA-2Y-A9GU	0.10330835	0.1213315	0.48344681	0	0.14803499	0	0.06661407	0.07726428	0
TCGA-2Y-A9GV	0	0.15789406	0.51106262	0	0.06971816	0.11718115	0.14414401	0	0
TCGA-2Y-A9GW	0.10112499	0.07856702	0.37689565	0	0.18431157	0	0.07914458	0.10175042	0.07820578
TCGA-2Y-A9GX	0	0.11481175	0.82097358	0	0	0	0	0	0
TCGA-2Y-A9GY	0	0.16941713	0.67305343	0	0	0.08830349	0	0	0
TCGA-2Y-A9GZ	0	0.1243169	0.76365734	0	0	0	0	0	0.07209035
TCGA-2Y-A9H0	0	0	0.77944958	0	0	0	0.07907148	0	0.09401366
TCGA-2Y-A9H1	0	0.11632339	0.54869583	0	0.13151178	0	0	0.06395962	0
TCGA-2Y-A9H2	0.22041058	0.09034806	0.67157579	0	0	0	0	0	0
TCGA-2Y-A9H3	0	0.13237951	0.75330107	0	0.0670776	0	0	0	0
TCGA-2Y-A9H4	0	0.20039294	0.59029759	0	0.06740019	0	0	0.1049616	0
TCGA-2Y-A9H5	0.15169551	0	0.73259524	0	0	0	0	0	0
TCGA-2Y-A9H6	0.12399368	0	0.59461438	0	0	0.11901193	0	0	0.06819601
TCGA-2Y-A9H7	0.10920798	0.11053793	0.67840564	0	0	0	0	0	0
TCGA-2Y-A9H8	0.24598719	0.13998166	0.42073777	0	0.08542275	0	0	0.10787062	0
TCGA-2Y-A9H9	0.10622923	0.25963427	0.40490766	0	0.12790462	0.08057795	0	0	0
TCGA-2Y-A9HA	0	0.21847051	0.4168605	0	0.08473422	0.09979566	0	0	0.09427971
TCGA-2Y-A9HB	0	0.19640076	0.49983834	0.11791678	0.16885468	0	0	0	0
TCGA-3K-AAZ8	0	0	0.74314217	0	0	0	0.08382008	0	0
TCGA-4R-AA8I	0.08696461	0.06387857	0	0.44373453	0.32088467	0	0	0	0.06927415
TCGA-5C-A9VG	0.10275184	0.189892	0.60229308	0	0	0	0	0.07518087	0
TCGA-5C-A9VH	0	0.2165367	0.64127714	0	0	0	0	0	0
TCGA-5C-AAPD	0.07272181	0.258348	0.51122726	0	0.08466382	0	0	0.07303911	0
TCGA-5R-AA1C	0	0.12494558	0.66605899	0	0	0.09541764	0.11357778	0	0
TCGA-5R-AA1D	0.16045885	0	0.72678427	0	0	0	0.08364165	0	0
TCGA-5R-AAAM	0	0	0.55622139	0.12133656	0.16711436	0.06364002	0	0	0
TCGA-BC-A10R	0	0.16461762	0.71319263	0.06473447	0	0	0	0	0
TCGA-BC-A10S	0.15001405	0	0.73007824	0	0.10599982	0	0	0	0

TCGA-BC-A10T	0	0.19180797	0.61774448	0	0.06767611	0	0.07145711	0	0
TCGA-BC-A10U	0	0.20950855	0.71335906	0	0	0	0	0	0
TCGA-BC-A10W	0	0.18190255	0.7168427	0	0	0	0	0	0
TCGA-BC-A10X	0	0.35230783	0	0.07109885	0.38343255	0	0	0.14966062	0
TCGA-BC-A10Y	0	0.14230441	0.41489998	0.19062361	0.10390135	0.12462568	0	0	0
TCGA-BC-A10Z	0	0.2100818	0.62225602	0	0	0	0	0	0.10724681
TCGA-BC-A110	0.24219898	0.07488548	0.65824346	0	0	0	0	0	0
TCGA-BC-A216	0.11591656	0	0.811254	0	0	0	0	0	0
TCGA-BC-A217	0	0.2510156	0.57023703	0	0.07643377	0	0	0	0
TCGA-BC-A3KF	0	0.06085867	0.49135121	0	0.17800193	0	0.12775039	0	0.12535679
TCGA-BC-A5W4	0.11493741	0.1110383	0.60515952	0	0.07444679	0	0	0	0
TCGA-BC-A69H	0	0.18956993	0.62000824	0.09358503	0	0	0	0	0
TCGA-BC-A69I	0.13638002	0	0.71988129	0	0.13795349	0	0	0	0
TCGA-BC-A8YO	0.09332078	0.17684976	0.52436128	0	0.07259578	0	0.11937019	0	0
TCGA-BD-A2L6	0	0.19370367	0.67345349	0	0	0	0.06347347	0	0
TCGA-BD-A3EP	0	0.15274008	0.73382263	0	0	0	0	0	0
TCGA-BD-A3ER	0	0.16968312	0.67055457	0	0	0	0	0	0.11730778
TCGA-BW-A5NO	0.11039352	0.12467543	0.63449634	0	0	0	0	0.07054501	0
TCGA-BW-A5NP	0.14243996	0.16108097	0.60186973	0	0	0	0	0.09460934	0
TCGA-BW-A5NQ	0	0.15088848	0.78910493	0	0	0	0	0	0
TCGA-CC-5258	0	0	0.43947841	0.07829714	0.08313041	0.06577659	0	0	0.28155802
TCGA-CC-5259	0.06133934	0.1704692	0.51236509	0	0	0	0.24635785	0	0
TCGA-CC-5260	0.21441376	0	0.60695595	0	0	0	0.17863028	0	0
TCGA-CC-5261	0.26929244	0	0.65528229	0	0	0	0	0	0
TCGA-CC-5262	0	0.18237047	0.47886756	0	0	0.0688403	0.07874599	0	0.0793955
TCGA-CC-5263	0	0.33788708	0.32850456	0	0.08614626	0	0.15589276	0	0
TCGA-CC-5264	0	0.2131336	0.53948827	0	0.07020108	0	0.10188836	0	0
TCGA-CC-A123	0	0.32173444	0.58485754	0	0	0	0	0	0
TCGA-CC-A1HT	0	0.23563987	0.60673283	0	0	0.06061216	0	0	0
TCGA-CC-A3M9	0	0.11642241	0.48557259	0	0.09845158	0.08893487	0.11948234	0	0
TCGA-CC-A3MA	0.18669627	0.11810216	0.62505157	0	0.07015	0	0	0	0
TCGA-CC-A3MB	0.08070509	0.20957108	0.52339117	0	0	0	0	0	0.11829352
TCGA-CC-A3MC	0	0.18195357	0.37435924	0	0	0.10110079	0.09921509	0	0.14204733

TCGA-CC-A5UC	0.12497756	0.14916798	0.50829913	0	0.11123856	0	0	0.10631678	0
TCGA-CC-A5UD	0	0	0.30194758	0	0	0	0.5283109	0	0.07047918
TCGA-CC-A5UE	0	0.19031168	0.42580342	0	0	0	0	0	0.26482703
TCGA-CC-A7IE	0	0.09813293	0.41397951	0	0.09141605	0.06706224	0.10547664	0	0.16158431
TCGA-CC-A7IF	0	0.20684462	0.67776813	0	0	0	0.0798178	0	0
TCGA-CC-A7IG	0	0.14533803	0.42829565	0	0	0	0	0	0.35505794
TCGA-CC-A7IH	0	0.1733917	0.23881599	0	0.20575154	0	0.3380071	0	0
TCGA-CC-A7II	0	0.14666892	0	0	0	0.13390527	0	0	0.5402666
TCGA-CC-A7IJ	0	0.18709546	0.75399584	0	0	0	0	0	0
TCGA-CC-A7IK	0	0.16002319	0.29966053	0	0.19416925	0	0.31939074	0	0
TCGA-CC-A7IL	0	0.10866805	0.50528428	0	0	0	0	0	0.32168471
TCGA-CC-A8HS	0.11168601	0.22351139	0.41186136	0	0.12967748	0	0	0.10042477	0
TCGA-CC-A8HT	0	0.21912504	0.49699743	0	0	0	0	0	0.17981834
TCGA-CC-A8HU	0	0.20777216	0.34422249	0	0.11800822	0	0	0	0.20367689
TCGA-CC-A8HV	0	0	0.55325996	0	0	0	0.06758191	0	0.20513448
TCGA-CC-A9FS	0	0.06857412	0.56331291	0	0	0	0	0	0.27299723
TCGA-CC-A9FU	0	0.10089448	0.76744675	0	0	0	0	0	0.09543771
TCGA-CC-A9FV	0.35450019	0	0.38684191	0	0	0.25865791	0	0	0
TCGA-CC-A9FW	0	0.10144107	0.44210805	0	0.06091009	0.09352373	0.0939838	0.06660086	0.0851509
TCGA-DD-A113	0.11208802	0.1637431	0.52786097	0	0	0	0.06448078	0.08544677	0
TCGA-DD-A114	0	0.16282862	0.38566216	0	0	0	0	0	0.41247226
TCGA-DD-A115	0	0.25834951	0.55092983	0	0	0	0	0.12630728	0.06441337
TCGA-DD-A116	0	0.24891226	0.58372234	0	0.09713951	0	0	0	0
TCGA-DD-A118	0.12718171	0.07946689	0.76053011	0	0	0	0	0	0
TCGA-DD-A119	0.11076603	0.11428873	0.66605843	0	0.07773783	0	0	0	0
TCGA-DD-A11A	0.13329472	0.11520659	0.66372967	0	0	0	0	0	0
TCGA-DD-A11B	0	0.12002397	0.6424982	0	0.09651667	0	0	0.08838738	0
TCGA-DD-A11C	0.08622787	0.11833239	0.68449225	0	0	0	0	0	0
TCGA-DD-A11D	0	0.34277064	0.50057137	0	0.08100088	0	0	0	0
TCGA-DD-A1E9	0	0	0.89914007	0.06815994	0	0	0	0	0
TCGA-DD-A1EB	0	0.27569174	0.57166051	0	0	0	0	0	0
TCGA-DD-A1EC	0.23797803	0.11350293	0.33321244	0	0.22625413	0	0	0.08905247	0
TCGA-DD-A1ED	0	0.24942183	0.37202973	0	0.15044845	0.06162539	0	0.09149637	0.07497823

TCGA-DD-A1EE	0	0.23247329	0.62782368	0	0	0	0	0	0.08616202
TCGA-DD-A1EF	0	0	0.51327416	0.06974707	0.17972789	0	0.07124715	0.13171834	0
TCGA-DD-A1EH	0.19035149	0	0.55472668	0.09887129	0	0	0	0	0.07524727
TCGA-DD-A1EI	0	0.20069797	0.4892253	0.09592431	0.14385444	0	0	0	0
TCGA-DD-A1EJ	0.10437906	0.13002318	0.69016874	0	0	0	0	0	0
TCGA-DD-A1EK	0	0.19321255	0.65454809	0	0	0	0	0	0
TCGA-DD-A1EL	0.06769234	0.19836421	0	0	0.09993753	0	0	0	0.57415283
TCGA-DD-A39V	0	0.13500436	0.61377976	0.10353363	0.08354604	0	0	0	0
TCGA-DD-A39W	0.10362946	0	0.68066486	0	0	0	0	0	0.12659848
TCGA-DD-A39X	0.09565608	0	0.73910705	0	0	0	0	0	0.09838304
TCGA-DD-A39Z	0	0.08514558	0.55864643	0	0	0	0	0.17306875	0.11147819
TCGA-DD-A3A2	0	0.07201649	0.7308344	0.08211675	0	0	0	0	0.07921639
TCGA-DD-A3A3	0.10146705	0	0.70743101	0	0	0	0	0	0.12022714
TCGA-DD-A3A4	0.14167219	0.2028298	0.37825749	0.09792928	0.12678342	0	0	0	0
TCGA-DD-A3A5	0	0.12259703	0.59531635	0.15292267	0	0	0	0	0
TCGA-DD-A3A6	0	0.10298309	0.46046226	0.22657821	0.13801596	0	0	0.07196049	0
TCGA-DD-A3A7	0	0.07455084	0.504717	0	0.07283046	0.264599	0	0	0
TCGA-DD-A3A8	0	0.14983454	0.66118656	0	0.08973828	0.06134142	0	0	0
TCGA-DD-A3A9	0	0.07729456	0.62221731	0	0.15241753	0	0.06166124	0.0743273	0
TCGA-DD-A4NA	0.11729429	0.09884651	0.63938968	0	0.08596729	0	0	0	0
TCGA-DD-A4NB	0	0.13023471	0.79127471	0	0	0	0	0	0
TCGA-DD-A4ND	0	0	0.82172099	0	0.06270069	0	0	0	0
TCGA-DD-A4NE	0	0	0.75864093	0	0	0.18256294	0	0	0
TCGA-DD-A4NF	0	0.19185287	0.76498618	0	0	0	0	0	0
TCGA-DD-A4NG	0	0	0.82965619	0	0	0	0	0	0
TCGA-DD-A4NH	0	0.10107834	0.60767654	0.24255892	0	0	0	0	0
TCGA-DD-A4NI	0	0.18755229	0.58428769	0	0.13537929	0	0	0	0
TCGA-DD-A4NJ	0	0.19246365	0.67771564	0	0	0	0.0625425	0	0
TCGA-DD-A4NK	0	0.10501092	0.73910584	0	0	0	0	0	0
TCGA-DD-A4NL	0.12872537	0	0.64104185	0	0	0.11861	0	0	0
TCGA-DD-A4NN	0.11176701	0	0.66025609	0	0.09930473	0	0.08428237	0	0
TCGA-DD-A4NO	0.08903387	0.12399055	0.55517445	0	0.14976551	0	0.07466157	0	0
TCGA-DD-A4NP	0.11083196	0.09780828	0.48652342	0	0.14980082	0.06456535	0	0	0

TCGA-DD-A4NQ	0.12761593	0.11685811	0.64783971	0	0.10768625	0	0	0	0
TCGA-DD-A4NR	0	0.10734626	0.73743961	0	0.07815206	0	0	0	0
TCGA-DD-A4NS	0.13009913	0.3292342	0.34162267	0	0.13605705	0	0	0	0
TCGA-DD-A4NV	0.06811932	0.10526917	0.66632334	0	0.07108662	0	0.08920156	0	0
TCGA-DD-A73A	0	0.1857384	0.33573436	0.08208185	0.12458379	0	0.14591267	0	0.07170067
TCGA-DD-A73B	0.06333049	0.11375315	0.44406581	0.07567957	0.12657244	0.08778256	0	0	0
TCGA-DD-A73C	0	0.10605805	0.50463639	0	0	0.09561792	0.06732134	0.13157595	0
TCGA-DD-A73D	0	0.11901168	0.67739597	0	0	0	0	0	0.09623226
TCGA-DD-A73E	0	0.25039954	0.70540333	0	0	0	0	0	0
TCGA-DD-A73F	0	0.12316598	0.61531447	0.10160606	0	0	0	0	0.10462287
TCGA-DD-A73G	0	0.06531063	0.80634921	0	0	0	0	0	0
TCGA-DD-AA3A	0.20398724	0.26040478	0.51602891	0	0	0	0	0	0
TCGA-DD-AAC8	0	0	0.12536594	0	0	0	0.7862173	0	0
TCGA-DD-AAC9	0	0.22687694	0.63304517	0.06535672	0	0	0	0	0
TCGA-DD-AACB	0	0.1162781	0.60536103	0	0	0	0	0.12867773	0
TCGA-DD-AACC	0.07830048	0.23266592	0.57456768	0	0.11446592	0	0	0	0
TCGA-DD-AACD	0	0.1341619	0.50183197	0	0.12282366	0.15236793	0	0	0
TCGA-DD-AACE	0	0.12383896	0.44872476	0.14728305	0.06574486	0	0.11625208	0	0.06685405
TCGA-DD-AACF	0	0.14675754	0.62816622	0	0.06163827	0	0	0	0
TCGA-DD-AACG	0	0.22209217	0.49431653	0	0.11825813	0	0.06730268	0	0
TCGA-DD-AACH	0	0.1170617	0.70763812	0	0.07657543	0	0.06081916	0	0
TCGA-DD-AACI	0	0	0.41211899	0	0.14707934	0	0.31983626	0	0
TCGA-DD-AACJ	0.0876239	0	0.71500743	0	0.14941645	0	0	0	0
TCGA-DD-AACK	0	0	0.41863569	0	0	0	0.35960568	0	0.07791428
TCGA-DD-AACL	0	0	0.13543076	0	0	0	0.82342536	0	0
TCGA-DD-AACM	0.1775775	0.07652281	0.49708405	0	0.0983297	0	0.06303285	0.0874531	0
TCGA-DD-AACN	0.09340446	0.11573153	0.38100516	0	0.08893916	0.06644289	0.0893619	0.12468165	0
TCGA-DD-AACO	0	0.10284248	0.87343795	0	0	0	0	0	0
TCGA-DD-AACP	0.13810151	0	0.28597559	0	0.10565337	0.24925863	0.06666792	0.06911065	0.07877658
TCGA-DD-AACQ	0	0.06164854	0.26006954	0	0	0	0.67063702	0	0
TCGA-DD-AACS	0.08297189	0.06361257	0.69956347	0	0	0	0	0	0.09696877
TCGA-DD-AACT	0	0.06811881	0.36156329	0	0.11673377	0	0.40531757	0	0
TCGA-DD-AACU	0	0.12226781	0.60498544	0	0	0	0.18535897	0	0

TCGA-DD-AACV	0.06094514	0	0.39209277	0.0728194	0.15961439	0.18295231	0	0	0.08186998
TCGA-DD-AACW	0	0.26610029	0.34224304	0.22781137	0	0.0773907	0	0	0
TCGA-DD-AACX	0	0.16266337	0.63959677	0	0.09480194	0	0	0	0
TCGA-DD-AACY	0	0.122274	0.7566861	0	0	0.07135331	0	0	0
TCGA-DD-AACZ	0	0.09846794	0.28114041	0.11578438	0.07743431	0	0.31699219	0	0
TCGA-DD-AAD0	0	0.09061092	0.47142499	0.08484702	0.07587189	0	0.06122477	0	0.17732208
TCGA-DD-AAD1	0.07741854	0.17513438	0.44398711	0	0.08524778	0	0.10483262	0	0
TCGA-DD-AAD2	0	0.13313842	0.65439675	0.06955659	0	0	0.07834237	0	0
TCGA-DD-AAD3	0	0.17957607	0.53897988	0	0.06778139	0.12496093	0	0	0
TCGA-DD-AAD5	0.0609923	0.06760391	0.63748158	0	0	0.06193273	0.07144694	0	0
TCGA-DD-AAD6	0	0.0690116	0.50224713	0	0	0.24732138	0.06541366	0	0
TCGA-DD-AAD8	0	0.06465748	0.58386755	0	0.09774546	0	0.19412736	0	0
TCGA-DD-AADA	0.10712608	0.14546008	0.52223346	0	0.07628188	0	0.11723138	0	0
TCGA-DD-AADB	0	0.15018451	0.61748929	0	0.15711029	0	0	0	0
TCGA-DD-AADC	0.11144519	0	0.42973336	0	0	0.18295726	0	0	0.16993496
TCGA-DD-AADD	0	0.09900739	0.53017525	0	0	0.27091307	0	0	0
TCGA-DD-AADE	0	0	0.53579961	0	0.07434299	0.22220717	0	0	0.09851244
TCGA-DD-AADF	0	0.17115445	0.38301105	0	0	0	0.42757054	0	0
TCGA-DD-AADG	0	0.18183339	0.49543244	0	0	0.15395891	0	0	0
TCGA-DD-AADI	0	0	0.41975609	0.06478717	0.12026616	0	0.14296499	0.15885133	0.06560216
TCGA-DD-AADJ	0	0	0.61899068	0	0	0	0.07292814	0	0.1637862
TCGA-DD-AADK	0	0.1602094	0.45973304	0	0.19607367	0.0922192	0	0	0
TCGA-DD-AADL	0	0.20036205	0.44765071	0	0.07031269	0.16537201	0	0	0
TCGA-DD-AADM	0	0	0.41262856	0	0.10953392	0.30556817	0.11503811	0	0
TCGA-DD-AADN	0.06205142	0.21776958	0.28956589	0	0.13045721	0.14098573	0	0.09660351	0.06256666
TCGA-DD-AADO	0	0	0.68104779	0	0.10846399	0	0.1022862	0	0.06409471
TCGA-DD-AADP	0.09410388	0.11845646	0.51879873	0	0.14285157	0.0985354	0	0	0
TCGA-DD-AADQ	0	0.11840027	0.70863771	0	0	0	0.09678745	0	0
TCGA-DD-AADR	0	0.12323474	0.76720261	0	0	0	0	0	0
TCGA-DD-AADS	0	0.10325178	0.42486456	0	0.0938917	0	0.30959118	0	0.06840078
TCGA-DD-AADU	0	0.07932453	0.35913811	0	0.1887349	0	0.18115696	0	0.10792406
TCGA-DD-AADV	0	0	0.41316581	0	0.0627933	0.28398152	0	0.07607034	0
TCGA-DD-AADW	0.08643875	0.2310982	0.28412086	0	0.12557453	0	0.11797086	0.1547968	0

TCGA-DD-AADY	0	0.09745099	0.73339265	0.14362102	0	0	0	0	0
TCGA-DD-AAE0	0	0	0.38360151	0.29621431	0.15265045	0	0	0.08880336	0
TCGA-DD-AAE1	0.13434059	0.10203451	0.61589037	0	0.09105813	0	0	0	0
TCGA-DD-AAE2	0	0.10864127	0.75658973	0	0	0	0.1224351	0	0
TCGA-DD-AAE3	0.06223379	0	0	0	0	0.86557846	0	0	0
TCGA-DD-AAE4	0.0931741	0.26110017	0.36113551	0	0.11643042	0	0.06526506	0.10289473	0
TCGA-DD-AAE6	0	0	0.84770938	0	0	0	0	0.11064552	0
TCGA-DD-AAE8	0.09412123	0.16968613	0.55872665	0	0	0	0	0	0
TCGA-DD-AAE9	0	0.14058978	0.48408223	0	0.16333575	0	0.14306603	0	0
TCGA-DD-AAEA	0	0	0.41172093	0	0	0.41422459	0	0	0
TCGA-DD-AAEB	0	0.15999512	0.51253522	0	0	0.25154572	0	0	0
TCGA-DD-AAED	0	0.13690967	0.7623904	0	0	0	0	0	0
TCGA-DD-AAEE	0.0783311	0	0.2743613	0	0.13743781	0.18295371	0	0	0.20126692
TCGA-DD-AAEG	0.14712278	0.15036166	0.49225715	0	0.06306726	0.10436198	0	0	0
TCGA-DD-AAEH	0	0.16367415	0.47433756	0	0.14226205	0	0	0	0.16199571
TCGA-DD-AAEI	0	0.10914211	0.60877701	0	0	0	0.15955491	0	0
TCGA-DD-AAEK	0	0	0.52379622	0	0	0.25936102	0	0	0.12776815
TCGA-DD-AAVP	0	0.18174311	0.68800587	0	0.07574488	0	0	0	0
TCGA-DD-AAVQ	0	0	0.7420196	0	0	0	0	0	0.18334001
TCGA-DD-AAVR	0.12735199	0.07659862	0.39078132	0.08073742	0.06675727	0	0.0970337	0.0653083	0.09543136
TCGA-DD-AAVS	0.07946131	0.0706529	0.42744413	0	0	0.21883959	0	0	0.16681563
TCGA-DD-AAVU	0	0	0.7379547	0	0.07745764	0	0.12272272	0	0
TCGA-DD-AAVV	0	0.1710665	0.60117101	0	0.13082783	0	0	0	0
TCGA-DD-AAVX	0	0.15886211	0.51535518	0.06097144	0.11175713	0	0.06136007	0.09169408	0
TCGA-DD-AAVY	0	0.16730221	0.53961794	0	0.19825967	0	0	0	0
TCGA-DD-AAVZ	0.07269322	0.1107352	0.79745893	0	0	0	0	0	0
TCGA-DD-AAW0	0.06291071	0	0.57475633	0	0	0.08967631	0.09356689	0	0.09821923
TCGA-DD-AAW1	0	0	0.36176655	0	0	0	0.44809767	0.06034383	0
TCGA-DD-AAW2	0	0	0.46509111	0	0.07803585	0.11762922	0.1360353	0	0.10217985
TCGA-DD-AAW3	0	0.06057422	0.6988876	0	0	0	0.06331461	0	0
TCGA-ED-A459	0	0.06424448	0.31810953	0	0	0	0.58530089	0	0
TCGA-ED-A4XI	0	0	0.48636109	0	0.16346857	0.22044965	0.07742879	0	0
TCGA-ED-A5KG	0.11930616	0.07968089	0.66655492	0	0	0	0.06586976	0	0

TCGA-ED-A627	0	0	0.46931278	0	0.417108	0	0	0	0
TCGA-ED-A66X	0.26507452	0	0.64575504	0	0	0	0	0	0
TCGA-ED-A66Y	0	0	0.41357281	0.15608517	0.1831549	0	0	0.07561819	0.11984945
TCGA-ED-A7PX	0.09983183	0.18203707	0.62526882	0.09286228	0	0	0	0	0
TCGA-ED-A7PY	0.1991234	0	0.30977409	0	0.19291099	0	0	0	0.27034121
TCGA-ED-A7PZ	0	0.14187426	0.33525637	0	0	0	0.43105129	0	0
TCGA-ED-A7XO	0.08233033	0.21734423	0.29186323	0.11220175	0.16282553	0	0	0	0
TCGA-ED-A7XP	0	0.18603592	0.46097971	0.1039279	0	0	0	0	0.15924814
TCGA-ED-A82E	0.09807428	0.17272436	0.51904867	0.10663569	0	0	0	0.103517	0
TCGA-ED-A8O5	0	0.06875389	0.18458678	0.08982041	0.15631473	0	0.24741226	0.14967695	0.10343498
TCGA-ED-A8O6	0.07400358	0.16530628	0.23712029	0.25714658	0.1097008	0	0.06544666	0	0
TCGA-ED-A97K	0.11532718	0	0	0.36190407	0	0.20327606	0.13195156	0	0.10091929
TCGA-EP-A12J	0.11953289	0.25270398	0.58370649	0	0	0	0	0	0
TCGA-EP-A26S	0	0.15473273	0.74395255	0	0	0	0	0	0
TCGA-EP-A2KA	0	0.27248269	0.49144736	0.16547103	0	0	0	0	0
TCGA-EP-A2KB	0	0.26433538	0.54325125	0	0	0.12957405	0	0	0
TCGA-EP-A2KC	0.08673128	0.2128564	0.52686417	0	0.09638892	0	0	0	0
TCGA-EP-A3JL	0	0.26223424	0.56032828	0	0.16014105	0	0	0	0
TCGA-EP-A3RK	0	0.10765365	0.43766606	0.08256575	0	0.10313042	0.07878374	0.09859931	0
TCGA-ES-A2HS	0	0.17971204	0.58340101	0.10815865	0	0	0	0.07778006	0
TCGA-ES-A2HT	0	0.16443781	0.5700975	0	0	0.10389101	0	0.06710654	0
TCGA-FV-A23B	0	0.10806869	0.56882306	0.10760946	0.13605784	0	0	0.06133	0
TCGA-FV-A2QQ	0	0.22947894	0.51256162	0	0.09072486	0	0	0	0
TCGA-FV-A2QR	0	0.21380986	0.76459025	0	0	0	0	0	0
TCGA-FV-A3I0	0.0784331	0	0.73585404	0	0	0	0	0.08005433	0.10565852
TCGA-FV-A3I1	0	0.20637391	0.62890103	0	0	0	0	0	0
TCGA-FV-A3R2	0	0.23961544	0.60922338	0	0	0	0	0.06429797	0
TCGA-FV-A3R3	0.1326944	0	0.60375478	0	0.06362698	0	0	0	0.12833057
TCGA-FV-A495	0	0.11889805	0.63896143	0	0.08072319	0	0	0.06924993	0
TCGA-FV-A496	0.07651968	0.18016633	0.6753095	0	0	0	0	0	0
TCGA-FV-A4ZP	0.0829746	0.15680061	0.62802776	0	0	0	0	0.09456902	0
TCGA-FV-A4ZQ	0	0.2072569	0.42329698	0	0	0.08791637	0	0	0.16544924
TCGA-G3-A25S	0	0	0.87002071	0	0	0	0	0	0

TCGA-G3-A25T	0.12018497	0.07388051	0.71511227	0	0.09082225	0	0	0	0
TCGA-G3-A25U	0	0.52486453	0.41106395	0	0	0	0	0	0
TCGA-G3-A25V	0	0.23278149	0.38885347	0	0.10860524	0.16796546	0	0	0
TCGA-G3-A25W	0	0.16210904	0.53031776	0	0.09671101	0.13919125	0	0	0
TCGA-G3-A25Y	0	0.30055887	0.55411843	0	0	0	0	0	0.09512794
TCGA-G3-A25Z	0.07675296	0.40284706	0.23742788	0	0.14253433	0	0	0.07588029	0
TCGA-G3-A3CG	0.06876873	0.15800018	0.52069565	0	0	0.07389126	0.09295446	0	0
TCGA-G3-A3CH	0.06737785	0	0.52628449	0.13845954	0	0	0.09694791	0.07814227	0.09278794
TCGA-G3-A3CI	0	0.25186943	0.41717575	0.07343221	0.20154179	0	0	0	0
TCGA-G3-A3CK	0	0	0.70280104	0	0.17895094	0	0	0	0.06683252
TCGA-G3-A5SI	0.07103997	0.09744762	0.5481888	0	0.1338635	0	0	0	0.11003147
TCGA-G3-A5SJ	0	0.13419387	0.62543771	0	0.08787095	0.13603183	0	0	0
TCGA-G3-A5SK	0	0.21342926	0.71019378	0	0.07637697	0	0	0	0
TCGA-G3-A5SL	0	0.10428358	0.69694004	0	0	0.07690947	0	0	0
TCGA-G3-A5SM	0	0.13895373	0.64943406	0.15088194	0	0	0	0	0
TCGA-G3-A6UC	0	0.19080825	0.60451655	0	0.06957754	0	0	0	0
TCGA-G3-A7M5	0	0.07272421	0.35032434	0	0	0	0.45276394	0	0
TCGA-G3-A7M6	0	0.0720166	0.86563973	0	0.06234367	0	0	0	0
TCGA-G3-A7M7	0	0.14243657	0.79375777	0	0	0	0	0	0
TCGA-G3-A7M8	0	0	0.28935288	0.21573979	0.18569217	0.11459847	0.07759347	0	0.07014808
TCGA-G3-A7M9	0	0.28512779	0.49506971	0	0.06143314	0	0	0	0
TCGA-G3-AAUZ	0.08176302	0.25326583	0.31649046	0	0.20381784	0	0	0	0.08970328
TCGA-G3-AAV0	0	0.08783977	0.68119098	0	0	0	0.10305393	0.0662917	0
TCGA-G3-AAV1	0	0.06078076	0.65207184	0	0	0	0	0	0.13816425
TCGA-G3-AAV2	0	0	0.49964234	0.1727968	0.08025895	0	0.11753945	0	0.11654107
TCGA-G3-AAV3	0	0.17679638	0.55879143	0.10351475	0	0	0.099818	0	0
TCGA-G3-AAV4	0	0.23200766	0.35068781	0.09987402	0	0.16283341	0	0.14212049	0
TCGA-G3-AAV5	0	0.0950545	0.59379382	0.12368232	0	0	0	0	0.1199012
TCGA-G3-AAV6	0	0.21912449	0.6398106	0.10067195	0	0	0	0	0
TCGA-G3-AAV7	0	0.1417963	0.62564116	0	0	0	0	0	0.09337492
TCGA-GJ-A3OU	0.06208102	0.07095949	0.61352686	0	0.06759682	0	0	0	0.08412142
TCGA-GJ-A6C0	0	0	0.50588401	0	0	0	0	0.27792843	0.12540453
TCGA-GJ-A9DB	0	0.0656052	0.70329749	0	0	0	0	0.06034298	0.09143453

TCGA-HP-A5MZ	0	0.09325358	0.81564765	0	0	0	0	0	0
TCGA-HP-A5N0	0	0.24745895	0.50815482	0	0.13330396	0	0	0	0
TCGA-K7-A5RF	0.09180831	0	0.52323407	0	0.13041676	0.12374965	0	0	0.07675336
TCGA-K7-A5RG	0.07057744	0.09894639	0.4962278	0	0.17224688	0	0.1449706	0	0
TCGA-K7-A6G5	0.07837786	0.2211517	0.45917103	0	0	0.14806526	0	0	0
TCGA-K7-AAU7	0.06104765	0	0.83038451	0	0	0	0	0	0
TCGA-KR-A7K0	0	0.24755841	0.44895116	0	0.1040273	0	0	0.0714902	0
TCGA-KR-A7K2	0	0.17332047	0.5007188	0.06996261	0.16322225	0	0	0	0
TCGA-KR-A7K7	0.16264669	0.08964547	0.69570537	0	0	0	0	0	0
TCGA-KR-A7K8	0.11374872	0.21597835	0.47455944	0	0.16515384	0	0	0	0
TCGA-LG-A6GG	0	0.14451107	0.42170537	0	0	0	0.35985078	0	0
TCGA-LG-A9QC	0	0.09805847	0.45724043	0	0	0.15526419	0	0.07224867	0.07803279
TCGA-LG-A9QD	0	0.14408023	0.60775918	0	0.0889251	0	0	0.07871141	0
TCGA-MI-A75C	0	0.16267939	0.58868096	0	0	0.10242605	0	0	0
TCGA-MI-A75E	0	0.07974121	0.58609016	0	0.1180038	0.07031457	0.09253276	0	0
TCGA-MI-A75G	0	0.22682808	0.37542219	0	0.06600465	0.16372987	0	0	0.06888679
TCGA-MI-A75H	0	0.2121219	0.66677185	0	0.09514148	0	0	0	0
TCGA-MI-A75I	0	0.12528909	0.62871529	0	0.0679188	0	0.13257125	0	0
TCGA-MR-A520	0	0	0.61091671	0.06901191	0	0.19906937	0	0	0.09805071
TCGA-MR-A8JO	0	0	0.84505712	0	0	0.0732677	0	0	0
TCGA-NI-A4U2	0	0.08072	0.76608904	0	0	0	0	0	0
TCGA-NI-A8LF	0	0.17233431	0.73561022	0	0	0	0	0	0
TCGA-O8-A75V	0	0.19701861	0.57723748	0	0	0.11979393	0	0	0
TCGA-PD-A5DF	0.09334693	0.06977297	0.7363164	0	0	0	0.0673877	0	0
TCGA-QA-A7B7	0	0.21744197	0.55461448	0	0.10659124	0	0	0	0.06930993
TCGA-RC-A6M3	0.07752625	0	0.65105993	0	0.10896828	0.07317978	0	0	0.06614562
TCGA-RC-A6M4	0	0.21360368	0.28064727	0	0.13148078	0	0.2869433	0	0
TCGA-RC-A6M5	0.29022457	0.08506583	0.38893619	0	0.17466082	0.06111259	0	0	0
TCGA-RC-A6M6	0	0.25407973	0.53425056	0	0	0	0.07355812	0	0.09694599
TCGA-RC-A7S9	0.08617155	0.18974235	0.35179805	0	0.14677827	0.07864858	0	0.08120814	0.06565308
TCGA-RC-A7SB	0	0	0.45221334	0.07387846	0.13645878	0.09919519	0.11405267	0	0.10757174
TCGA-RC-A7SF	0	0.09165631	0.34471911	0.12010895	0.10655048	0.09606579	0.10900504	0	0.11466157
TCGA-RC-A7SH	0.12769703	0.08539329	0.52437179	0	0.13763243	0	0.0790028	0	0

TCGA-RC-A7SK	0	0.1490753	0.35606862	0	0.13546752	0.26941062	0.08997793	0	0
TCGA-RG-A7D4	0	0.12848425	0.78522627	0	0	0	0	0	0
TCGA-T1-A6J8	0	0.27630342	0.40394111	0.0913807	0	0.10744616	0	0	0.11268908
TCGA-UB-A7MA	0.14572036	0	0.80517749	0	0	0	0	0	0
TCGA-UB-A7MB	0	0.15024875	0.11961636	0	0.26370188	0	0.46643301	0	0
TCGA-UB-A7MC	0	0.09236381	0.87283277	0	0	0	0	0	0
TCGA-UB-A7MD	0.09633676	0.18370338	0.61226231	0	0	0	0	0	0
TCGA-UB-A7ME	0.0866608	0.11347405	0.4485529	0	0	0.08224966	0.26906259	0	0
TCGA-UB-A7MF	0.10013406	0.14963317	0.68086273	0	0.06008704	0	0	0	0
TCGA-UB-AA0U	0.08047357	0.10997343	0.65818909	0	0	0	0	0	0
TCGA-UB-AA0V	0.16642293	0.24554846	0.25712898	0	0.13884363	0	0	0.08998369	0.10207232
TCGA-WJ-A86L	0	0.18722758	0.67366519	0	0	0	0	0	0.08075987
TCGA-WQ-A9G7	0.10669594	0.12319277	0.10186982	0.43018528	0.2380562	0	0	0	0
TCGA-WQ-AB4B	0.06673953	0.07166547	0.80466084	0	0	0	0	0	0
TCGA-WX-AA44	0.06896674	0.14991583	0.60157227	0	0.11937491	0	0.06017024	0	0
TCGA-WX-AA46	0	0.14174031	0.67477994	0	0.11819874	0	0	0	0
TCGA-WX-AA47	0	0	0.85802466	0	0	0	0	0	0
TCGA-XR-A8TC	0.1164578	0.2476712	0.49867453	0	0	0	0	0	0
TCGA-XR-A8TD	0	0.21122674	0.73673356	0	0	0	0	0	0
TCGA-XR-A8TE	0.33036067	0.33537549	0	0	0.30136693	0	0	0	0
TCGA-XR-A8TF	0	0	0.40941996	0	0	0	0.29720217	0	0.17683653
TCGA-XR-A8TG	0	0.21674766	0.65274361	0	0.09473369	0	0	0	0
TCGA-YA-A8S7	0.09979452	0	0.61372086	0	0.15017572	0	0	0	0.07756682
TCGA-ZP-A9CV	0	0	0.73898573	0	0.11325467	0	0	0	0.06912451
TCGA-ZP-A9CY	0	0.09619573	0.48985416	0.1136893	0.16436517	0	0	0	0.13589564
TCGA-ZP-A9CZ	0.12441455	0	0.44603259	0.06622589	0.13924958	0	0.06558576	0	0.09459701
TCGA-ZP-A9D0	0.06745449	0	0.7175249	0	0	0	0.08884756	0	0.07558825
TCGA-ZP-A9D1	0	0.14205	0.33124202	0	0.27476467	0	0.20289345	0	0
TCGA-ZP-A9D2	0.07330562	0.12354847	0.44955698	0	0.2657461	0	0	0	0
TCGA-ZP-A9D4	0.08458412	0	0.50721305	0	0.11330059	0	0	0	0.19002424
TCGA-ZS-A9CD	0	0.16032072	0.6038868	0	0	0	0	0.1065871	0
TCGA-ZS-A9CE	0.08054918	0.13989916	0.72091592	0	0	0	0	0	0
TCGA-ZS-A9CF	0	0.16262114	0.64084047	0	0	0	0.0948908	0	0

TCGA-ZS-A9CG	0	0.08654847	0.70760864	0	0	0	0.10812707	0.08001576	0

Table S9: GISTIC peaks in Asian and European cohorts

Chr	Start	End	Descriptor	Type	Cohort	q.values	Gene.Symbol	Status
1	121124911	145103927	1q21.1	amp	ASIAN	8.11E-05	NA	Shared peak
1	150460562	168519364	1q22	amp	ASIAN	0.0024811	NA	Shared peak
2	340935968	341050597	2p11.1	amp	ASIAN	4.32E-09	NA	Shared peak
3	659463375	690472424	3q29	amp	ASIAN	0.14801	BDH1	Shared peak
4	729801527	731979093	4p14	amp	ASIAN	5.00E-04	NA	Private amp
5	882682663	882853016	5p15.33	amp	ASIAN	2.64E-06	TERT	Shared peak
5	1049868532	1062541960	5q35.2	amp	ASIAN	0.088559	NSD1	Shared peak
6	1062541961	1073512967	6p25.3	amp	ASIAN	0.068883	NA	Private amp
6	1095031630	1095039860	6p21.32	amp	ASIAN	0.18708	NA	Private amp
6	1121319194	1132613132	6q12	amp	ASIAN	0.081582	EYS	Shared peak
6	1121319194	1132613132	6q12	amp	ASIAN	0.081582	PHF3	Shared peak
7	1391633667	1391923977	7q36.3	amp	ASIAN	0.13082	PTPRN2	Shared peak
8	1506451443	1523546688	8q24.13	amp	ASIAN	0.016365	NA	Private amp
11	1884963012	1885469340	11q13.3	amp	ASIAN	1.76E-15	CCND1	Shared peak
11	1884963012	1885469340	11q13.3	amp	ASIAN	1.76E-15	FGF19	Shared peak
13	2180652651	2199936179	13q34	amp	ASIAN	1.35E-10	ING1	Shared peak
14	2199936180	2219496035	14q11.2	amp	ASIAN	0.029451	CHD8	Private amp
14	2199936180	2219496035	14q11.2	amp	ASIAN	0.029451	PRMT5	Private amp
16	2443236820	2457303927	16q11.2	amp	ASIAN	4.53E-15	NA	Shared peak
17	2520852936	2520970982	17p11.2	amp	ASIAN	0.015886	NA	Private amp
17	2579327271	2581367074	17q25.3	amp	ASIAN	0.052112	CBX8	Private amp
17	2579327271	2581367074	17q25.3	amp	ASIAN	0.052112	FOXK2	Private amp
18	2658746978	2658824809	18q23	amp	ASIAN	0.12232	NA	Private amp
19	2673729311	2674829948	19p13.12	amp	ASIAN	0.045401	NOTCH3	Private amp
19	2673729311	2674829948	19p13.12	amp	ASIAN	0.045401	BRD4	Private amp
19	2677937514	2681264096	19p13.11	amp	ASIAN	0.071994	NA	Private amp
19	2699132751	2701151724	19q13.2	amp	ASIAN	0.049956	PAF1	Private amp
1	1	8417570	1p36.31	del	ASIAN	2.71E-17	RPL22	Shared peak
2	304123567	439564226	2q31.2	del	ASIAN	0.20089	NFE2L2	Shared peak
2	484020028	492449994	2q37.3	del	ASIAN	0.0077107	ING5	Shared peak
4	748348736	800206972	4q13.3	del	ASIAN	0.017161	COX18	Shared peak
4	748348736	800206972	4q13.3	del	ASIAN	0.017161	ALB	Shared peak
4	861470534	881626700	4q35.1	del	ASIAN	1.29E-12	NA	Shared peak
6	1231852223	1233657027	6q27	del	ASIAN	2.39E-06	PHF10	Shared peak
8	1399548263	1400512832	8p23.1	del	ASIAN	0.017161	NA	Shared peak
9	1558926635	1566328026	9p21.3	del	ASIAN	3.19E-04	CDKN2A	Shared peak
9	1558926635	1566328026	9p21.3	del	ASIAN	3.19E-04	CDKN2B	Shared peak
9	1583334731	1605659606	9p11.2	del	ASIAN	0.13654	NA	Shared peak
11	1881292846	1881945346	11q13.1	del	ASIAN	0.21741	NA	Private del
11	1815907891	1950914406	11q22.3	del	ASIAN	0.18936	ATM	Shared peak
12	2071832920	2084766301	12q24.32	del	ASIAN	0.061234	NA	Shared peak
13	2084766302	2126791164	13q12.11	del	ASIAN	0.024817	MPHOSPH8	Private del
13	2084766302	2126791164	13q12.11	del	ASIAN	0.024817	XPO4	Private del
13	2129229282	2136253547	13q14.2	del	ASIAN	8.46E-11	RB1	Shared peak
14	2306067962	2306727184	14q32.33	del	ASIAN	0.0023367	TDRD9	Shared peak
14	2306067962	2306727184	14q32.33	del	ASIAN	0.0023367	AKT1	Shared peak
15	2329403992	2329832159	15q11.2	del	ASIAN	4.89E-05	NA	Private del
15	2393618570	2409817111	15q26.3	del	ASIAN	0.011676	IGF1R	Shared peak
16	2409817112	2410241164	16p13.3	del	ASIAN	0.0054228	AXIN1	Shared peak
16	2409817112	2410241164	16p13.3	del	ASIAN	0.0054228	TSC2	Shared peak

16	2409817112	2410241164	16p13.3	del	ASIAN	0.0054228	RBFOX1	Shared peak
16	2442831013	2457303927	16q11.2	del	ASIAN	1.25E-23	NA	Shared peak
16	2487058908	2500171864	16q24.3	del	ASIAN	0.010288	NA	Private del
22	2829728721	2860214161	22q11.1	del	ASIAN	0.091645	NA	Shared peak
1	16731648	16902893	1p36.13	amp	EUROPEAN	0.017814	NA	Private amp
1	121124363	145075207	1q21.1	amp	EUROPEAN	3.21E-06	NA	Shared peak
1	149726033	151740447	1q21.3	amp	EUROPEAN	1.06E-08	MCL1	Shared peak
1	149726033	151740447	1q21.3	amp	EUROPEAN	1.06E-08	ARNT	Shared peak
1	149726033	151740447	1q21.3	amp	EUROPEAN	1.06E-08	KCNN3	Shared peak
1	248722452	249250621	1q44	amp	EUROPEAN	4.11E-04	SMYD3	Private amp
2	249250622	270144360	2p24.2	amp	EUROPEAN	0.056761	NA	Private amp
2	340928288	341059847	2p11.1	amp	EUROPEAN	3.20E-05	NA	Shared peak
3	663882472	666457430	3q26.31	amp	EUROPEAN	0.10402	NA	Shared peak
4	760260678	760473436	4q13.2	amp	EUROPEAN	0.15697	NA	Private amp
5	882720562	882956952	5p15.33	amp	EUROPEAN	0.0043469	TERT	Shared peak
5	1060689243	1062541960	5q35.3	amp	EUROPEAN	0.10793	PRELID1	Shared peak
6	1095092059	1095151085	6p21.32	amp	EUROPEAN	0.0041401	NA	Private amp
6	1106141067	1106558970	6p21.1	amp	EUROPEAN	4.85E-04	CRIP3	Private amp
6	1120014494	1127567805	6p11.1	amp	EUROPEAN	9.46E-05	NA	Shared peak
7	1349797552	1354636745	7q31.2	amp	EUROPEAN	0.0010993	MET	Private amp
7	1391054539	1391991961	7q36.3	amp	EUROPEAN	0.12502	PTPRN2	Shared peak
8	1478227557	1479864810	8q21.2	amp	EUROPEAN	0.070871	NA	Private amp
8	1535298124	1536218523	8q24.3	amp	EUROPEAN	0.033342	PARP10	Private amp
8	1535298124	1536218523	8q24.3	amp	EUROPEAN	0.033342	CYC1	Private amp
9	1678428165	1678807797	9q34.3	amp	EUROPEAN	0.23077	NOTCH1	Private amp
11	1884646820	1885533400	11q13.3	amp	EUROPEAN	4.54E-09	CCND1	Shared peak
11	1884646820	1885533400	11q13.3	amp	EUROPEAN	4.54E-09	FGF19	Shared peak
12	1951579982	1953482287	12p13.33	amp	EUROPEAN	0.22316	KDM5A	Private amp
12	2008914416	2010204813	12q14.1	amp	EUROPEAN	0.056761	CDK4	Private amp
13	2181296653	2199936179	13q34	amp	EUROPEAN	1.85E-05	ING1	Shared peak
15	2407179476	2409817111	15q26.3	amp	EUROPEAN	0.024383	IGF1R	Private amp
16	2443242029	2462795260	16q11.2	amp	EUROPEAN	6.36E-34	NA	Shared peak
17	2521492661	2526153585	17p11.2	amp	EUROPEAN	0.070871	NA	Private amp
17	2573089279	2573386206	17q25.1	amp	EUROPEAN	1.17E-04	NA	Private amp
19	2668448072	2668532651	19p13.2	amp	EUROPEAN	0.010132	MRPL4	Private amp
19	2668448072	2668532651	19p13.2	amp	EUROPEAN	0.010132	KEAP1	Private amp
20	2718573306	2781598825	20q13.2	amp	EUROPEAN	0.057958	ZNF217	Private amp
20	2768971655	2772985203	20q13.2	amp	EUROPEAN	0.057958	ZNF217	Private amp
22	2867215496	2868962806	22q13.1	amp	EUROPEAN	0.099588	TNRC6B	Private amp
1	2309793	6654351	1p36.31	del	EUROPEAN	1.86E-19	RPL22	Shared peak
1	16950405	17007888	1p36.13	del	EUROPEAN	6.96E-18	NA	Private del
2	388572751	407369654	2q22.1	del	EUROPEAN	3.50E-04	LRP1B	Shared peak
2	482970449	492449994	2q37.3	del	EUROPEAN	0.010525	ING5	Shared peak
3	538388356	558883326	3p21.1	del	EUROPEAN	0.0017145	BAP1	Private del
3	538388356	558883326	3p21.1	del	EUROPEAN	0.0017145	PBRM1	Private del
3	539179193	590113645	3p13	del	EUROPEAN	0.0093281	NA	Private del
3	686762822	690472424	3q29	del	EUROPEAN	0.017818	BDH1	Private del
4	693948060	694251946	4p16.3	del	EUROPEAN	0.045018	FGFR3	Private del
4	693948060	694251946	4p16.3	del	EUROPEAN	0.045018	LETM1	Private del
4	753336394	804211969	4q21.23	del	EUROPEAN	9.84E-07	NA	Shared peak
4	778481941	779215159	4q22.1	del	EUROPEAN	8.22E-08	FAM190A	Shared peak
4	764333152	800833263	4q23	del	EUROPEAN	5.10E-05	ADH1B	Shared peak

4	868750395	881626700	4q35.2	del	EUROPEAN	4.85E-06	FAT1	Shared peak
4	868750395	881626700	4q35.2	del	EUROPEAN	4.85E-06	FRG1	Shared peak
5	946616656	1019146832	5q14.3	del	EUROPEAN	0.175	NA	Private del
6	1143291549	1173288466	6q16.1	del	EUROPEAN	0.038251	NA	Private del
6	1231253131	1233657027	6q27	del	EUROPEAN	0.0051302	PHF10	Shared peak
7	1233657028	1240775271	7p22.3	del	EUROPEAN	0.18864	NA	Private del
7	1384891620	1392795690	7q36.3	del	EUROPEAN	0.12689	PTPRN2	Private del
8	1394886875	1400090031	8p23.1	del	EUROPEAN	0.0053602	NA	Shared peak
9	1539159713	1544330370	9p24.2	del	EUROPEAN	0.24834	NA	Private del
9	1559780288	1566276510	9p21.2	del	EUROPEAN	0.026514	NA	Shared peak
9	1579913496	1605659481	9p12	del	EUROPEAN	0.0018691	NA	Shared peak
9	1665019423	1680373143	9q34.3	del	EUROPEAN	0.013722	NOTCH1	Private del
10	1798074294	1814293217	10q26.11	del	EUROPEAN	2.39E-04	NA	Private del
11	1815907891	1816555338	11p15.5	del	EUROPEAN	4.84E-04	SIRT3	Shared peak
11	1815907891	1816555338	11p15.5	del	EUROPEAN	4.84E-04	PHRF1	Shared peak
11	1918863697	1950914406	11q25	del	EUROPEAN	0.17979	NA	Shared peak
12	1950914407	1965689665	12p13.31	del	EUROPEAN	0.13301	NA	Private del
12	2072924778	2084766301	12q24.33	del	EUROPEAN	0.030845	NA	Shared peak
13	2131445284	2145007261	13q14.2	del	EUROPEAN	8.96E-09	RB1	Shared peak
13	2155081109	2160627679	13q22.2	del	EUROPEAN	1.74E-06	NA	Private del
14	2305855341	2307285719	14q32.33	del	EUROPEAN	2.60E-08	TDRD9	Shared peak
14	2305855341	2307285719	14q32.33	del	EUROPEAN	2.60E-08	AKT1	Shared peak
15	2392475184	2409817111	15q26.1	del	EUROPEAN	0.18013	NA	Shared peak
16	2409817112	2410255666	16p13.3	del	EUROPEAN	3.07E-04	AXIN1	Shared peak
16	2409817112	2410255666	16p13.3	del	EUROPEAN	3.07E-04	TSC2	Shared peak
16	2409817112	2410255666	16p13.3	del	EUROPEAN	3.07E-04	RBFOX1	Shared peak
16	2442288777	2462786552	16q11.2	del	EUROPEAN	6.91E-22	NA	Shared peak
17	2520620148	2526259755	17p11.2	del	EUROPEAN	0.075925	NA	Private del
19	2659444323	2662925142	19p13.3	del	EUROPEAN	0.034126	STK11	Private del
21	2811969411	2814638523	21q21.3	del	EUROPEAN	0.06626	NA	Private del
21	2819383192	2829728720	21q22.3	del	EUROPEAN	0.21707	PTTG1IP	Private del
22	2829728721	2846178503	22q11.1	del	EUROPEAN	0.034387	NA	Shared peak
22	2880413370	2881033286	22q13.33	del	EUROPEAN	9.45E-04	BRD1	Private del
22	2880413370	2881033286	22q13.33	del	EUROPEAN	9.45E-04	HDAC10	Private del

Table S10: Correlation p-value of each feature pair across TCGA, Asian and European cohorts

Feature 1	Feature 2	p-value	fdr	Cohort
Age	pLMC	0.059174313	0.153414886	TCGA
Age	race	1.52E-10	3.54E-09	TCGA
Age	pLMC	0.092070849	0.312383236	Asian
Age	pLMC	0.033197698	0.157044702	European
AMP_5p	ARID2	0.072786516	0.184158655	TCGA
AMP_5p	grade	0.885876141	0.973999945	TCGA
AMP_5p	Shannon	0.200673654	0.41315164	TCGA
AMP_5p	SBS22	0.11385832	0.254364333	TCGA
AMP_5p	TMB	0.029824207	0.097860679	TCGA
AMP_5p	DOCK2	1	1	TCGA
AMP_5p	HBV	0.012490644	0.048574726	TCGA
AMP_5p	MDSC	0.006627813	0.028996684	TCGA
AMP_5p	SCNA	3.34E-07	4.38E-06	TCGA
AMP_5p	Stage	0.333382475	0.560082558	TCGA
AMP_5p	ARID1A	0.095309036	0.222833524	TCGA
AMP_5p	RNAGR	0.078497696	0.193355187	TCGA
AMP_5p	Purity	0.927338604	0.993577075	TCGA
AMP_5p	DEL_4q	0.079183553	0.193355187	TCGA
AMP_5p	APOB	1	1	TCGA
AMP_5p	Age	0.903409886	0.98370096	TCGA
AMP_5p	pLMC	0.021362899	0.073377643	TCGA
AMP_5p	race	1	1	TCGA
AMP_5p	ARID2	0.315829691	0.697763271	Asian
AMP_5p	grade	0.79558803	0.998852414	Asian
AMP_5p	Shannon	0.053744685	0.208397756	Asian
AMP_5p	SBS22	0.055950621	0.21261236	Asian
AMP_5p	TMB	0.56873869	0.834180629	Asian
AMP_5p	DOCK2	0.344930602	0.712231422	Asian
AMP_5p	HBV	0.001988622	0.01889191	Asian
AMP_5p	MDSC	0.015351255	0.074788165	Asian
AMP_5p	SCNA	6.07E-04	0.006406768	Asian
AMP_5p	Stage	0.239386338	0.590693561	Asian
AMP_5p	ARID1A	0.195849905	0.518662929	Asian
AMP_5p	RNAGR	0.359864297	0.712231422	Asian
AMP_5p	Purity	0.886675269	1	Asian
AMP_5p	DEL_4q	0.049667726	0.200018638	Asian
AMP_5p	APOB	1	1	Asian
AMP_5p	Age	0.805624085	0.998852414	Asian
AMP_5p	pLMC	0.409406683	0.753706474	Asian
AMP_5p	ARID2	0.148555064	0.409064669	European
AMP_5p	grade	0.767582572	0.925028382	European
AMP_5p	Shannon	0.945809368	0.996687335	European
AMP_5p	SBS22	0.943921748	0.996687335	European
AMP_5p	TMB	0.016596667	0.092746081	European
AMP_5p	DOCK2	0.403571158	0.737293462	European
AMP_5p	HBV	0.739966853	0.924958566	European
AMP_5p	MDSC	0.101818391	0.31713925	European
AMP_5p	SCNA	1.23E-04	0.001804124	European

AMP_5p	Stage	0.948089224	0.996687335	European
AMP_5p	ARID1A	0.313010182	0.631685223	European
AMP_5p	RNAGR	0.062307046	0.236766776	European
AMP_5p	Purity	0.95475772	0.996725092	European
AMP_5p	DEL_4q	0.574224969	0.865894794	European
AMP_5p	APOB	0.764719727	0.925028382	European
AMP_5p	Age	0.885457639	0.980190904	European
AMP_5p	pLMC	0.011536693	0.07306572	European
APOB	Age	0.041167263	0.127134195	TCGA
APOB	pLMC	0.091111366	0.21742485	TCGA
APOB	race	0.682223591	0.864365633	TCGA
APOB	Age	0.249313965	0.599615865	Asian
APOB	pLMC	0.344258619	0.712231422	Asian
APOB	Age	0.023544266	0.120902985	European
APOB	pLMC	0.211084318	0.501325254	European
ARID1A	RNAGR	0.985317845	1	TCGA
ARID1A	Purity	0.423684299	0.649443086	TCGA
ARID1A	DEL_4q	0.475061723	0.711947474	TCGA
ARID1A	APOB	0.407008571	0.634251265	TCGA
ARID1A	Age	0.775283968	0.919828436	TCGA
ARID1A	pLMC	0.689767834	0.864365633	TCGA
ARID1A	race	0.81465576	0.924533162	TCGA
ARID1A	RNAGR	0.912025737	1	Asian
ARID1A	Purity	0.814424387	0.998852414	Asian
ARID1A	DEL_4q	0.514823503	0.794750507	Asian
ARID1A	APOB	1	1	Asian
ARID1A	Age	0.632490038	0.88645336	Asian
ARID1A	pLMC	0.827753036	1	Asian
ARID1A	RNAGR	0.474911339	0.79852349	European
ARID1A	Purity	0.186904118	0.455279263	European
ARID1A	DEL_4q	1	1	European
ARID1A	APOB	0.177053025	0.445556101	European
ARID1A	Age	0.338185126	0.662424473	European
ARID1A	pLMC	0.736636316	0.924958566	European
ARID2	grade	0.691492507	0.864365633	TCGA
ARID2	Shannon	0.332061983	0.560082558	TCGA
ARID2	SBS22	0.993846093	1	TCGA
ARID2	TMB	0.331955061	0.560082558	TCGA
ARID2	DOCK2	0.015311548	0.055860754	TCGA
ARID2	HBV	0.595364803	0.80145262	TCGA
ARID2	MDSC	0.795437482	0.924533162	TCGA
ARID2	SCNA	0.662606498	0.853664814	TCGA
ARID2	Stage	0.756706948	0.902888973	TCGA
ARID2	ARID1A	0.6176488	0.81459206	TCGA
ARID2	RNAGR	0.342957108	0.563082082	TCGA
ARID2	Purity	0.913436606	0.98370096	TCGA
ARID2	DEL_4q	1	1	TCGA
ARID2	APOB	0.671326802	0.859625783	TCGA
ARID2	Age	0.505942464	0.73783276	TCGA
ARID2	pLMC	0.167811944	0.359597022	TCGA

ARID2	race	0.805620701	0.924533162	TCGA
ARID2	grade	0.843509498	1	Asian
ARID2	Shannon	0.459128146	0.778878106	Asian
ARID2	SBS22	0.374165827	0.723142858	Asian
ARID2	TMB	0.296640089	0.679055625	Asian
ARID2	DOCK2	0.027177341	0.120085927	Asian
ARID2	HBV	1	1	Asian
ARID2	MDSC	0.939197656	1	Asian
ARID2	SCNA	0.907292023	1	Asian
ARID2	Stage	0.813878425	0.998852414	Asian
ARID2	ARID1A	1	1	Asian
ARID2	RNAGR	0.774185703	0.980635224	Asian
ARID2	Purity	0.581576814	0.843508357	Asian
ARID2	DEL_4q	1	1	Asian
ARID2	APOB	1	1	Asian
ARID2	Age	0.530285084	0.795908825	Asian
ARID2	pLMC	0.871902184	1	Asian
ARID2	grade	0.356948854	0.683439709	European
ARID2	Shannon	0.62740634	0.899648937	European
ARID2	SBS22	0.164861252	0.430733004	European
ARID2	TMB	0.732556439	0.924958566	European
ARID2	DOCK2	0.305937562	0.631685223	European
ARID2	HBV	0.461119771	0.789304113	European
ARID2	MDSC	0.812486009	0.964827136	European
ARID2	SCNA	0.651985341	0.917608999	European
ARID2	Stage	0.50392139	0.818333882	European
ARID2	ARID1A	1	1	European
ARID2	RNAGR	0.219843624	0.515682575	European
ARID2	Purity	0.559689692	0.864561313	European
ARID2	DEL_4q	1	1	European
ARID2	APOB	0.526623421	0.842188639	European
ARID2	Age	0.791486484	0.945801459	European
ARID2	pLMC	0.049592124	0.196302156	European
DEL_16q	GEP	0.05065576	0.143888737	TCGA
DEL_16q	AMP_5p	0.534757145	0.751106402	TCGA
DEL_16q	ARID2	0.62064157	0.81459206	TCGA
DEL_16q	grade	0.047548168	0.140635427	TCGA
DEL_16q	Shannon	0.839931005	0.933256672	TCGA
DEL_16q	SBS22	0.856353241	0.946495687	TCGA
DEL_16q	TMB	0.781953764	0.922529721	TCGA
DEL_16q	DOCK2	0.321034134	0.557166678	TCGA
DEL_16q	HBV	0.300677693	0.539677911	TCGA
DEL_16q	MDSC	2.65E-05	1.86E-04	TCGA
DEL_16q	SCNA	7.17E-07	7.92E-06	TCGA
DEL_16q	Stage	0.264542172	0.482994196	TCGA
DEL_16q	ARID1A	0.343211936	0.563082082	TCGA
DEL_16q	RNAGR	1.75E-12	5.25E-11	TCGA
DEL_16q	Purity	0.479190247	0.711947474	TCGA
DEL_16q	DEL_4q	1.17E-07	1.75E-06	TCGA
DEL_16q	APOB	0.003664109	0.016727453	TCGA

DEL_16q	Age	0.008967491	0.037663463	TCGA
DEL_16q	pLMC	0.044145735	0.134356584	TCGA
DEL_16q	race	0.068304913	0.174927216	TCGA
DEL_16q	GEP	0.136075049	0.417004181	Asian
DEL_16q	AMP_5p	0.478540776	0.784573612	Asian
DEL_16q	ARID2	1	1	Asian
DEL_16q	grade	0.918198526	1	Asian
DEL_16q	Shannon	0.840906353	1	Asian
DEL_16q	SBS22	0.43938432	0.765899274	Asian
DEL_16q	TMB	0.138430433	0.417488608	Asian
DEL_16q	DOCK2	0.534435875	0.795908825	Asian
DEL_16q	HBV	0.600847752	0.858353931	Asian
DEL_16q	MDSC	0.024664454	0.11157729	Asian
DEL_16q	SCNA	0.207335059	0.532346774	Asian
DEL_16q	Stage	0.195133979	0.518662929	Asian
DEL_16q	ARID1A	0.328241973	0.70074129	Asian
DEL_16q	RNAGR	1.73E-08	4.70E-07	Asian
DEL_16q	Purity	0.645894842	0.88645336	Asian
DEL_16q	DEL_4q	5.61E-05	8.89E-04	Asian
DEL_16q	APOB	0.003688261	0.028030786	Asian
DEL_16q	Age	0.004181116	0.029422667	Asian
DEL_16q	pLMC	0.485175557	0.784573612	Asian
DEL_16q	GEP	0.27075468	0.591303325	European
DEL_16q	AMP_5p	0.078391861	0.261306203	European
DEL_16q	ARID2	0.714918484	0.924958566	European
DEL_16q	grade	0.009839888	0.064468235	European
DEL_16q	Shannon	0.694872238	0.924958566	European
DEL_16q	SBS22	0.731446928	0.924958566	European
DEL_16q	TMB	0.127417587	0.374367518	European
DEL_16q	DOCK2	0.686506056	0.924958566	European
DEL_16q	HBV	0.315842612	0.631685223	European
DEL_16q	MDSC	0.00109381	0.010391195	European
DEL_16q	SCNA	1.22E-07	5.81E-06	European
DEL_16q	Stage	0.542459178	0.851795404	European
DEL_16q	ARID1A	1	1	European
DEL_16q	RNAGR	3.00E-04	0.003355061	European
DEL_16q	Purity	0.266837609	0.590610554	European
DEL_16q	DEL_4q	0.004413236	0.03225057	European
DEL_16q	APOB	0.35970511	0.683439709	European
DEL_16q	Age	0.480798177	0.801330295	European
DEL_16q	pLMC	7.91E-04	0.008344684	European
DEL_4q	APOB	0.407732956	0.634251265	TCGA
DEL_4q	Age	0.001092594	0.005306399	TCGA
DEL_4q	pLMC	0.001337794	0.006243041	TCGA
DEL_4q	race	1.01E-05	7.83E-05	TCGA
DEL_4q	APOB	0.096621855	0.322072851	Asian
DEL_4q	Age	0.029114543	0.125721892	Asian
DEL_4q	pLMC	0.038569979	0.162851022	Asian
DEL_4q	APOB	0.755518997	0.925028382	European
DEL_4q	Age	0.690492523	0.924958566	European

DEL_4q	pLMC	0.042448806	0.176218766	European
DOCK2	HBV	0.595364803	0.80145262	TCGA
DOCK2	MDSC	0.317205373	0.555109402	TCGA
DOCK2	SCNA	0.714368705	0.872194349	TCGA
DOCK2	Stage	0.60829787	0.813646832	TCGA
DOCK2	ARID1A	0.6176488	0.81459206	TCGA
DOCK2	RNAGR	0.110125268	0.248669959	TCGA
DOCK2	Purity	0.960475719	1	TCGA
DOCK2	DEL_4q	1	1	TCGA
DOCK2	APOB	0.215238586	0.431736514	TCGA
DOCK2	Age	0.713050962	0.872194349	TCGA
DOCK2	pLMC	0.022192278	0.073974261	TCGA
DOCK2	race	0.215868257	0.431736514	TCGA
DOCK2	HBV	0.344930602	0.712231422	Asian
DOCK2	MDSC	0.376795489	0.723142858	Asian
DOCK2	SCNA	0.831799141	1	Asian
DOCK2	Stage	0.404380676	0.753258121	Asian
DOCK2	ARID1A	1	1	Asian
DOCK2	RNAGR	0.310207975	0.694107744	Asian
DOCK2	Purity	0.353689558	0.712231422	Asian
DOCK2	DEL_4q	1	1	Asian
DOCK2	APOB	0.325794309	0.70074129	Asian
DOCK2	Age	0.276878984	0.657587586	Asian
DOCK2	pLMC	0.428137163	0.760243561	Asian
DOCK2	HBV	0.060842883	0.235921384	European
DOCK2	MDSC	0.929185869	0.996687335	European
DOCK2	SCNA	0.908565541	0.992111798	European
DOCK2	Stage	0.1786844	0.445556101	European
DOCK2	ARID1A	1	1	European
DOCK2	RNAGR	0.874451082	0.980190904	European
DOCK2	Purity	0.527476042	0.842188639	European
DOCK2	DEL_4q	0.667109595	0.924958566	European
DOCK2	APOB	0.426776765	0.7578279	European
DOCK2	Age	0.859039695	0.980190904	European
DOCK2	pLMC	0.0177861	0.096553112	European
GEP	AMP_5p	0.22392717	0.435413942	TCGA
GEP	ARID2	0.219661715	0.435178868	TCGA
GEP	grade	0.560658557	0.774594059	TCGA
GEP	Shannon	0.594909913	0.80145262	TCGA
GEP	SBS22	0.22271748	0.435413942	TCGA
GEP	TMB	0.152441696	0.33346621	TCGA
GEP	DOCK2	0.827930087	0.926169434	TCGA
GEP	HBV	0.008837469	0.037663463	TCGA
GEP	MDSC	7.80E-19	5.46E-17	TCGA
GEP	SCNA	8.02E-05	5.10E-04	TCGA
GEP	Stage	0.103252731	0.235685582	TCGA
GEP	ARID1A	0.209966261	0.428086551	TCGA
GEP	RNAGR	0.045130028	0.135390084	TCGA
GEP	Purity	3.33E-15	1.16E-13	TCGA
GEP	DEL_4q	0.077061042	0.192652606	TCGA

GEP	APOB	0.529364502	0.751106402	TCGA
GEP	Age	0.68465296	0.864365633	TCGA
GEP	pLMC	4.60E-04	0.002474921	TCGA
GEP	race	0.510946167	0.739991	TCGA
GEP	AMP_5p	0.078379361	0.275779235	Asian
GEP	ARID2	0.156966509	0.451873283	Asian
GEP	grade	0.160083192	0.453967261	Asian
GEP	Shannon	0.008663908	0.049883105	Asian
GEP	SBS22	0.354699593	0.712231422	Asian
GEP	TMB	0.484862096	0.784573612	Asian
GEP	DOCK2	0.676961973	0.905794189	Asian
GEP	HBV	2.49E-04	0.002788193	Asian
GEP	MDSC	1.71E-11	1.08E-09	Asian
GEP	SCNA	0.002343086	0.019612461	Asian
GEP	Stage	0.015067188	0.074788165	Asian
GEP	ARID1A	0.533359478	0.795908825	Asian
GEP	RNAGR	0.009883244	0.055229891	Asian
GEP	Purity	2.74E-11	1.30E-09	Asian
GEP	DEL_4q	0.233563662	0.583909154	Asian
GEP	APOB	0.724899467	0.949868267	Asian
GEP	Age	0.293947166	0.679055625	Asian
GEP	pLMC	0.013249208	0.068036476	Asian
GEP	AMP_5p	0.98607726	1	European
GEP	ARID2	0.736035944	0.924958566	European
GEP	grade	0.468544829	0.794852834	European
GEP	Shannon	0.130043454	0.374367518	European
GEP	SBS22	0.489417891	0.801632752	European
GEP	TMB	0.194620192	0.468073878	European
GEP	DOCK2	0.358219763	0.683439709	European
GEP	HBV	0.274714582	0.593133757	European
GEP	MDSC	2.23E-09	2.12E-07	European
GEP	SCNA	0.012315775	0.07548378	European
GEP	Stage	0.949475829	0.996687335	European
GEP	ARID1A	0.283008691	0.604175857	European
GEP	RNAGR	0.413374346	0.742460115	European
GEP	Purity	2.92E-06	7.66E-05	European
GEP	DEL_4q	0.236185134	0.540664765	European
GEP	APOB	0.591616493	0.878277788	European
GEP	Age	0.568604994	0.865894794	European
GEP	pLMC	0.01526891	0.090659156	European
grade	Shannon	0.432255994	0.65778086	TCGA
grade	SBS22	0.978043374	1	TCGA
grade	TMB	0.191538167	0.398247673	TCGA
grade	DOCK2	0.657395953	0.853664814	TCGA
grade	HBV	7.04E-07	7.92E-06	TCGA
grade	MDSC	4.31E-04	0.002447443	TCGA
grade	SCNA	7.25E-04	0.003681903	TCGA
grade	Stage	0.310313847	0.547612672	TCGA
grade	ARID1A	0.520369928	0.748477293	TCGA
grade	RNAGR	4.08E-04	0.00237907	TCGA

grade	Purity	0.968980642	1	TCGA
grade	DEL_4q	0.009740545	0.039497889	TCGA
grade	APOB	0.447702619	0.676385251	TCGA
grade	Age	0.04854202	0.141580891	TCGA
grade	pLMC	0.536504573	0.751106402	TCGA
grade	race	5.15E-06	4.51E-05	TCGA
grade	Shannon	0.734536867	0.955055652	Asian
grade	SBS22	0.48161385	0.784573612	Asian
grade	TMB	0.506141296	0.794750507	Asian
grade	DOCK2	0.765508441	0.976151704	Asian
grade	HBV	0.002717771	0.021515689	Asian
grade	MDSC	0.310521885	0.694107744	Asian
grade	SCNA	0.184407556	0.515256406	Asian
grade	Stage	0.098332944	0.322125161	Asian
grade	ARID1A	0.907980956	1	Asian
grade	RNAGR	0.936397134	1	Asian
grade	Purity	0.706882992	0.932692837	Asian
grade	DEL_4q	0.570755167	0.834180629	Asian
grade	APOB	0.358229231	0.712231422	Asian
grade	Age	0.457205262	0.778878106	Asian
grade	pLMC	0.425819979	0.760243561	Asian
grade	Shannon	0.629754256	0.899648937	European
grade	SBS22	0.53899478	0.851795404	European
grade	TMB	0.455708335	0.787132578	European
grade	DOCK2	0.855731192	0.980190904	European
grade	HBV	0.435958383	0.763075229	European
grade	MDSC	0.003685141	0.028007072	European
grade	SCNA	0.004795106	0.033743342	European
grade	Stage	0.155748066	0.42274475	European
grade	ARID1A	0.136330504	0.386608891	European
grade	RNAGR	5.25E-05	9.07E-04	European
grade	Purity	0.595340911	0.878277788	European
grade	DEL_4q	0.162940861	0.430733004	European
grade	APOB	0.399917579	0.737293462	European
grade	Age	0.180567473	0.445556101	European
grade	pLMC	0.171132789	0.439394998	European
HBV	MDSC	0.732400017	0.882204919	TCGA
HBV	SCNA	0.484802328	0.711947474	TCGA
HBV	Stage	1.87E-05	1.35E-04	TCGA
HBV	ARID1A	0.801702811	0.924533162	TCGA
HBV	RNAGR	0.084542068	0.204067062	TCGA
HBV	Purity	0.158822221	0.343841923	TCGA
HBV	DEL_4q	0.001111817	0.005306399	TCGA
HBV	APOB	0.661823146	0.853664814	TCGA
HBV	Age	1.35E-05	1.01E-04	TCGA
HBV	pLMC	0.527422076	0.751106402	TCGA
HBV	race	8.83E-22	9.27E-20	TCGA
HBV	MDSC	7.51E-05	0.001098152	Asian
HBV	SCNA	0.371381707	0.723142858	Asian
HBV	Stage	4.18E-10	1.32E-08	Asian

HBV	ARID1A	0.518679278	0.794750507	Asian
HBV	RNAGR	0.070677333	0.25337157	Asian
HBV	Purity	0.738911478	0.955055652	Asian
HBV	DEL_4q	0.593706733	0.854577874	Asian
HBV	APOB	1	1	Asian
HBV	Age	0.904809599	1	Asian
HBV	pLMC	0.433288715	0.762267185	Asian
HBV	MDSC	0.381100865	0.716922419	European
HBV	SCNA	0.596304393	0.878277788	European
HBV	Stage	0.43776421	0.763075229	European
HBV	ARID1A	0.129501169	0.374367518	European
HBV	RNAGR	0.710389066	0.924958566	European
HBV	Purity	0.110986229	0.340119089	European
HBV	DEL_4q	0.001178278	0.010660615	European
HBV	APOB	1	1	European
HBV	Age	0.093662771	0.301625873	European
HBV	pLMC	0.764356626	0.925028382	European
MDSC	SCNA	5.35E-24	1.12E-21	TCGA
MDSC	Stage	4.07E-06	3.72E-05	TCGA
MDSC	ARID1A	0.482595044	0.711947474	TCGA
MDSC	RNAGR	2.33E-06	2.45E-05	TCGA
MDSC	Purity	8.85E-16	3.72E-14	TCGA
MDSC	DEL_4q	2.46E-06	2.46E-05	TCGA
MDSC	APOB	0.244648152	0.462847856	TCGA
MDSC	Age	0.357641108	0.573317807	TCGA
MDSC	pLMC	3.21E-07	4.38E-06	TCGA
MDSC	race	2.18E-04	0.001308035	TCGA
MDSC	SCNA	2.90E-10	1.10E-08	Asian
MDSC	Stage	1.62E-04	0.00220197	Asian
MDSC	ARID1A	0.510414779	0.794750507	Asian
MDSC	RNAGR	1.82E-04	0.002300858	Asian
MDSC	Purity	3.07E-07	7.29E-06	Asian
MDSC	DEL_4q	0.059953393	0.223355776	Asian
MDSC	APOB	0.60715944	0.860897714	Asian
MDSC	Age	0.958499216	1	Asian
MDSC	pLMC	0.019305378	0.091700545	Asian
MDSC	SCNA	5.11E-14	9.71E-12	European
MDSC	Stage	0.001778592	0.014692718	European
MDSC	ARID1A	0.640995306	0.908873941	European
MDSC	RNAGR	9.22E-04	0.009217654	European
MDSC	Purity	5.52E-09	3.50E-07	European
MDSC	DEL_4q	1.58E-04	0.001995762	European
MDSC	APOB	0.334654031	0.662336103	European
MDSC	Age	0.611140665	0.886387225	European
MDSC	pLMC	2.98E-05	5.67E-04	European
pLMC	race	0.033150909	0.107102936	TCGA
Purity	DEL_4q	0.961963561	1	TCGA
Purity	APOB	0.735170766	0.882204919	TCGA
Purity	Age	0.544264842	0.756924615	TCGA
Purity	pLMC	0.058754886	0.153414886	TCGA

Purity	race	0.095500082	0.222833524	TCGA
Purity	DEL_4q	0.322559857	0.70074129	Asian
Purity	APOB	0.649322677	0.88645336	Asian
Purity	Age	0.536191209	0.795908825	Asian
Purity	pLMC	0.189930431	0.518662929	Asian
Purity	DEL_4q	0.86765528	0.980190904	European
Purity	APOB	0.48679687	0.801632752	European
Purity	Age	0.234002537	0.540664765	European
Purity	pLMC	0.267328988	0.590610554	European
RNAGR	Purity	1.11E-09	2.12E-08	TCGA
RNAGR	DEL_4q	7.63E-06	6.16E-05	TCGA
RNAGR	APOB	0.340762926	0.563082082	TCGA
RNAGR	Age	3.10E-05	2.10E-04	TCGA
RNAGR	pLMC	0.00978043	0.039497889	TCGA
RNAGR	race	1.26E-10	3.32E-09	TCGA
RNAGR	Purity	3.17E-06	6.69E-05	Asian
RNAGR	DEL_4q	0.126330277	0.393487749	Asian
RNAGR	APOB	0.217948242	0.552135547	Asian
RNAGR	Age	9.95E-04	0.009949335	Asian
RNAGR	pLMC	1.95E-04	0.002310517	Asian
RNAGR	Purity	2.58E-06	7.66E-05	European
RNAGR	DEL_4q	0.001283541	0.011085126	European
RNAGR	APOB	0.932612643	0.996687335	European
RNAGR	Age	0.06599371	0.245858919	European
RNAGR	pLMC	0.024831157	0.124155784	European
SBS22	TMB	1.04E-17	5.44E-16	TCGA
SBS22	DOCK2	0.053350831	0.145502268	TCGA
SBS22	HBV	0.021663875	0.073377643	TCGA
SBS22	MDSC	0.18746157	0.393669297	TCGA
SBS22	SCNA	0.331124586	0.560082558	TCGA
SBS22	Stage	0.975390294	1	TCGA
SBS22	ARID1A	0.423592435	0.649443086	TCGA
SBS22	RNAGR	0.052008294	0.145502268	TCGA
SBS22	Purity	0.05070365	0.143888737	TCGA
SBS22	DEL_4q	0.806317889	0.924533162	TCGA
SBS22	APOB	0.723321532	0.878020357	TCGA
SBS22	Age	0.251742596	0.46373636	TCGA
SBS22	pLMC	4.55E-04	0.002474921	TCGA
SBS22	race	5.57E-07	6.88E-06	TCGA
SBS22	TMB	3.72E-14	7.07E-12	Asian
SBS22	DOCK2	0.688599269	0.914922105	Asian
SBS22	HBV	0.415547713	0.753706474	Asian
SBS22	MDSC	0.661596157	0.891512552	Asian
SBS22	SCNA	0.904499654	1	Asian
SBS22	Stage	0.987416029	1	Asian
SBS22	ARID1A	0.641847856	0.88645336	Asian
SBS22	RNAGR	0.384437657	0.723199553	Asian
SBS22	Purity	0.416521999	0.753706474	Asian
SBS22	DEL_4q	0.121382659	0.38437842	Asian
SBS22	APOB	0.491834736	0.785282352	Asian

SBS22	Age	0.002312458	0.019612461	Asian
SBS22	pLMC	0.005828863	0.038189106	Asian
SBS22	TMB	0.071340683	0.255323224	European
SBS22	DOCK2	0.003466898	0.027446273	European
SBS22	HBV	0.84726704	0.980190904	European
SBS22	MDSC	0.895413555	0.98340217	European
SBS22	SCNA	0.145817739	0.407431917	European
SBS22	Stage	0.746152108	0.925028382	European
SBS22	ARID1A	0.667375209	0.924958566	European
SBS22	RNAGR	0.077759139	0.261306203	European
SBS22	Purity	0.033920441	0.157044702	European
SBS22	DEL_4q	0.608590774	0.886387225	European
SBS22	APOB	0.396822921	0.737293462	European
SBS22	Age	0.312235489	0.631685223	European
SBS22	pLMC	0.035651635	0.157044702	European
SCNA	Stage	0.01024373	0.040588363	TCGA
SCNA	ARID1A	0.810480175	0.924533162	TCGA
SCNA	RNAGR	2.73E-06	2.61E-05	TCGA
SCNA	Purity	7.58E-06	6.16E-05	TCGA
SCNA	DEL_4q	4.61E-10	9.68E-09	TCGA
SCNA	APOB	0.82914216	0.926169434	TCGA
SCNA	Age	0.942367127	1	TCGA
SCNA	pLMC	3.90E-08	6.30E-07	TCGA
SCNA	race	0.056120121	0.151092632	TCGA
SCNA	Stage	0.196545952	0.518662929	Asian
SCNA	ARID1A	0.857788677	1	Asian
SCNA	RNAGR	0.050531024	0.200018638	Asian
SCNA	Purity	0.041315556	0.170651208	Asian
SCNA	DEL_4q	9.39E-06	1.78E-04	Asian
SCNA	APOB	0.935720693	1	Asian
SCNA	Age	0.100350485	0.323162579	Asian
SCNA	pLMC	0.011478071	0.060578706	Asian
SCNA	Stage	0.025900017	0.12617957	European
SCNA	ARID1A	0.886854286	0.980190904	European
SCNA	RNAGR	7.58E-06	1.60E-04	European
SCNA	Purity	1.35E-04	0.001826437	European
SCNA	DEL_4q	6.39E-05	0.001011262	European
SCNA	APOB	0.829487598	0.972855825	European
SCNA	Age	0.035782828	0.157044702	European
SCNA	pLMC	3.22E-06	7.66E-05	European
Shannon	SBS22	0.353462682	0.573317807	TCGA
Shannon	TMB	2.03E-04	0.001251608	TCGA
Shannon	DOCK2	0.795074198	0.924533162	TCGA
Shannon	HBV	0.053215223	0.145502268	TCGA
Shannon	MDSC	0.174553931	0.370265915	TCGA
Shannon	SCNA	0.121523073	0.268629951	TCGA
Shannon	Stage	0.01479539	0.055860754	TCGA
Shannon	ARID1A	0.711400745	0.872194349	TCGA
Shannon	RNAGR	0.403313856	0.634251265	TCGA
Shannon	Purity	7.50E-05	4.92E-04	TCGA

Shannon	DEL_4q	0.018779982	0.065729938	TCGA
Shannon	APOB	0.570306504	0.782773632	TCGA
Shannon	Age	0.904546305	0.98370096	TCGA
Shannon	pLMC	7.21E-09	1.26E-07	TCGA
Shannon	race	0.266796794	0.482994196	TCGA
Shannon	SBS22	0.63507218	0.88645336	Asian
Shannon	TMB	0.487261506	0.784573612	Asian
Shannon	DOCK2	0.860349456	1	Asian
Shannon	HBV	0.083463198	0.288327413	Asian
Shannon	MDSC	0.006275957	0.039747726	Asian
Shannon	SCNA	0.00237414	0.019612461	Asian
Shannon	Stage	0.02215216	0.102656353	Asian
Shannon	ARID1A	0.901367296	1	Asian
Shannon	RNAGR	0.206962866	0.532346774	Asian
Shannon	Purity	0.149867703	0.444919745	Asian
Shannon	DEL_4q	0.004084594	0.029422667	Asian
Shannon	APOB	0.991210562	1	Asian
Shannon	Age	0.512451133	0.794750507	Asian
Shannon	pLMC	8.03E-12	7.62E-10	Asian
Shannon	SBS22	0.69662655	0.924958566	European
Shannon	TMB	1.22E-06	4.63E-05	European
Shannon	DOCK2	0.678484731	0.924958566	European
Shannon	HBV	0.875366661	0.980190904	European
Shannon	MDSC	0.97399729	1	European
Shannon	SCNA	0.82116975	0.969082313	European
Shannon	Stage	0.301621299	0.631685223	European
Shannon	ARID1A	0.732067383	0.924958566	European
Shannon	RNAGR	0.069211677	0.252888821	European
Shannon	Purity	1.73E-04	0.002054917	European
Shannon	DEL_4q	0.312828019	0.631685223	European
Shannon	APOB	0.41421459	0.742460115	European
Shannon	Age	0.769234128	0.925028382	European
Shannon	pLMC	0.009564625	0.064468235	European
Stage	ARID1A	1	1	TCGA
Stage	RNAGR	0.038150754	0.120234078	TCGA
Stage	Purity	0.378073673	0.601480844	TCGA
Stage	DEL_4q	0.908857034	0.98370096	TCGA
Stage	APOB	0.355291023	0.573317807	TCGA
Stage	Age	0.251488938	0.46373636	TCGA
Stage	pLMC	0.818872229	0.924533162	TCGA
Stage	race	0.695847026	0.864661985	TCGA
Stage	ARID1A	0.74681738	0.958752041	Asian
Stage	RNAGR	0.010885911	0.059094943	Asian
Stage	Purity	0.892766769	1	Asian
Stage	DEL_4q	0.65317616	0.88645336	Asian
Stage	APOB	0.866523274	1	Asian
Stage	Age	0.452198835	0.778878106	Asian
Stage	pLMC	0.068625118	0.250745624	Asian
Stage	ARID1A	0.739865628	0.924958566	European
Stage	RNAGR	0.569744855	0.865894794	European

Stage	Purity	0.072565548	0.255323224	European
Stage	DEL_4q	0.887330713	0.980190904	European
Stage	APOB	0.042663491	0.176218766	European
Stage	Age	0.252025201	0.570057003	European
Stage	pLMC	0.047187362	0.190757419	European
TMB	DOCK2	7.36E-04	0.003681903	TCGA
TMB	HBV	0.243928575	0.462847856	TCGA
TMB	MDSC	0.014978099	0.055860754	TCGA
TMB	SCNA	0.058896301	0.153414886	TCGA
TMB	Stage	0.250193351	0.46373636	TCGA
TMB	ARID1A	0.228722331	0.440657702	TCGA
TMB	RNAGR	0.015428208	0.055860754	TCGA
TMB	Purity	0.038360396	0.120234078	TCGA
TMB	DEL_4q	0.30658419	0.545615932	TCGA
TMB	APOB	0.102728334	0.235685582	TCGA
TMB	Age	6.62E-04	0.003473604	TCGA
TMB	pLMC	0.017343974	0.061732788	TCGA
TMB	race	0.005268842	0.023541633	TCGA
TMB	DOCK2	0.004887358	0.033164213	Asian
TMB	HBV	0.381276167	0.723199553	Asian
TMB	MDSC	0.249090771	0.599615865	Asian
TMB	SCNA	0.846328854	1	Asian
TMB	Stage	0.944624387	1	Asian
TMB	ARID1A	0.155378419	0.451873283	Asian
TMB	RNAGR	0.00828092	0.049167961	Asian
TMB	Purity	0.286948741	0.67308964	Asian
TMB	DEL_4q	0.814853285	0.998852414	Asian
TMB	APOB	0.48379099	0.784573612	Asian
TMB	Age	2.77E-05	4.79E-04	Asian
TMB	pLMC	0.007267857	0.044544928	Asian
TMB	DOCK2	0.097898817	0.310012921	European
TMB	HBV	0.551494819	0.858885374	European
TMB	MDSC	0.092209438	0.301625873	European
TMB	SCNA	0.023105864	0.120902985	European
TMB	Stage	0.036368247	0.157044702	European
TMB	ARID1A	0.927841998	0.996687335	European
TMB	RNAGR	0.077211624	0.261306203	European
TMB	Purity	0.113789304	0.343174092	European
TMB	DEL_4q	0.880438165	0.980190904	European
TMB	APOB	0.165492154	0.430733004	European
TMB	Age	0.016258365	0.092746081	European
TMB	pLMC	0.690952359	0.924958566	European

Table S11: Univariate Cox model p-values for selected features

Feature	TCGA p-value	Asian p-value	European p-value	TCGA hazard ratio (HR)	Asian HR	European HR
DEL_16q	0.138852303	0.013678165	0.944278184	1.369969241	2.44338328	0.980854849
GEP	0.004972856	0.045125428	0.049699245	0.376435896	0.327785639	0.400601847
AMP_5p	0.001684711	0.009423369	0.127016511	1.941098337	2.394041284	1.52112789
ARID2	0.100951073	0.021549733	0.842230971	1.898617213	2.904017113	1.155734587
grade	0.933723283	0.048943727	0.028681825	1.446663597	0.36809533	14.75975762
Shannon	0.463951822	0.456490344	0.705370608	1.384495382	1.775859263	1.2316533
SBS22	0.097350046	0.005695298	0.268153591	4.605009904	12.32816344	0.014395778
TMB	6.27E-04	0.007931987	0.035615198	1.088612816	1.084090525	1.091857618
DOCK2	2.91E-06	4.34E-10	0.401389969	3.894697212	7.908068329	1.644557134
HBV	3.44E-04	2.90E-06	0.823372239	0.392817404	0.210908639	1.111388826
MDSC	1.29E-06	2.69E-06	0.012908067	10285.79195	2911905.649	289.4785959
SCNA	0.006013828	0.00858458	0.186341882	1.851235905	2.989302609	1.402517578
Stage	2.37E-05	3.21E-07	0.47118531	2.896326049	7.221507673	1.451461022
ARID1A	0.10756669	0.81382962	0.03295767	1.754521841	1.152809626	2.491580848
RNAGR	0.133921798	0.035666591	0.360534077	2.115935045	2.012747466	1.576541171
Purity	0.034580389	0.354047084	0.038100819	3.476205106	2.616649402	4.558198786
DEL_4q	0.198643327	0.619572518	0.085095733	1.313813536	1.18956011	1.611304186
APOB	0.281133448	0.275742715	0.003198091	1.434911954	0.460820528	2.999843187
Age	0.023641904	0.975036795	0.010827605	1.020235717	1.000492908	1.031843972
pLMC	0.539210596	0.746872136	0.422735079	0.995295165	0.995357222	0.99231304
race	0.333215593	NA	NA	1.236967496	NA	NA
